# Supplementary material for: Impact of urban productive safety net program on urban households’ asset accumulation and food consumption rate in Dessie City, South Wollo Zone, Amhara Region, Ethiopia
Source: PLoS One. 2024 Sep 25;19(9):e0308575. doi: 10.1371/journal.pone.0308575 (PMC11423993; doi:10.1371/journal.pone.0308575)
Supplement: Attachment — Submitted filename: Response to reviewrers.docx [file pone.0308575.s002.docx]

**Response to Reviewers**

**Minor comments**

1.In the abstract, the authors should remove italics from this section.

**Response**: We did it.

2. In the introduction, line 53, the name of the author should be mention before the reference number.

**Response:** Mentioned.

Samuel [2]…

3. Lines 59 &60, approximately 850 million people worldwide faced food insecurity, with a majority residing in developing regions. According to the state of food insecurity (SOFI) 2023 report how many people are food insecure worldwide? What is the share of Africa’s continent?

**Response:** The answer is given as follow:

The relative lack of change in hunger at the global level from 2021 to 2022 hides substantial differences at the regional level. Progress was made towards reducing hunger in most subregions in Asia and in Latin America, but hunger is still on the rise in Western Asia, the Caribbean and all subregions of Africa. The proportion of the population facing hunger is much larger in Africa compared with the other regions of the World – nearly 20 percent compared with 8.5 percent in Asia, 6.5 percent in Latin America and the Caribbean, and 7.0 percent in Oceania [4].

4-Lines 60 &61, In Africa, around 200 million people, accounting for 27.4 percent of the population, experienced food insecurity between the 1970s and 1990s. what is the current prevalence for Africa in recent time?2020-2023?

**Response:**

- An estimated 149 million Africans are facing acute food insecurity—an increase of 12 million people from a year ago. This equates to a risk category of 3 or higher (Crisis, Emergency, and Catastrophe) on the Integrated Food Security Phase Classification (IPC) scale of 1 to 5. Some 122 million of those facing acute food insecurity are in countries experiencing conflict—82 percent of the total—accentuating that conflict is the primary driver of acute food insecurity in Africa [5]. Four of the top 10 countries facing the most acute food insecurity are in East Africa—Sudan, Ethiopia, South Sudan, and Somalia. An estimated 20.1 million people are facing food insecurity in Ethiopia, and 15.1 million require emergency food assistance, following a 2-year war in Tigray, conflicts in other parts of the country, as well as a severe drought that struck the Horn of Africa. The World Food Program and other donors suspended food aid in Ethiopia for 4 months following the discovery of a large-scale diversion within the country [5].

5- Lines 61 & 62, Ethiopia, in particular, had about 8.5 million chronically food insecure individuals, constituting 10 percent of its population. Is this the most recent data available?

**Response:**

- An estimated 20.1 million people are facing food insecurity in Ethiopia, and 15.1 million require emergency food assistance, following a 2-year war in Tigray, conflicts in other parts of the country, as well as a severe drought that struck the Horn of Africa. The World Food Program and other donors suspended food aid in Ethiopia for 4 months following the discovery of a large-scale diversion within the country [5].

6- Lines 65 &66 conducted a study indicating that the second phase of PSNP plays a crucial role in ensuring food security for both chronically and temporarily food insecure households in Ethiopia. Meaning of PSNP? State the full meaning at first mention before abbreviating.

**Response:** corrected.

The Productive Safety Net Program (PSNP)

7- Lines 67 &68 districts across eight regions by providing program .transfers and supporting productive activities until December 2014. What was its success rate and what is it fails out as compared with the initial emergency relief appeal method?

**Response:**

Before implementing PSNP, the Ethiopian 69 government relied on unpredictable annual appeals for emergency food relief, which saved lives but failed to 70 address the increasing number of food insecure individuals [6]. Based on the information provided, the study conducted by Berhane et al. [7] indicates that the second phase of the Productive Safety Net Program (PSNP) in Ethiopia has several successes when compared with the initial emergency relief appeal method. These successes include:

**Enhanced food security**: The study suggests that the PSNP plays a crucial role in ensuring food security for both chronically and temporarily food insecure households. By providing program transfers and supporting productive activities, the PSNP aims to assist over 7.5 million people in 318 districts across eight regions. This targeted approach is likely more effective in addressing the needs of food insecure individuals compared to the unpredictable annual appeals for emergency food relief.

**Long-term planning and stability:** Unlike the initial emergency relief appeal method, which relied on unpredictable annual appeals for food aid, the PSNP operates on a set timeline until December 2014. This longer-term planning allows for more stability and predictability in addressing food insecurity issues.

**Diversification of support:** The PSNP not only provides program transfers but also supports productive activities. This indicates that the program aims to go beyond providing immediate relief by helping households develop sustainable livelihoods and improve their resilience to food insecurity.

However, without further information on the specific failures of the initial emergency relief appeal method, it is challenging to make a direct comparison. It is important to note that the PSNP is a complex program with its own limitations and challenges. Further analysis and evaluation would be needed to understand the full extent of its successes and failures in comparison to the initial emergency relief appeal method.

8- Line 82, million urban poor living in 972 cities and towns.

Urban poor what? dwellers/resident?

**Response:**

The Ethiopian government has been working on a program called the Urban Productive Safety Net Program (UPSNP) that will span a 10-year period. This program is part of the Urban Food Security and Job Creation Strategy, which was officially endorsed on May 8, 2015. The purpose of the UPSNP is to provide assistance to more than 4.7 million impoverished individuals living in 972 cities and towns across the country.

9- Lines 86-88, beneficiaries, In the first phase, 604,000 the poorest 12 percent and about 55 percent of people living below the poverty line in these 11 cities Urban Productive Safety Net Project [10]. Incomplete statement. What happened to 604,000?

**Response:** Corrected as follow:

In the initial stage of the UPSNP, approximately 604,000 individuals who are among the most impoverished 12 percent and around 55 percent of the population residing below the poverty threshold in these 11 cities were the recipients of the program's benefits.

10- Line 93, Having these in mind the researcher was try to evaluate the impacts of urban productive safety net program on urban households’ asset accumulation , consumption and food security status in Dessie City. Delete the word was.

**Response:** Corrected as follow:

Having these in mind, the researchers tried to evaluate the impacts of urban productive safety net program on urban households’ asset accumulation, consumption and food security status in Dessie City.

11- Line 98 Chronically, food insecurity has been one of the most problems of urban poverty, particularly in drought-prone. Please rephrase for clarity.

**Response:** Rephrased as follow:

Historically, urban poverty has consistently faced the significant challenge of food insecurity, especially in drought-prone areas of Ethiopia.

12- Line 100, High proportion of food insecure households, which have been receiving emergency food aids. Remove the word which.

**Response:** Corrected as follow:

High proportion of food insecure households have been receiving emergency food aids.

13- Line 101, Although the emergency assistance was substantial and saved many lives, it was uncertainty of the food aid, …. Do you mean the uncertainty/or inconsistency in availability of food aid?

**Response:** It is the uncertainty of the food aid and corrected as follow:

Although the emergency assistance was substantial and saved many lives, there was uncertainty of the food aid, poor timing of provision and insufficient quantity of aid for the individual’s beneficiary was among the main defects of food aids in the past and creating dependency syndrome on the recipients [13].

14- Lines 107-109, aim of preventing asset depletion at household level while creating assets at the community level (NO REFERENCE).

**Response:** The reference is included (Look at the following statement):

Recognizing this, the government of Ethiopia changed the emergency food-based assistance to multi-year 108 support of PSNP in 2005 that provides cash and food transfers to chronically food insecure households with the aim of preventing asset depletion at household level while creating assets at the community level Devereux and Sabates (2004).

15- Lines 112-113, Some findings state that, PSNP has able to smooth the consumption and protects household’s assets (GRAMMER CHECK for clarity).

**Response:** Checked.

According to Kataru’s [13] research finding, it has been determined that the Productive Safety Net Program (PSNP) is capable of effectively mitigating consumption fluctuations and safeguarding household assets.

16- Line 113, [14] indicated that beneficiary households have managed to 114 significantly improve their food security status by 1.05 months. Mention the authors NAME BEFORE the reference number.

**Response:** Corrected as follow:

Berhane et al. [16] indicated that beneficiary households have managed to significantly improve their food security status by 1.05 months.

17- Lines 114-115, In contrast to these, what proved is that there is no significant difference between, the consumption as well as assets of PSNP participants and non-participants. Please clarify this statement for better understanding.

**Response:** Clarified as follow:

On the contrary, what has been demonstrated is that there is no noteworthy distinction between the consumption and assets of individuals involved in the PSNP program and those who are not [17].

18- Lines 116-118, Moreover, [17], stated that, participation in PSNP reduces the asset size of the participants, due to the dependency behavior of beneficiaries which discourage them to put efforts on their agricultural activities. Do mention the author before reference.

**Response:**

Furthermore, Habitamu [19], stated that, participation in PSNP reduces the asset size of the participants, due to the dependency behavior of beneficiaries which discourage them to put efforts on their agricultural activities.

19- Line 112, levels have brought inconsistent results and far from conclusive,(This statement is not clear).

**Response:** corrected as follow:

The previous ideas indicated that various researchers have examined the effects of the program at both the national and local levels, but their findings have been inconsistent and far from definitive. Consequently, this study was conducted to address this gap.

20- Line 136, THE RESEARCH DESIGN AND SAMPLING. Put this in sentence case.

**Response: 2.2 The Research Design and Sampling**

21- Lines 137-139, Because of survey design helped identify patterns of behavior, activities, people‘s attitude, opinions, habits and any of the variety of social problems that influence the explicability and scaling-up efforts of the program across various contexts. Recast as survey design helps to identify….

**Response:** Reorganized as follow:

The survey design helps to identify patterns of behavior, activities, people’s attitude, opinions, habits and any of the variety of social problems that influence the explicability and scaling-up efforts of the program across various contexts

22- Lines 139-141, Descriptive survey design help to identify the issues had been occurred and could be predetermine certain preventive measures could be adopted. Paraphrase this sentence for clarity and better understanding

**Response:** Paraphrased as follow:

The utilization of a descriptive survey design aids in identifying past occurrences and enables the pre-determination of specific preventive measures that can be implemented.

23- Line 144, The quantitative data were analyzed and interpreted in the numerical data through appropriate. Delete – the words… in the numerical data

**Response:**

The quantitative data were analyzed and interpreted through appropriate statistical tools and cross tabulations were made to facilitate meaningful analysis and interpretations of the research findings.

24- Line 154, stated that target population as all members of a real set of subjects to which a researcher wishes to……. Replace as by are

**Response:**

Creswell [22], stated that all target populations are member of a real set of subjects to which a researcher wishes to generalize the results of the study.

25- Lines 155-156, … The study was targeted each selected *Kebele*, the name of the entire list of household heads were obtained from *Kebele* administration office. They should be corrected grammatically.

**Response:** corrected as follow:

The study specifically focused on each chosen Kebele, and the researcher obtained the complete roster of household heads from the Kebele administration office.

26- Line 157-158, Then on the bases of *Kebele* document there are 338 PWPSNP beneficiary households and 272 eligible non-beneficiary households in Robit *Kebele.* Full meaning

**Response:**

Then on the bases of *Kebele* document there are 338 Public Work-Productive Safety Net Program (PW-PSNP) beneficiary households and 272 eligible non-beneficiary households in Robit *Kebele*.

27- Line 160, beneficiary household heads were used to as sample frames included for the survey (The statement needs to be improved upon grammatically).

**Response:**

… beneficiary household heads were used as sample frames included for the survey.

28- Line 175, In addition to this, 6 key informant interviewees were conducted with purposively.

You mean interview?

**Response:** Yes, I mean interview. Accordingly, it has corrected as follow:

Additionally, interviews were conducted with six key informants, specifically selected Kebele Development Agents, Kebele Administrators, and the head of the UPSNP Task Force.

29- Lines 175-176, ). In addition to this, 6 key informant interviewees were conducted with purposively selected *Kebele* Development Agents, *Kebele* Administrators and head of UPSNP Task Force. You mean interview? Please rephrase for clarity.

**Response:** Yes, I mean interview. Accordingly, it has rephrased as follow:

In addition to this, interviews were conducted with six key informants, specifically selected *Kebele* Development Agents, *Kebele* Administrators, and the head of the UPSNP Task Force members.

30- Line 178, The study was employed both primary and secondary data sources. Delete-was

**Response:** The word was is deleted.

The study employed both primary and secondary data sources.

31- Line184, The study was employed both quantitative and qualitative data analysis techniques. Delete-was

**Response:** The word was is deleted.

The study employed both quantitative and qualitative data analysis techniques.

32- Lines 186 &187, Similarly, qualitative data was gathered through open-ended questionnaires and interview and analyzed through textually to supplement the questionnaire survey. Delete-through

**Response:** The word through is deleted.

Similarly, qualitative data was gathered through open-ended questionnaires and interview and analyzed textually to supplement the questionnaire survey.

33- Lines 189 &190, To understand the distribution of the covariates across the two groups, the quantitative data were analyzed the 190 independent variables through simple descriptive analysis, such as frequencies, and percentages were done to summarized in tables, interpret and conclude the results. please rephrase.

**Response:** Paraphrased as follow:

To assess the distribution of the covariates between the two groups, the quantitative data underwent analysis using descriptive techniques.

34- Line 190, and percentages were done to …..

**Response:** Paraphrased and corrected as follow:

This involves examining the independent variables through methods such as calculating frequencies and percentages, which were then summarized in tables to interpret and draw conclusions from the results.

35- Lines 193 &194, In addition, to the simple descriptive analysis, to estimate the impact of participation in the urban Productive Safety Net program. Delete-in addition to---

**Response:** The phrase in addition to is deleted.

36- Line 198, According to [22], in estimating logit model for the dichotomous (binary) logistic regressions will be used to….

**Response:** Will be is replaced by was.

According to Gujarati [24], in estimating logit model for the dichotomous (binary) logistic regressions was used to achieve this objective, particularly; participation which takes a value of 1 if the household participated in a program and 0 otherwise.

37- Line 249, …by classifying the households’ consumption in to two main categories,

**Response:** It is rewritten as into

… by classifying the households’ consumption into two main categories,

38- Line 254, .. the field survey based on the respondents’ their consumption rate in terms of Ethiopian birr.

**Response:** The word their is deleted.

… the field survey based on the respondents’ consumption rate in terms of Ethiopian Birr.

39- Results and Discussion section (pages 10- 17) should be well written with appropriate comparison with relevant previous research output/published articles and must be well referenced. That is this section should not just be result presentations alone without being adequately discussed.

**Response: we tried to rewrite it with** appropriate comparison with relevant previous studies. For example, 1. a study conducted by Derso et al. [27] supports the result of credit experience variable.

… A study done by Derso et al. [27] supported this result. It indicated that three in four households were experiencing food insecurity and that an appropriate multi-dimensional approach such as the opening of small business enterprises through credit service support may help to diversify income sources and to reduce the dependency ratio.

2. a study done by Alemseged G, Hailu B (2018) as well as by Scho L, Wekesah F, Mohamed S (2014) supported the result of the variable family size … This result is consistent with the studies conducted in Kenya and other rural parts of north Ethiopia by [28,29] found that households with a large family size were more like to be food secure as compared to those with a small family size. The reason may be as the household size becomes larger, the number of economically active members may also increase, which leads to increase their participation in various income-generating activities that could boost their food security than for households with lower family size.

40- Line 474, CONCLUSION. Can you make it precise?

**Response:** Yes, we did it.

Chronic poverty and lack of food are major global problems, particularly in developing regions. In Ethiopia, around 8.5 million people, constituting 10% of the population, suffer from chronic food insecurity, leading to asset selling and consumption restrictions. A study shows that factors like household head's age, family size, and off-farm income activities influence participation in the PW-UPSNP program. Beneficiary households have more assets than eligible non-beneficiary households. The PW-UPSNP program significantly increases food consumption, non-food consumption, and total consumption expenditure for beneficiaries compared to non-beneficiaries.

Econometric analysis reveals positive effects of the PW-UPSNP program on community and home asset accumulation. More PW-UPSNP beneficiaries are food secure compared to non-beneficiaries. The mean food consumption expenditure is significantly higher for PW- UPSNP beneficiaries. The program has a positive and statistically significant impact on food consumption. The second phase of the PSNP program in Ethiopia ensures food security for both chronically and temporarily food insecure households. It aims to assist over 7.5 million people across eight regions through program transfers and productive activities.

Previous emergency food relief efforts were insufficient, leading to the implementation of the PSNP program. It is the largest social protection program in Sub-Saharan Africa and focuses on selected households over several years to achieve sustainable food security. Most studies have focused on rural areas, but the overall conclusion is that the PW-UPSNP program has a positive effect on consumption, asset accumulation, and food security. The program successfully addresses chronic poverty and food insecurity in Ethiopia.

41- Line 534, (pages 20-21) **REFERENCES.** Most of references are outdated. Kindly work on all

**Response:** We tried to up-date some of them. For example, the following references are included.

World Bank (2012) Retrieved from; Washington, D.C: 2012. Managing risk, promoting growth: Developing systems for social protection in Africa‐‐the World Bank’s Africa social protection strategy 2012-2022. [[Google Scholar](https://scholar.google.com/scholar?q=World+Bank+Managing+risk,+promoting+growth%E2%80%AF:+Developing+systems+for+social+protection+in+Africa%E2%80%90%E2%80%90the+World+Bank%E2%80%99s+Africa+social+protection+strategy+2012-2022+2012+Retrieved+from+Washington,+D.C+)]

FAO, IFAD, UNICEF, WFP and WHO (2023) The State of Food Security and Nutrition in the World 2023. Urbanization, agrifood systems transformation and healthy diets across the rural–urban continuum. Rome, FAO. <https://doi.org/10.4060/cc3017en>

5. Africa Center for Strategic Studies (2023) Unresolved Conflicts Continue to Drive Africa’s Food Crisis. africacenter.org/spotlight/unresolved-conflicts-continue-to-drive-africas-food-crisis/

. Berhane G, J Hoddinott, Kumar H, Alemayehu S, Michael T, Yisehac Y et al. (2013) ‘Evaluation of Ethiopia’s Food Security Program: Documenting Progress in the Implementation of the Productive Safety Nets Program and the Household Asset Building Programm’. ESSP II–EDRI REPORT, Washington, D.C: International Food Policy Research Institute.

Devereux S, Sabates-Wheeler R (2004) Transformative social protection. IDS Working Paper, 232. Institute of Development Studies, Brighton, Sussex Bn1 9RE England.

Derso A, Bizuneh H, Keleb A, Ademas A, Adane M (2021) Food insecurity status and determinants among Urban Productive Safety Net Program beneficiary households in Addis Ababa, Ethiopia. *PLOS ONE,*16(9): e0256634.https://doi. org/10.1371/journal.pone.0256634

Alemseged G, Hailu B (2018) Determinants of rural household food insecurity in Northern Ethiopia: An empirical study. *Sustainable Development Africa*, 2018; 20(1):32–63.

Scho L, Wekesah F, Mohamed S (2014) Vulnerability to food insecurity in urban slums: Experiences from Nairobi, Kenya. *Journal of Urban Health*, 2014; 91(6):1098–113.

42.Conclusion/recommendation

**Response:** we have included a brief recommendation.

The UPSNP program has proven to be an effective social protection intervention in combating chronic poverty and food insecurity in urban areas of Ethiopia. Its positive impact on asset accumulation, food consumption, and overall well-being of beneficiary households is evident. Therefore, it is recommended to continue and expand the UPSNP program to reach more vulnerable urban households and further improve their livelihoods. By doing so, Ethiopia can make significant strides towards achieving sustainable development and reducing poverty and food insecurity in urban areas.
