# Supplementary material for: Impact of urban productive safety net program on urban households’ asset accumulation and food consumption rate in Dessie City, South Wollo Zone, Amhara Region, Ethiopia
Source: PLoS One. 2024 Sep 25;19(9):e0308575. doi: 10.1371/journal.pone.0308575 (PMC11423993; doi:10.1371/journal.pone.0308575)
Supplement: Attachment — Submitted filename: PONE-D-23-24451R1-REVEIWERS COMMENT.docx [file pone.0308575.s003.docx]

#### REVIEWER’S COMMENT

Generally, the study titled “Impact of Urban Productive Safety Net Program on Urban Households’ Asset Accumulation and Food Consumption Rate in Dessie City, South Wollo Zone, Amhara Region, Ethiopia” was well-researched, very relevant, scientific, and very informative. The study focused how to achieve the “zero hunger” which is the Sustainable Development Goal 2 in Ethiopia and other sub-Saharan African Countries where food insecurity and hunger is thriving due to poor food production and agricultural practices.

The authors demonstrated excellent mastery of the study. However, few minor corrections are needed before the manuscript to proceeds to the production phase.

Please find below the area that needs clarification and improvement.

In the abstract, the authors should do some grammar checks on some tenses (appropriate use of past tense). And indicate the knowledge gap and/ problem statement.

**Lines 67 &68** An estimated 149 million Africans are facing acute food insecurity—an increase of 12 million people from a year ago.

Lines 78 & 79 Before implementing PSNP, the Ethiopian 69 government relied on unpredictable annual appeals for emergency food relief, which saved lives but failed to 70 address the increasing number of…………………………………

Lines 84 to 89 The study suggests that the PSNP plays a crucial role in ensuring food

security for both chronically and temporarily food insecure households. By providing program

transfers and supporting productive activities, the PSNP aims to assist over 7.5 million people in 318 districts across eight regions. This targeted approach is likely more effective in addressing the needs of food insecure individuals compared to the unpredictable annual appeals for emergency food relief.

Lines 90 to 93 Unlike the initial emergency relief appeal method, which relied on unpredictable annual appeals for food aid, the PSNP operates on a set timeline until December 2014. This longer-term planning allows for more stability and predictability in addressing food insecurity issues.

Lines 126 &127 ]. Hence, Dessie City has been labeled as typical food insecure area despite various food security interventions with PSNP is effective or not……….

Lines 134 &135 Historically, urban poverty has consistently faced the significant challenge of food insecurity,

especially in drought-prone areas of Ethiopia

**Methodology**

##### Lines 296-302 Impact Indicator Variables for Asset Accumulation and consumption

##### The impact of the PSNP is analyzed by dividing the two categories which are the asset building

and consumption expenditure of the PW-PSNP beneficiary and eligible non-beneficiaryhouseholds.

**Outcome Variables for Asset Accumulation:** The first is the home asset (which is the sum of

household goods and consumer durables) and community asset value is also measured based on

the estimation of its market value by respondents in terms of Birr.

Lines 306 to 310 Therefore, in the study, the households have been asked about the quantity as well as the current price of overall households mean real food consumption expenditure per Adult Equivalent (AE) in terms of 24 hours and current non-food consumption expenditure per adult equivalent (AE) in terms of year, at their disposal, during the field survey based on the respondents’ consumption rate in terms of Ethiopian Birr
