# Supplementary material for: Impact of urban productive safety net program on urban households’ asset accumulation and food consumption rate in Dessie City, South Wollo Zone, Amhara Region, Ethiopia
Source: PLoS One. 2024 Sep 25;19(9):e0308575. doi: 10.1371/journal.pone.0308575 (PMC11423993; doi:10.1371/journal.pone.0308575)
Supplement: Attachment — Submitted filename: PONE-D-23-24451_R2_reviewers COMMENT2024.docx [file pone.0308575.s005.docx]

**PLOS ONE**

#### Impact of Urban Productive Safety Net Program on Urban Households’ Asset Accumulation and Food Consumption Rate in Dessie City, South Wollo Zone, Amhara Region, Ethiopia

--Manuscript Draft--

| **Manuscript Number:** | PONE-D-23-24451R2 |
| --- | --- |
| **Article Type:** | Research Article |
| **Full Title:** | Impact of Urban Productive Safety Net Program on Urban Households’ Asset Accumulation and Food Consumption Rate in Dessie City, South Wollo Zone, Amhara Region, Ethiopia |
| **Short Title:** | Impact of Urban Productive Safety Net Program |
| **Corresponding Author:** | Alem-meta Assefa, PhD Wollo University Dessie, ETHIOPIA |
| **Keywords:** | Productive safety net program, asset accumulation, consumption, Dessie City |
| **Abstract:** | In many developing nations, such as Ethiopia, where it frequently accounts for at least 25% of GDP, agriculture is the main driver of economic growth. However, the productivity and quality of the land depend on the methods used in agriculture, necessitating the use of improved techniques such as the Urban Productive Safety Net Program, which supports low-income households and establishes urban safety net mechanisms. While several studies have examined the effects of rural productive safety net programs, the outcomes of similar programs in urban areas are not well documented. This knowledge gap hinders the ability to effectively design and implement urban productive safety net programs. This study's primary goal was to evaluate how Dessie City's urban productive safety net program affected asset accumulation and consumption rates. There was use of both primary and secondary data sources. Eleven2 households, both beneficiaries and non-beneficiaries of the public work Urban Productive Safety Net Program, were chosen at random.  Econometric models, inferential statistics, and descriptive analysis were employed in the analysis. The econometric estimation results indicated a positive relationship between the asset accumulation and consumption of non-beneficiary and beneficiary households in the public work urban productive safety net program and education, access to credit, adult male labor force participation, and extension services.  Participation in the program was also found to have a positive and significant impact on home and community assets. Even so, overall consumption—both food and non- food—as well as the state of food security are improving. Overall, the results showed that beneficiary households have better consumption and food security status, as well as higher levels of home and community assets as a result of program participation.  Keywords: Productive safety net program; asset accumulation; consumption; Dessie City |
| **Order of Authors:** | Alem-meta Assefa, PhD |
| **Response to Reviewers:** | Based on the questions raised by reviewers, I have responded one by one as follow: Author’s Response  Question 1. In the abstract, the authors should do some grammar checks on some tenses (appropriate use of past tense). And indicate the knowledge gap and/ problem statement.  Response: I made the necessary grammar correction and the inclusion of the knowledge gap.  Question 2: Lines 67 & 68 - An estimated 149 million Africans are facing acute food insecurity—an increase of 12 million people from a year ago. Is this a recent information? If yes please indicate the year of reference and source of the information with appropriate referencing.  Response: I include the following reference:  Food and Agriculture Organization of the United Nations. (2023). The State of Food Security and Nutrition in the World 2023. Rome: FAO. |

|  | Question 3: Lines 78 & 79 - Before implementing PSNP, the Ethiopian government relied on unpredictable annual appeals for emergency food relief, which saved lives but failed to address the increasing number of food insecure individuals [6].  Response: Numbers 69 and 70 are deleted because they were written mistakenly. Question 4: Lines 84 to 89 - The study suggests that the PSNP plays a crucial role in ensuring food security for both chronically and temporarily food insecure households. By providing program transfers and supporting productive activities, the PSNP aims to assist over 7.5 million people in 318 districts across eight regions. This targeted approach is likely more effective in addressing the needs of food insecure individuals compared to the unpredictable annual appeals for emergency food relief. No reference to support this fact.  Response: I include the following reference:  Gilligan, D. O., Hoddinott, J., & Seyoum Taffesse, A. (2008). The impact of Ethiopia's Productive Safety Net Programme and its linkages. International Food Policy Research Institute (IFPRI).  Question 5: Lines 90 to 93 - Unlike the initial emergency relief appeal method, which relied on unpredictable annual appeals for food aid, the PSNP operates on a set timeline until December 2014. This longer-term planning allows for more stability and predictability in addressing food insecurity issues. No reference.  Response: I include the following reference:  Berhane, G., Gilligan, D. O., Hoddinott, J., Kumar, N., & Taffesse, A. S. (2014). Can social protection work in Africa? The impact of Ethiopia's Productive Safety Net Programme. Economic Development and Cultural Change, 63(1), 1-26.  Question 6: Lines 126 & 127 - Hence, Dessie City has been labeled as typical food insecure area despite various food security interventions with PSNP is effective or not. Please paraphrase the following statement:  Response: The statement is paraphrased as follow:  Despite the introduction of multiple food security interventions with PSNP, Dessie City has been classified as a typical food insecure area.  Question 7: Lines 134 &135 - Historically, urban poverty has consistently faced the significant challenge of food insecurity, especially in drought-prone areas of Ethiopia. No reference.  Response: I include the following reference:  Zewdie, Yilma Muluken. 2014. "Food Insecurity and Coping Strategies of Urban Poor Households in Addis Ababa." Journal of Economics and Sustainable Development 5(20): 20-30.  Question 8: Lines 296-302 - Impact Indicator Variables for Asset Accumulation and consumption  The impact of the PSNP is analyzed by dividing the two categories which are the asset building and consumption expenditure of the PW-PSNP beneficiary and eligible non- beneficiary households. Reference for this asersion.  Response: I include the following reference: Gebrehiwot, T. and Castilla, C. 2019. "The Role of the Productive Safety Net Programme (PSNP) in Improving Consumption and Asset Accumulation in Rural Ethiopia." Journal of Development Studies 55(5): 685-700.  Question 9: Outcome Variables for Asset Accumulation: The first is the home asset (which is the sum of household goods and consumer durables) and community asset value is also measured based on the estimation of its market value by respondents in terms of Birr. Reference for this tool/ascersion.  Response: I include the following reference: Gilligan, Daniel O., and Hoddinott, John. 2007. "Is There Persistence in the Impact of Emergency Food Aid? Evidence on Consumption, Food Security, and Assets in Rural Ethiopia." American Journal of Agricultural Economics 89(2): 225-242.  Question 10: Lines 306 to 310 - Therefore, in the study, the households have been asked about the quantity as well as the current price of overall households mean real food consumption expenditure per Adult Equivalent (AE) in terms of 24 hours and current non-food consumption expenditure per adult equivalent (AE) in terms of year, at their disposal, during the field survey based on the respondents’ consumption rate in terms of Ethiopian Birr. Reference for the source of this measure/tool?  Response: I include the following reference: Gebrehiwot, Tesfaye, and Van der Veen, Anne. 2015. "Coping with Food Insecurity on a Micro-scale: Evidence from Ethiopian Rural Households." Ecology of Food and Nutrition 54(2): 187-208. |
| --- | --- |
| **Additional Information:** |  |

| **Question** | **Response** |
| --- | --- |
| **Financial Disclosure**  Enter a financial disclosure statement that describes the sources of funding for the work included in this submission. Review the [submission guidelines](http://journals.plos.org/plosone/s/submission-guidelines#loc-financial-disclosure-statement) for detailed requirements. View published research articles from [*PLOS ONE*](http://journals.plos.org/plosone/) for specific examples.  This statement is required for submission and **will appear in the published article** if the submission is accepted. Please make sure it is accurate. | The author(s) received no specific funding for this work. |
| **Unfunded studies**  Enter: *The author(s) received no specific funding for this work.*  **Funded studies**  Enter a statement with the following details:   - Initials of the authors who received each award - Grant numbers awarded to each author - The full name of each funder - URL of each funder website - Did the sponsors or funders play any role in the study design, data collection and analysis, decision to publish, or preparation of the manuscript? - **NO** - Include this sentence at the end of your statement: *The funders had no role in study design, data collection and analysis, decision to publish, or preparation of the manuscript.* - **YES** - Specify the role(s) played. |  |
| * typeset |  |
| **Competing Interests**  Use the instructions below to enter a competing interest statement for this submission. On behalf of all authors, disclose any [competing interests](http://journals.plos.org/plosone/s/competing-interests) that could be perceived to bias this work—acknowledging all financial support and any other relevant financial or non- financial competing interests. | The authors have declared that no competing interests exist. |

| This statement is required for submission and **will appear in the published article** if the submission is accepted. Please make sure it is accurate and that any funding sources listed in your Funding Information later in the submission form are also declared in your Financial Disclosure statement.  View published research articles from  [*PLOS ONE*](http://journals.plos.org/plosone/) for specific examples. |  |
| --- | --- |
| **NO authors have competing interests**  Enter: *The authors have declared that no competing interests exist.*  **Authors with competing interests**  Enter competing interest details beginning with this statement:  *I have read the journal's policy and the authors of this manuscript have the following competing interests: [insert competing interests here]* |  |
| * typeset |  |
| **Ethics Statement**  Enter an ethics statement for this submission. This statement is required if the study involved:   - Human participants - Human specimens or tissue - Vertebrate animals or cephalopods - Vertebrate embryos or tissues - Field research   Write "N/A" if the submission does not require an ethics statement.  General guidance is provided below. Consult the [submission guidelines](http://journals.plos.org/plosone/s/submission-guidelines#loc-guidelines-for-specific-study-types) for detailed instructions. **Make sure that all information entered here is included in the Methods section of the manuscript.** | Not applicable |

| **Format for specific study types**  **Human Subject Research (involving human participants and/or tissue)**   - Give the name of the institutional review board or ethics committee that approved the study - Include the approval number and/or a statement indicating approval of this research - Indicate the form of consent obtained (written/oral) or the reason that consent was not obtained (e.g. the data were analyzed anonymously)   **Animal Research (involving vertebrate animals, embryos or tissues)**   - Provide the name of the Institutional Animal Care and Use Committee (IACUC) or other relevant ethics board that reviewed the study protocol, and indicate whether they approved this research or granted a formal waiver of ethical approval - Include an approval number if one was obtained - If the study involved *non-human primates*, add *additional details* about animal welfare and steps taken to ameliorate suffering - If anesthesia, euthanasia, or any kind of animal sacrifice is part of the study, include briefly which substances and/or methods were applied   **Field Research**  Include the following details if this study involves the collection of plant, animal, or other materials from a natural setting:   - Field permit number - Name of the institution or relevant body that granted permission |  |
| --- | --- |
|  |  |
| **Data Availability**  Authors are required to make all data underlying the findings described fully available, without restriction, and from the time of publication. PLOS allows rare exceptions to address legal and ethical concerns. See the [PLOS Data Policy](http://journals.plos.org/plosone/s/data-availability) and [FAQ](http://journals.plos.org/plosone/s/data-availability#loc-faqs-for-data-policy) for detailed information. | Yes - all data are fully available without restriction |

| A Data Availability Statement describing where the data can be found is required at submission. Your answers to this question constitute the Data Availability Statement and **will be published in the article**, if accepted. |  |
| --- | --- |
| **Important:** Stating ‘data available on request from the author’ is not sufficient. If your data are only available upon request, select ‘No’ for the first question and explain your exceptional situation in the text box. |  |
| Do the authors confirm that all data underlying the findings described in their manuscript are fully available without restriction? |  |
| **Describe where the data may be found in full sentences. If you are copying our sample text, replace any instances of XXX with the appropriate details.**   - If the data are **held or will be held in a public repository**, include URLs, accession numbers or DOIs. If this information will only be available after acceptance, indicate this by ticking the box below. For example: *All XXX files are available from the XXX database (accession number(s) XXX, XXX.*). - If the data are all contained **within the manuscript and/or Supporting Information files**, enter the following: *All relevant data are within the manuscript and its Supporting Information files.* - If neither of these applies but you are able to provide **details of access elsewhere**, with or without limitations, please do so. For example:   *Data cannot be shared publicly because of [XXX]. Data are available from the XXX Institutional Data Access / Ethics Committee (contact via XXX) for researchers who meet the criteria for access to confidential data.*  *The data underlying the results presented in the study are available* | Data is available within the paper and supporting information. |

| *from (include the name of the third party and contact information or URL).*   - This text is appropriate if the data are owned by a third party and authors do not have permission to share the data.   * typeset |  |
| --- | --- |
| Additional data availability information: | Tick here if your circumstances are not covered by the questions above and you need the journal’s help to make your data available. |

Manuscript	[Click here to access/download;Manuscript;Revised Manuscript.docx](https://www2.cloud.editorialmanager.com/pone/download.aspx?id=36601774&guid=b0f7a88f-1f92-4ad1-b200-c1dbb7238692&scheme=1)

## 1 Impact of Urban Productive Safety Net Program on Urban

2 Households’ Asset Accumulation and Food Consumption Rate

## 3 in Dessie City, South Wollo Zone, Amhara Region, Ethiopia

4 ^1^Bezaye Endris, ^2^Alem-meta Assefa

5

6 ^1^Dessie City Municipal office, Dessie, Ethiopia
7 ^2^Department of Geography and Environmental Studies, Wollo University, P.O. Box 1145, Dessie,
8 Ethiopia

9

10

11 Corresponding author’s email: [alemmeta99@yahoo.com](mailto:alemmeta99@yahoo.com)
12 ^1^Wollo University, Department of Geography and Environmental Studies, P.O. Box 1145, Dessie,
13 Ethiopia
14 Corresponding author’s ORCID: [https://orcid.org/0000-0002-1856-](https://orcid.org/0000-0002-1856-2373)

15	[2373](https://orcid.org/0000-0002-1856-2373)

16

17

18

19

20

##### 21 Abstract

22 Agriculture is the driver of economic growth in many developing countries like Ethiopia, where
23 it often represents at least 25% of gross domestic product. But the quality and productivity of the
24 land depends on the way agriculture is practiced. Better agricultural techniques are necessarily
25 including Productive Safety net Program. Urban Productive Safety Net Program is designed to
26 support the poor households and establish urban safety net mechanisms. The main objective of
27 this study was to assess the impact of urban productive safety net program on asset accumulation
28 and consumption rate in Dessie city. Both primary and secondary data sources were used. A total
29 of 112 households were selected based on simple random sampling method among the public
30 work Urban Productive Safety Net Program beneficiary and eligible non-beneficiary households.
31 Descriptive analysis, inferential statistics and econometric models were used for analysis. Based
32 on the econometric estimation results the demographic and socio-economic variables such as
33 education, access to credit, access to extension services and adult male labor force revealed
34 positive relation to public work urban productive safety net program beneficiary and non-
35 beneficiary household’s asset accumulation and consumption. The impact of program
36 participation was also found to be positive and significant for both home asset and community
37 asset. While food and non-food consumption even the total consumption and food security status
38 are also positive. Generally, the result indicated that, due to program participation beneficiary
39 households have higher home asset and community asset and better consumption and food
40 security status.
41 **Keywords:** Productive safety net program; asset accumulation; consumption; Dessie City

42

43

# 44 1. Introduction

45 In Sub-Saharan Africa, numerous individuals continue to experience chronic food insecurity
46 caused by repeated droughts, limited productivity in subsistence agriculture, and widespread
47 poverty [1]. Samuel [2] states that various food security initiatives have been created to address
48 this ongoing issue effectively. Accordingly, in the past two decades we have seen a rapid increase
49 of social protection programs in African countries to alleviate poverty, food insecurity, and
50 vulnerability of poor households [3]. These formal programs aim to assist individuals and
51 households facing chronic food insecurity or temporary financial setbacks by offering income
52 support or alternatives, such as cash and in-kind transfers, subsidies, and labor-intensive public
53 work. The Productive Safety Net Program (PSNP) is a large-scale social protection program
54 implemented in Ethiopia since 2005. It aims to provide assistance to chronically food-insecure
55 households and help them improve their food security and resilience to shocks. The program
56 combines two main components: direct support through cash and/or food transfers to eligible
57 households, and support for public works programs. The cash and food transfers are intended to
58 meet immediate consumption needs, while public works programs provide temporary
59 employment opportunities and help build community assets such as roads, bridges, and irrigation
60 systems.
61 The relative lack of change in hunger at the global level from 2021 to 2022 hides substantial
62 differences at the regional level. Progress was made towards reducing hunger in most subregions
63 in Asia and in Latin America, but hunger is still on the rise in Western Asia, the Caribbean and
64 all subregions of Africa. The proportion of the population facing hunger is much larger in Africa
65 compared with the other regions of the World – nearly 20 percent compared with 8.5 percent in
66 Asia, 6.5 percent in Latin America and the Caribbean, and 7.0 percent in Oceania [4].
67 An estimated 149 million Africans are facing acute food insecurity—an increase of 12 million
68 people from a year ago. This equates to a risk category of 3 or higher (Crisis, Emergency, and
69 Catastrophe) on the Integrated Food Security Phase Classification (IPC) scale of 1 to 5. Some 122
70 million of those facing acute food insecurity are in countries experiencing conflict—82 percent
71 of the total—accentuating that conflict is the primary driver of acute food insecurity in Africa [5].
72 Four of the top 10 countries facing the most acute food insecurity are in East Africa—Sudan,
73 Ethiopia, South Sudan, and Somalia. Ethiopia, in particular, had an estimated 20.1 million people
74 who are facing food insecurity, and 15.1 million require emergency food assistance, following a
75 2-year war in Tigray, conflicts in other parts of the country, as well as a severe drought that struck
76 the Horn of Africa [5]. These individuals lacked consistent access to sufficient food, leading to
77 asset selling and consumption restrictions in order to survive [6].
78 Before implementing PSNP, the Ethiopian 69 government relied on unpredictable annual appeals
79 for emergency food relief, which saved lives but failed to 70 address the increasing number of
80 food insecure individuals [6]. Based on the information provided, the study conducted by Berhane
81 et al. [7] indicates that the second phase of the Productive Safety Net Program (PSNP) in Ethiopia
82 has several successes when compared with the initial emergency relief appeal method. These
83 successes include:
84 **Enhanced food security**: The study suggests that the PSNP plays a crucial role in ensuring food
85 security for both chronically and temporarily food insecure households. By providing program
86 transfers and supporting productive activities, the PSNP aims to assist over 7.5 million people in
87 318 districts across eight regions. This targeted approach is likely more effective in addressing
88 the needs of food insecure individuals compared to the unpredictable annual appeals for
89 emergency food relief.
90 **Long-term planning and stability:** Unlike the initial emergency relief appeal method, which
91 relied on unpredictable annual appeals for food aid, the PSNP operates on a set timeline until
92 December 2014. This longer-term planning allows for more stability and predictability in
93 addressing food insecurity issues.
94 **Diversification of support:** The PSNP not only provides program transfers but also supports
95 productive activities. This indicates that the program aims to go beyond providing immediate
96 relief by helping households develop sustainable livelihoods and improve their resilience to food
97 insecurity.
98 However, without further information on the specific failures of the initial emergency relief
99 appeal method, it is challenging to make a direct comparison. It is important to note that the PSNP
100 is a complex program with its own limitations and challenges. Further analysis and evaluation
101 would be needed to understand the full extent of its successes and failures in comparison to the
102 initial emergency relief appeal method.
103 In an attempt to break the cycle of annual appeal of food aid and achieve an acceptable level
104 of food security at macro (national) and micro (household) level, the government of Ethiopia
105 developed a food security strategy (FSS) in November 1996 [8]. The FSS highlighted government
106 plans to address the causes and effects of food insecurity in Ethiopia. Based on the FSS, the
107 government designed regional food security programs and projects in 2002 [9].
108 The PSNP is currently the largest operating social protection program in Sub-Saharan Africa,
109 outside of South Africa. It differs from previous food-for-work programs, in that it focuses
110 continuously on selected households over several years and in that the explicit objective was that
111 it will eventually be phased out [10].
112 The Ethiopian government has been working on a program called the Urban Productive Safety
113 Net Program (UPSNP) that will span a 10-year period. This program is part of the Urban Food
114 Security and Job Creation Strategy, which was officially endorsed on May 8, 2015. The purpose
115 of the UPSNP is to provide assistance to more than 4.7 million impoverished individuals living
116 in 972 cities and towns across the country. It is envisaged that this will be achieved over a long-
117 term period through a gradual roll-out plan of different phases starting with big cities having a
118 population of over 100,000 people. The proposed Bank support will provide assistance for the
119 first five-year phase of the government program and is targeting 11 major cities, Addis Ababa
120 and one city from each region (Adama, Assayita, Asosa, Dessie, Dire Dawa, Gambella, Hawassa,
121 Harari, Jijiga, and Mekele). In the initial stage of the UPSNP, approximately 604,000 individuals
122 who are among the most impoverished 12 percent and around 55 percent of the population residing
123 below the poverty threshold in these 11 cities were the recipients of the program's benefits [11].
124 Dessie City was one of chronically food insecure cities in Ethiopia. The first phase of the
125 program started in 2017 through direct support and public work by choosing beneficiaries from
126 selected *Kebeles* [12]. Hence, Dessie City has been labeled as typical food insecure area despite
127 various food security interventions with PSNP is effective or not. Having these in mind, the
128 researchers tried to evaluate the impacts of urban productive safety net program on urban
129 households’ asset accumulation, consumption and food security status in Dessie City.
130 Accordingly, the conceptual framework shows several factors; like personal, socio-economic,
131 institutional and bio-physical factors that affect household participation in UPSNP and its impact

132	(Fig 1).

##### 133 Fig. 1 Conceptual Framework of the Study

134 Historically, urban poverty has consistently faced the significant challenge of food insecurity,
135 especially in drought-prone areas of Ethiopia. The responses to food security have conventionally
136 been dominated by emergency food-based intervention. High proportion of food insecure
137 households have been receiving emergency food aids. Although the emergency assistance was
138 substantial and saved many lives, there was uncertainty of the food aid, poor timing of provision
139 and insufficient quantity of aid for the individual’s beneficiary was among the main defects of
140 food aids in the past and creating dependency syndrome on the recipients [13]. For that reason,
141 the past decades of large-scale food aids deliveries have done little to prevent households’ asset
142 depletion, prevent environmental degradation and chronically food insecurity in general, because
143 of ignorance of integrating these aids with natural resource management in the country.
144 Recognizing this, the government of Ethiopia changed the emergency food-based assistance to
145 multi-year support of PSNP in 2005 that provides cash and food transfers to chronically food
146 insecure households with the aim of preventing asset depletion at household level while creating
147 assets at the community level [14].	At national level, there were some works that tried to
148 highlights the impacts of program intervention, particularly PSNP. Unfortunately, most of them
149 have focused on the impacts of PSNP on asset building and consumption in rural areas rather than
150 urban areas. According to Kataru’s [15] research finding, it has been determined that the
151 Productive Safety Net Program (PSNP) is capable of effectively mitigating consumption
152 fluctuations and safeguarding household assets. Berhane et al. [16] indicated that beneficiary
153 households have managed to significantly improve their food security status by 1.05 months. On
154 the contrary, what has been demonstrated is that there is no noteworthy distinction between the
155 consumption and assets of individuals involved in the PSNP program and those who are not [17].
156 Similarly, Andersson et al. [18] indicated that there was no increment in livestock holdings for
157 program participants. Furthermore, Habitamu [19], stated that, participation in PSNP reduces the
158 asset size of the participants, due to the dependency behavior of beneficiaries which discourage
159 them to put efforts on their agricultural activities. But, none of them discussed the magnitude of
160 the impact of PSNP on asset accumulation and consumption on urban households.
161 From the above statements, we understand that various researchers have examined the effects of
162 the program at both the national and local levels, but their findings have been inconsistent and far
163 from conclusive. Consequently, this study was conducted to address this gap.

# 164 2. Materials and Methods

### 165 2.1 Description of the Study Area

166 Dessie City is aged more than a hundred and ten years old and also capital of Dessie Zuria *Wereda*
167 and South administrative Wollo Zone. It is located between 11^0^08’North Latitude and 39^0^38’East
168 Longitude (Fig 2). It is also situated in the north eastern part of the country at 401km away from
169 Addis Ababa, the capital of Ethiopia. The city is placed at the foot of *Tossa* Ridge and is
170 surrounded by and also incorporates a number of hills within its boundary. The average elevation
171 of the city is 2,600 m.a.s.l. Dessie was established in 1893 by King Michael. The city serves as
172 social, economic and political center for the South Wollo Zone and their administration.
173 According to the Central Statistics Agency [20], the total population of the city is approximately
174 250,346 and its annual growth rate is 3.72%. The sex composition of the population is 46.8%
175 male and 53.2% female. The city has 5 sub-cities and its total area is 16,745.82 hectares.

##### 176 Fig. 2 Map of the Study Area

177 177
178 178

### 179 2.2 The Research Design and Sampling

180 The research was conducted through descriptive survey design. The survey design helps to
181 identify patterns of behavior, activities, people’s attitude, opinions, habits and any of the variety
182 of social problems that influence the explicability and scaling-up efforts of the program across
183 various contexts [21]. The utilization of a descriptive survey design aids in identifying past
184 occurrences and enables the pre-determination of specific preventive measures that can be
185 implemented.
186 The study used mixed research approach. Accordingly, both quantitative and qualitative data
187 were collected and analyzed at the same time. Data obtained through interview, questionnaire and
188 observation were analyzed qualitatively. The quantitative data were analyzed and interpreted
189 through appropriate statistical tools and cross tabulations were made to facilitate meaningful
190 analysis and interpretations of the research findings.
191 **Selection of Sample Site:** On the bases of the researchers’ prior knowledge of the area and work
192 place Dawudo Sub-city was selected purposively among the 5 sub-cities of Dessie City. Then, in
193 Dawudo sub-city, Robit and Bahil Amba *Kebeles* were selected by simple random sampling
194 method.

##### 195 Selection of Sample Households

196 Creswell [22], stated that all target populations are member of a real set of subjects to which a
197 researcher wishes to generalize the results of the study. The study specifically focused on each
198 chosen Kebele, and the researcher obtained the complete roster of household heads from the
199 Kebele administration office.
200 Then on the bases of *Kebele* document there are 338 Public Work-Productive Safety Net
201 Program (PW-PSNP) beneficiary households and 272 eligible non-beneficiary households in
202 Robit *Kebele*. While 337 PW-PSNP beneficiary households and 273 eligible non-beneficiary
203 households in Bahil Amba *Kebele,* thus totally 1220 both in PW-UPSNP beneficiary and eligible
204 non-beneficiary household heads were used as sample frames included for the survey. Then after,
205 the researcher used the known formula so as to minimize the number of household heads in the
206 study as a sample. Thus, Kothari [23] sample size determination was employed as shown below:
207 207

z^2^.p.q.N

e^2^(N−1)+Z^2^.p.q

n =

208 Where: n = Sample size
209 z =Standard variation at 95 percent confidence interval (1.96)
210 p = Sample proportion in the target population estimated to have the characteristics being
211 measured (0.03)

212	q = 1-p

213 N =size of the target population is 1220
214 e= the estimate should be within 3 percent of the true value (0.03)
215 215

Therefore, the desired sample is n =	^1.96^2.0.03.(1−0.03).(1220)	= 112.4

0.03	1219 −1 +(1.96) .0.03.(1−0.03)

2(	)	2

216 216

0.03(1.96^2^ x0.97x1220)

n= 0.03(0.03 𝑥 1219+1.96^2^)

3.8416𝑥0.97𝑥1220	= n=

36.57+3.8416

=

4546.14944	=112

40.4116

217	n =**112**

218 Then by using the above formula, 112 household heads were selected as a representative sample.
219 These household heads were selected based on proportional random sampling technique or ratio
220 to the total household heads (Table 1). In addition to this, interviews were conducted with six key
221 informants, specifically selected *Kebele* Development Agents, *Kebele* Administrators, and the
222 head of the UPSNP Task Force members. Finally, both the sample households and the key
223 informants were orally asked to express their agreement for data collection interview. As a result,
224 they expressed their consent verbally.

### 225 2.3 Data Sources and Collection Techniques

226 The study employed both primary and secondary data sources. The primary data were collected
227 from public work UPSNP beneficiary and eligible non-beneficiary household heads through
228 questionnaires as well as key informant interview. While secondary data were collected from
229 different published and unpublished sources of data. Data collection techniques of the study
230 include questionnaires, key informant interview, and field observation.

### 231 2.4 Methods of Data Analysis

232 The study employed both quantitative and qualitative data analysis techniques. Quantitative data
233 were gathered through the close-ended questionnaire and analyzed by using descriptive statistics,
234 inferential statistics and econometric tools using stata computer software. Similarly, qualitative
235 data was gathered through open-ended questionnaires and interview and analyzed textually to
236 supplement the questionnaire survey.
237 To assess the distribution of the covariates between the two groups, the quantitative data
238 underwent analysis using descriptive techniques. This involves examining the independent
239 variables through methods such as calculating frequencies and percentages, which were then
240 summarized in tables to interpret and draw conclusions from the results. Inferential statistical
241 tools, including mean, Chi-square test, one-sample t-test, and paired t-test, were employed to
242 determine the significance of mean values for continuous variables between the households of
243 PW-PSNP beneficiaries and eligible non-beneficiaries. To estimate the impact of household’s
244 participation in the Urban Productive Safety Net program on asset accumulation and consumption
245 expenditure logit model and an econometric model (Propensity score matching method) were
246 used in addition to the simple descriptive analysis.

##### 247 2.4.1 Binary Logistic Regression Model

248 According to Gujarati [24], in estimating logit model for the dichotomous (binary) logistic
249 regressions was used to achieve this objective, particularly; participation which takes a value of
250 1 if the household participated in a program and 0 otherwise. The logit model was selected for
251 this study because the dependent variable is dichotomous in nature and the computation will be
252 easier. For the binary treatment case, where we estimate the probability of participants versus
253 non-participants of UPSNP is addressed as a decision involving binary/dichotomous response
254 variable. The logit model is mathematically formulated as follow:
255 255

Pi =

𝑒𝑍𝑖

zi	**(1)**

1+𝑒

256 Where, Pi -is the probability of participation in the PSNP in relation to explanatory variable
257 ezi- irrational number to the power of Zi
258 Zi = βo + Σ Βi Xi + Ui	**(2)**
259 Zi - a function of n explanatory variable 1, 2…	n )
260 βo - is the intercept
261 Βi- is regression coefficients to be estimated
262 Xi- is pre-intervention characteristics (explanatory variable)
263 Ui- is a disturbance term
264 The probability that the household belongs to non-participant group is given by:
265 265

1-Pi =

1 zi	**(3)**

1+𝑒

266 Then the odd ratio was written as:
267 267

Pi 1−Pi =

1+𝑒𝑧𝑖 _zi 𝑒

1+𝑒

=

𝑧𝑖

**------------------------------------------------------------------------------(4)**

Pi

268 . It is simply the odds ratio in favor of participating in the UPSNP. It is the ratio of the

1−𝐏𝐢

269 probability that the household would participate in the UPSNP to the probability that he/she would
270 not participate in the UPSNP.
271 By taking the natural log of equation (4), the log of odds ratio was written as:
272 272

Li = Ln ( ^Pi^

1−𝐏𝐢

) = Ln ( 𝑒^𝑧𝑖^ ) = Zi = βo + Σ ΒiXi + Ui	(**5**)

273 Li- is the log of the odds ratio in favor of participation in UPSNP which is linear both in Xi and
274 parameters.
275 **2.4.2 Propensity Score Matching (PSM) Method**
276 As placement to participate in PSNP is not random, i.e., an eligible household is deliberately
277 selected based on asset size, vulnerability to shocks and food insecurity. In addition, the baseline
278 survey was not conducted prior to the intervention of the PSNP in the study area. Therefore,
279 propensity score matching method is usually used to assess the impact of a program, in this case,
280 we used to address the impacts of PSNP on asset accumulation and consumption in the beneficiary
281 households.
282 According to Gilligan et al. [25] this model assumes that, “after controlling for all pre-
283 intervention observable household’s and community’s characteristics that are correlated with both
284 program participation and the outcome variable, untreated groups have the same average outcome
285 as the treated group would have had they did not receive the program”. This is the basic idea, to
286 found in group of non-participants those individuals who are similar to the participants in all
287 relevant pre-treatment characteristics X. Hence, the matching approach is one of the best
288 solutions.
289 **Procedures to estimate propensity score:** It is the first step of PSM technique. Rosenbaum and
290 Rubin [26] revealed that matching can be performed conditioning only on P(X) rather than on X,
291 where P(X) = Prob (D=1|X) is the probability of participating in the program conditional on X.
292 According to these authors, if outcomes without the intervention are independent of participation
293 given X, then they are also independent of participation given P(X). Estimating the propensity
294 score involves decision on three choices: what model to be used for the estimation and what
295 variables should be included in this model and matching algorism.

##### 296 Impact Indicator Variables for Asset Accumulation and consumption

297 The impact of the PSNP is analyzed by dividing the two categories which are the asset building
298 and consumption expenditure of the PW-PSNP beneficiary and eligible non-beneficiary
299 households.
300 **Outcome Variables for Asset Accumulation:** The first is the home asset (which is the sum of
301 household goods and consumer durables) and community asset value is also measured based on
302 the estimation of its market value by respondents in terms of Birr.
303 **Outcome variables for consumption**: In order to analyze the impacts of the PSNP on the
304 household’s consumption the study has attempted to examine it’s by classifying the households’
305 consumption into two main categories, because households have consumed different items and
306 due to this reason their value might not the same. Therefore, in the study, the households have
307 been asked about the quantity as well as the current price of overall households mean real food
308 consumption expenditure per Adult Equivalent (AE) in terms of 24 hours and current non-food
309 consumption expenditure per adult equivalent (AE) in terms of year, at their disposal, during the
310 field survey based on the respondents’ consumption rate in terms of Ethiopian Birr.

# 311 3. Results and Discussion

### 312 3.1 Descriptive Analysis of the Dummy Variables Used in Logit

313 **Model**
314 **Sex of the household head:** In the urban area, female headed households are more effective in
315 feeding their family in a sustainable manner from year to year which makes them more likely to
316 be food secure. Because, female headed households are in a better labor input than the males
317 headed in urban area. Based on the respondents result in this study, the sample was composed of
318 both male and female headed households from the total sample households 94 (84%) and 18
319 (16%) were male and female headed household respectively. From the total male headed
320 household respondents, that have been included in the study, 54 (48%) are from the PW-PSNP
321 participant group while the other 40 (36%) are from the non-PSNP participants. In other way
322 female headed households represent about 8 (7%) and 10 (10%) from PW-PSNP participant and
323 non-PSNP beneficiary households respectively. As the result indicates that, in the study area the
324 male headed households were a higher chance to PSNP participants in the public work
325 components of the PSNP. Therefore, as compared to their female headed households’ counterpart
326 male headed households participate more in the PSNP.
327 **Credit Experience**: It is indicating whether the household had credit experience or not. Those
328 who had credit experience can use it to purchase something so that they would manage to escape
329 the liquidity constraints experienced by many urban households in the study area. Moreover, the
330 credit could also serve as a capital source to fund any off-farm activity that the households may
331 undertake indicating that credit experience minimizes the danger of being food unsecured.
332 Amhara Credit and Saving Institution is one of the main and dominant credit suppliers in the
333 study area. Prior to PSNP of the respondents only 11 (10%) PW-PSNP participant and 7 (6%) of
334 non-PSNP participants had taken a loan. Prior to PSNP shows that about 51 (46%) of respondents
335 and 43 (38%) of non-beneficiary respondents had not borrowing credit experience. Access to
336 credit after the implementation of the program indicated that out of the total respondents 38 (34%)
337 of them were public work of PSNP beneficiaries and 33 (29%) non-beneficiaries households
338 received credit, mainly to purchase food item, and improve household asset. It indicates that,
339 higher percent of PSNP public work participants had access to credit compared to non-participant
340 groups. Hence, after the program, from the total respondents of public work PSNP beneficiary
341 have been better and borrowing experience than non-beneficiary household. It indicates that
342 greater percentage of beneficiary households mainly to purchase food item and improve home
343 asset compared to non-beneficiary households. Moreover, the survey result revealed that out of
344 the total respondents with off-farm income, 51 (46%) of them were from the participant group
345 and the 41 (37%) were non-participant households. A study done by Derso et al. [27] supported
346 this result. It indicated that three in four households were experiencing food insecurity and that
347 an appropriate multi-dimensional approach such as the opening of small business enterprises
348 through credit service support may help to diversify income sources and to reduce the dependency
349 ratio. While, without off-farm income, 11 (10%) of them were from the participant group and the
350 others 9 (8%) were found to be non-participant households; regarding access to extension service
351 about 55 (49%) of PSNP in public work participants and 33 (29%) of non-participants were
352 receiving the type of training in 2019/20. But, the remaining 7 (6%) of PSNP participants and 17
353 (15%) non-participants did not receive any type of training. Based on these results, to some extent
354 the extension service was highly supportive to the PSNP beneficiary households when compared
355 with non-beneficiary households. According to the survey result the educational status of the
356 household revealed that 31 (27.6%) of PW-PSNP beneficiary households were literate (able to
357 read and write) and 25 (22%) of non-beneficiary households were literate (able to read and write).
358 While, 31 (27.6%) and 25 (22%) of PW-PSNP beneficiary and PSNP non-beneficiary household
359 heads were illiterate (unable to read and write) respectively. The result indicated that, both
360 illiterate and literate household heads in the respondent were similar in proportion (Table 2).
361 On the other hand, access to remittance the result of the study showed that about 51(45.5%) of
362 the PW-PSNP beneficiary households did not receive any kind of remittance, and the rest of 11
363 (10%) received remittance mainly to purchase food item and none food items. while	from the
364 total none beneficiary households, 11 (10%) household heads who took remittances and the
365 remaining 39 (35%) non-beneficiary household heads did not received remittance respectively.
366 This means greater percentages of beneficiary of the UPSNP are not received remittance as
367 compared to non-beneficiary. Besides, the result of the study also revealed that food gap faced
368 households prior to the introduction of the program three month and above were faced food gap,
369 58 (52%) and 48 (43%) of PW-PSNP beneficiary and non-PSNP beneficiary households’ groups
370 had food gap on three months of experienced food gaps respectively. It implies that prior to the
371 introduction of the program higher percentage of participant groups were faced food gap and food
372 insecure compared to non-beneficiary households (Table 2 above).

### 373 3.2 Descriptive Analysis of the Continuous Variables Used in Logit

374 **Model**
375 **Age of the household head:** It is a continuous variable that refers to the level of `experience the
376 farmers possess and measured in the number of years. The older the head of the household is the
377 more he/she would be effective in risk diversification as well as in predicting the weather and
378 applying the right technology. However, the existence of large number of children under the age
379 of 15 and old age of 65 and above in the family affect the household consumption negatively. The
380 households with higher dependency ratio will have lowest welfare status. This means households
381 with higher dependency ration had low level of welfare, hence higher probability to fall in to food
382 in security and poverty. The result of the study indicated that the average age of the household
383 head for the whole sample is around 49.17 years (Table 3). When we compare the two groups in
384 terms of their mean age, it was found that treated groups are older than their controlled groups by
385 about 1.58 year, i.e., 49.91 years compared to 48.33 years respectively. Thus, the older household
386 heads were found to be food in secured.
387 **Dependency Ration:** The dependency ratio shows the ratio of economically active persons
388 compared to economically dependent household members. Economically active members of
389 households, whose age is from 14 to 64 were assumed to be the principal productive force and
390 sources of income for the household. Household members whose age was between 0-14 and
391 above 64 were considered as economically inactive and dependent members of the household.
392 Similarly, the dependency ratio for the members of the sampled households estimated to be 1.25
393 and 1.08 of the PW-UPSNP beneficiaries and non-UPSNP beneficiaries respectively. Whereas,
394 the average difference between treated and non-treated group are around 0.32. Therefore, the
395 result indicates that treated groups are the higher dependency than control groups. Thus, the
396 higher dependency contributes to food insecurity and the probability level of participation
397 significantly 1 percent (Table 3 above).
398 **Household Family Size:** It refers to the total number of the household’s member. Family size is
399 one of the major socioeconomic variables, which as a significant influence on the amount of
400 household consumption. As the number of the family increases, given the fixed resources, the
401 food share goes to the individual in that family declines which maximizes the probability of being
402 food insecure. As presented in Table 3, the average family size of the combined samples were
403 around 4. The average family size of beneficiary and non-beneficiary households was 4 and 3
404 respectively. As a result, beneficiary households had greater family size compared to non-
405 beneficiary households (Table 3 above).

### 406 3.3 Impact of Productive Safety net Program on Community and

407 **Home asset values**
408 Different types of sustainable productive activities were being undertaken through public works
409 component of PSNP to build community assets. These activities largely focused on the
410 households’ area of community asset like maintenance of roads, maintenance and construction of
411 schools, maintenance and construction of latrine and cleaning surrounding environment.
412 Whereas, home asset (consumer and durable assets like radio, wrist watch, bed, mobile phone,
413 Tape recorder, Television and housing condition) were included. Then, the result of the study
414 revealed that in the households’ area of community asset and home asset value calculated as the
415 beneficiary worked and contribute as estimated at the values have 345.6 and 711.6 Ethiopian Birr
416 respectively. Whereas, non-beneficiary households of community asset and home asset value
417 contribute to have 291.1 and 254.6 Ethiopian Birr respectively. Therefore, the beneficiary
418 households had greater amount of community and home asset building tendency than non-
419 beneficiary households (Table 4). According to this finding, the mean difference in community
420 asset and home asset value contribute between the beneficiary and non-beneficiary households
421 were found to be positive and statistically significant at 1% of probability level. This implies that,
422 the PSNP beneficiary households possess community asset and home asset value that have
423 contribute to greater value (quality) as compared to the assets possessed by the non-PSNP
424 beneficiary households.

### 425 3.4 Impact of Productive Safety Net Program on Consumption

426 Consumption expenditure was used as impact indicator while evaluating impact of the UPSNP,
427 and it was computed as per adult equivalent consumption expenditure. The overall households
428 mean real consumption expenditure per adult equivalent (AE) for the sample households was
429 257.63 Ethiopian birr. The mean consumption expenditure for PSNP beneficiaries and non-
430 beneficiary groups was 295.21 and 215.54 Ethiopian Birr respectively. The mean difference test
431 of consumption expenditure for the two groups was 79.68 Ethiopian Birr. It implies that
432 statistically significant at 1% probability level. Whereas the overall households mean real food
433 consumption and non-food consumption expenditure per adult equivalent (AE) for the sample
434 households was 209.95 and 47.68 Ethiopian Birr respectively (Table 5). The mean food
435 consumption expenditure for PSNP beneficiary and non-beneficiary groups was also 237.72 and
436 178.86 Ethiopian Birr respectively. The mean difference test of consumption expenditure for the
437 two groups was 58.86 Ethiopian Birr. It implies that statistically significant at 1% probability
438 level. Moreover, the mean non-food consumption expenditure for PSNP beneficiary and non-
439 beneficiary groups were 57.49 and 36.68 Ethiopian Birr respectively. Therefore, the result of the
440 study showed that the PSNP beneficiary households were found to be positive and statistically
441 significant at 1% over the intervention period. Besides, the overall consumption per adult
442 equivalent for the participants’ increased when compared to non-participants over the intervention
443 period. This result was supported by the evidences that taken from the key informants at the time
444 of interview. One of the key informants said that:
445 *“The beneficiary households have better money to pay for food items, medical cost and school fee with the help of*
446 *PW-UPSNP. Before they included in PSNP, some of the beneficiary households were eating once a day but some*
447 *others were eating twice in a day. After they were included in the PW-UPSNP, the majority of the beneficiary*
448 *households are eating three times in a day”.*
449 449

### 450 3.5 Econometric Results

451 The study used propensity score matching to evaluate the effects of PSNP on asset accumulation
452 and consumption expenditure among urban households. The propensity score was estimated using
453 a logistic model based on observable characteristics to determine the likelihood of households
454 participating in the program. Beneficiary and non-beneficiary households were then matched
455 based on their propensity scores, using a binary outcome variable (1 for PW-PSNP beneficiary
456 households and 0 for eligible non-PSNP beneficiary households) in the logit model.

##### 457 3.5.1 Factors of Participation in Productive Safety Net Program

458 The researchers have incorporated relevant variables to explain the dependent variable and
459 conducted a logit model. They found that the gender, age of the household head, family size, and
460 off-farm income activities significantly affected program placement during selection.
461 The findings show that various factors significantly affected participation in PW-PSNP.
462 Employment on wage, family size, number of adult females, age of household head, and
463 employment on own business before UPSNP intervention all had positive and significant
464 influences on PW-PSNP participation, with varying magnitudes of impact.
465 Households with larger family sizes are more likely to participate in the program compared to
466 those with smaller family sizes. This is because larger families often have higher food demand
467 and a higher chance of food insecurity due to limited resources. The study found family size to
468 be statistically significant at a 5% level of significance. This result is consistent with the studies
469 conducted in Kenya and other rural parts of north Ethiopia by [28,29] found that households with
470 a large family size were more like to be food secure as compared to those with a small family
471 size. The reason may be as the household size becomes larger, the number of economically active
472 members may also increase, which leads to increase their participation in various income-
473 generating activities that could boost their food security than for households with lower family
474 size. Additionally, male-headed households were more likely to be program participants
475 compared to female-headed households, likely due to females being active in non-farm activities
476 generating off-farm income. This result was also statistically significant at a 5% level of
477 significance.
478 Household head age is the other variable which is found to be negatively significant at 1% level
479 of significant and related with probability of participation in PW-PSNP. This implies that as
480 increase in age of household heads, decreases their participation in the program. This is due to
481 the reason that, the age goes beyond certain periods; the person may become physically weak and
482 food insecure. On the other hand, variables like sex of household head, food gap, accesses to
483 extension services and access to remittance and number of adult males are found to be statistically
484 insignificant (Table 6).
485 **3.5.2 Impact of UPSNP on Household Asset Accumulation**
486 In this study, household home assets represent the sum of household goods like home, pot, oven,
487 bed, box and barrel and consumer durables like mobile phone, watch, tape recorder and Radio
488 and Television etc. After converting both the household goods and the consumer durable of the
489 entire households in to their respective market value, the two categories have been added together
490 to form the household home assets.
491 The impact of UPSNP on household’s home asset were found to be positive with the maximum
492 and the minimum ATT value calculated to be 494 Ethiopian Birr (based on NN matching) and
493 441 Ethiopian Birr (based on Kernel matching) (Table 7) respectively. Statistically, this result is
494 found to be significant at one percent for all matching estimators used in this study, i.e., based on
495 Kernel, Stratification and NN matching. The implication of this result is, because of the
496 introduction of the program, the value of non-productive/home asset at the hands of the PW-PSNP
497 beneficiary households is higher than the value of the non-productive asset at the hands of the
498 non-beneficiary households by the amount of at maximum 494 Ethiopian Birr and at minimum
499 441 Ethiopian Birr. And this result confirms the fact that the program is successful in avoiding
500 the depletion in the non-productive asset of the beneficiary households.
501 According to this finding, the program has a positive and significant impact on the household
502 asset at 1% level of significance. This can be also stated as PW-PSNP beneficiary households
503 have more asset value than the eligible non-beneficiary households. Generally, the above positive
504 value of ATT implies that, because of the program intervention household’s home assets have
505 been protected in the study area.
506 During the field survey, the participants of the PW-PSNP program made efforts to enhance
507 community assets, as evidenced by their contributions to activities such as road maintenance,
508 improving the greenery of the environment, and erecting fences around schools, health posts, and
509 ponds. The result of this study shows that the current value of community assets at the hands of
510 the PSNP beneficiary households is higher than the current value of community assets at the hand
511 of the non-PSNP beneficiary households.
512 The mean difference in the current value of community assets between the PW-PSNP
513 beneficiary and eligible non-beneficiary households were found to be positive and significant
514 with the minimum and maximum ATT value of contribute as calculated 54 Ethiopian Birr (based
515 on Stratification matching) and 82 Ethiopian Birr (based on the NN matching) respectively.
516 Statistically, the calculated ATT for the treated and control group is found to be significant at 1%
517 based on Kernel and Stratification matching while 5% with nearest neighbor matching estimators
518 used in the study.
519 According to the result of this study on average the community assets of the PW-PSNP
520 beneficiary households contribute to the value at maximum 82 Ethiopian Birr (based on NN
521 matching) and to the minimum 54 Ethiopian Birr (based on Stratification matching) more as
522 compared to the community assets of the eligible non- beneficiary households.
523 In general, because of their participation in the program the PW-PSNP beneficiary households
524 have managed to protect their community assets from possible deterioration. Thus, the program
525 enables the PSNP beneficiary households to preserve their community assets from depletion. The
526 result coincides with [25], who assessed the impact of Ethiopia’s PSNP using the method of
527 propensity score matching method based on various impact indicators, also found that the mean
528 difference in community assets between PW-PSNP and eligible non-PSNP households are
529 positive and significant at one percent. Therefore, the result of the study showed that participation
530 in PW-PSNP was found to be helpful to the participants in improving their community assets.
531 This result is also coinciding with the finding of Yibrah [30], who studied the impact of
532 participating in the Productive Safety Net Program on Consumption and Asset holding.

#### 533 3.5.3 Food Security Status of Sample Households

534 Data on food consumption was collected from 112 households for this study, aiming to capture
535 the diversity and frequency of various foods consumed within a 24-hour period. The study utilized
536 a household food consumption score, which is a continuous measure and can be divided into three
537 levels. The results indicated that 45 households (72.5%) of PW-UPSNP beneficiaries had
538 acceptable food consumption, 11 households (17.7%) had borderline consumption, and 6
539 households (9.6%) were considered poor in terms of their food consumption score. On the one
540 hand, among	eligible	non-UPSNP	beneficiary	households, 38%	had	acceptable	food
541 consumption, 36% had borderline consumption, and 26% had poor consumption. The households
542 with poor consumption are considered food insecure, while those with borderline consumption
543 are moderately food insecure, and the households with adequate food consumption are food
544 secure. On the other hand, the mean food consumption score for PW-UPSNP beneficiary
545 households was 1.53, whereas for eligible non-PSNP beneficiary households, it was 1.23.
546 Consequently, there is a significant difference in the mean food consumption score between PW-
547 UPSNP beneficiary households and eligible non-UPSNP beneficiary households, which amounts

548	to 1.38.

549 The outcome suggests that households benefiting from PW-UPSNP are more food secure
550 compared to those eligible for PSNP but not receiving PW-UPSNP. The chi-square test confirms
551 a significant difference in food security status between the two groups at a 1% level of
552 significance (Table 8).

#### 553 3.5.4 Food Consumption Expenditure Per Adult Equivalent

554 Food consumption expenditure behavior can be used to assess household food security. The
555 average food consumption expenditure for households benefiting from PW-UPSNP was 237.72
556 Ethiopian Birr, while for non-beneficiary households, it was 178.86 Ethiopian Birr. The
557 statistically significant difference of 58.86 Ethiopian Birr between the two groups was observed
558 at a 1% probability level (Table 9). Moreover, this result indicates that the PW-UPSNP on
559 beneficiary households food consumption had a positive with the maximum and the minimum
560 ATT value calculated to be 59 Ethiopian Birr (based on stratification matching) and 45 Ethiopian
561 Birr (based on NN matching) respectively (Table 10).
562 562
563 563

# 564 Conclusions

565 Chronic poverty and lack of food are major global problems, particularly in developing regions.
566 In Ethiopia, around 8.5 million people, constituting 10% of the population, suffer from chronic
567 food insecurity, leading to asset selling and consumption restrictions. A study shows that factors
568 like household head's age, family size, and off-farm income activities influence participation in
569 the PW-UPSNP program. Beneficiary households have more assets than eligible non-beneficiary
570 households. The PW-UPSNP program significantly increases food consumption, non-food
571 consumption, and total consumption expenditure for beneficiaries compared to non-beneficiaries.
572 Econometric analysis reveals positive effects of the PW-UPSNP program on community and
573 home asset accumulation. More PW-UPSNP beneficiaries are food secure compared to non-
574 beneficiaries. The mean food consumption expenditure is significantly higher for PW-UPSNP
575 beneficiaries. The program has a positive and statistically significant impact on food
576 consumption. The second phase of the PSNP program in Ethiopia ensures food security for both
577 chronically and temporarily food insecure households. It aims to assist over 7.5 million people
578 across eight regions through program transfers and productive activities.
579 Previous emergency food relief efforts were insufficient, leading to the implementation of the
580 PSNP program. It is the largest social protection program in Sub-Saharan Africa and focuses on
581 selected households over several years to achieve sustainable food security. Most studies have
582 focused on rural areas, but the overall conclusion is that the PW-UPSNP program has a positive
583 effect on consumption, asset accumulation, and food security. The program successfully
584 addresses chronic poverty and food insecurity in Ethiopia.
585 585
586 586

| 587 | **Supporting information** |  |
| --- | --- | --- |
| 588 | S1_Fig 1 |  |
| 589 | S1_Table 1 |  |
| 590 | S2_Table 2 |  |
| 591 | S3_Table 3 |  |
| 592 | S4_Table 4 |  |
| 593 | S5_Table 5 |  |
| 594 | S6_Table 6 |  |
| 595 | S7_Table 7 |  |
| 596 | S8_Table 8 |  |
| 597 | S9_Table 9 |  |
| 598 | S10_Table 10 |  |
| 599 | **Acknowledgments** |  |
| 600 | The authors would like | to thank farmers, agricultural development agents, and local |

601 administrators of the study area for their assistance during the field work.

##### 602 Declarations

603 The authors declare that they have no conflict of interests.

##### 604 Ethics Statement

605 As I mentioned in the Journal web site the reason why I gave the response N/A is because our
606 study does not have any connection with both human and animal connection. What we did is
607 collecting data from sample households via interview and focus group discussion as well as field
608 observation. All of these data collection methods do not require any kind of ethical approval. Both
609 the sample households and the key informants were expressed their consent verbally to give us
610 the response for the questions we asked about the impact of urban productive safety net program
611 on urban households’ asset accumulation and food consumption rate in Dessie City. Their verbal
612 agreement is mentioned in the methodology section of the manuscript.

##### 613 Competing Interests

614 The authors have declared that no competing interests exist.

##### 615 Data Availability Statement

616 Data is available within the paper and supporting information. We attached minimal data set
617 within the Supporting Information.

##### 618 Funding

619 The author(s) received no specific funding for this work.

##### 620 Author Contributions

621 Conceptualization: Bezaye Endris, Alem-meta Assefa.
622 Data curation: Bezaye Endris, Alem-meta Assefa.
623 Formal analysis: Bezaye Endris, Alem-meta Assefa.
624 Investigation: Bezaye Endris, Alem-meta Assefa.
625 Methodology: Bezaye Endris, Alem-meta Assefa.
626 Project administration: Bezaye Endris, Alem-meta Assefa.
627 Resources: Bezaye Endris, Alem-meta Assefa.
628 Software: Bezaye Endris, Alem-meta Assefa.
629 Supervision: Bezaye Endris, Alem-meta Assefa.
630 Validation: Bezaye Endris, Alem-meta Assefa.
631 Visualization: Bezaye Endris, Alem-meta Assefa.
632 Writing – original draft: Bezaye Endris, Alem-meta Assefa.
633 Writing – review and editing: Bezaye Endris, Alem-meta Assefa.
634 634
635 635
636 636
637 637
638 638
639 639
640 640
641 641
642 642
643 643
644 644
645 645
646 646
647 647
648 648
649 649
650 **REFERENCES**
651 1. Fekadu N, Mberengwa (2009) Challenges of Productive Safety Net Program Implementation 652	at Local Level. The Cases of Kuyu Woreda, North Shewa Zone, Oromia Region, Ethiopia. 653	*Journal of Sustainable Development in Africa,* 11(1), 248-267.

654	2. Samuel G (2010) Food aid and small householder agriculture in Ethiopia development studies,

655	March 20-22.

656	3. World Bank (2012) Retrieved from; Washington, D.C: 2012. Managing risk, promoting 657	growth: Developing systems for social protection in Africa‐ ‐ the World Bank’s Africa 658	social protection strategy 2012-2022. [[Google Scholar](https://scholar.google.com/scholar?q=World%2BBank%2BManaging%2Brisk%2C%2Bpromoting%2Bgrowth%E2%80%AF%3A%2BDeveloping%2Bsystems%2Bfor%2Bsocial%2Bprotection%2Bin%2BAfrica%E2%80%90%E2%80%90the%2BWorld%2BBank%E2%80%99s%2BAfrica%2Bsocial%2Bprotection%2Bstrategy%2B2012-2022%2B2012%2BRetrieved%2Bfrom%2BWashington%2C%2BD.C%2B)]

659 4. FAO, IFAD, UNICEF, WFP and WHO (2023) The State of Food Security and Nutrition in the 660	World 2023. Urbanization, agrifood systems transformation and healthy diets across the 661	rural–urban continuum. Rome, FAO. <https://doi.org/10.4060/cc3017en>

662	5. Africa Center for Strategic Studies (2023) Unresolved Conflicts Continue to Drive Africa’s 663	Food Crisis. africacenter.org/spotlight/unresolved-conflicts-continue-to-drive-africas-food- 664	crisis/

665	6. Ministry of Agriculture and Rural Development (MOARD) (2010) Concept Note for

666	Establishment of a System of Productive Safety Net in Ethiopia.

667 7. Berhane G, J Hoddinott, Kumar H, Alemayehu S, Michael T, Yisehac Y et al. (2013) 668	‘Evaluation of Ethiopia’s Food	Security Program: Documenting Progress	in	the 669	Implementation of the Productive Safety Nets Program and the Household Asset Building 670	Programm’. ESSP II–EDRI REPORT, Washington, D.C: International Food Policy 671	Research Institute.

672	8. Federal Democratic Republic of Ethiopia (FDRE) (2006) Productive Safety Net Program: 673	Program Implementation Manual(rev). Ministry of Agriculture and Rural Development 674	(MoARD), Addis Ababa, Ethiopia.

675	9. Federal Democratic Republic of Ethiopia (FDRE) (2001) Rural Development Policies,

676	Strategies and Instruments of the Government of the FDRE.

677	10. Berhane G, J Hoddinott, Kumar N, Alemayehu S (2011) The impact of Ethiopia’s Productive 678	Safety Nets and Household Asset Building Programme:2006-2010. Washington, D.C: 679	International Food Policy Research Institute.

680	11. Ministry of Urban Development and Housing UPSNP (2016) Urban Productive Safety Net

681	Program Implementation Manual, Version xx, Addis Ababa.

682	12. Dessie City UPSNP Task Force Office (2019) Unpublished Report Document, Ethiopia.

683	13. Andersson C, Alemu M, Stage J (2010) Impacts of the Productive Safety Net Program in 684	Ethiopia on livestock and tree holdings of rural households Environment for Development 685	Discussion Paper Series 09-05.

686	14. Devereux S, Sabates-Wheeler R (2004) Transformative social protection. IDS Working Paper,

687	232. Institute of Development Studies, Brighton, Sussex Bn1 9RE England.

688

689 15. Kataru K (2011) Assessment of the Impact of Productive Safety Net Program on the Lives of 690	the Beneficiaries in Ethiopia: The Case of Dera Malo Woreda, Gamo Gofa Zone, 691	unpublished Master of Arts Degree in Public Management, Addis Ababa University, 692	Ethiopia.

693 16. Berhane G, J Hoddinott, Kumar N, Alemayehu S (2011) The impact of Ethiopia’s Productive 694	Safety Nets and Household Asset Building Programme:2006-2010. Washington, D.C: 695	International Food Policy Research Institute.

696	17. Tadele M (2011) Impact of Productive Safety Net Program on the sustainable Land

697	Management Practice in Central Rift value. University of Haramaya, Ethiopia.

698	18. Andersson C, Alemu M, Jesper S (2011) Impacts of the Productive Safety Net Program in 699	Ethiopia on livestock and tree holdings of rural households. *Journal of Development* 700	*Economics*, 94 (2011) 119–126. doi:10.1016/j.jdeveco.2009.12.002.

701 19. Habitamu A (2011) Impact of Productive Safety Net Program on the Household Welfare and
702 labor supply in rural Ethiopia. Master’s Thesis, Addis Ababa University, Ethiopia.
703 20. Central Statistics Authority (CSA) (2007) National Population Statistics. Federal Democratic
704 Republic of Ethiopia, Central Statistical Authority, Addis Ababa, Ethiopia.
705 21. Kombo DK, Tromp DL (2006) *Thesis Writing*: An Introduction, Paulines Publications Africa,
706 Nairobi.
707 22. Creswell JW (2009) *Research Design: Qualitative, Quantitative, and Mixed Methods*
708 *Approaches.* 3^rd^Edition, SAGE Publications, London, United Kingdom.
709 23. Kothari CR (2004) *Quantitative Techniques*. 2^nd^Edition, New Delhi: Vikas Publishing House
710 Pvt. Ltd. India.
711 24. Gujarati DN (2004) *Basic Econometrics*. 4^th^Edition, the McGraw−Hill Companies.
712 25. Gilligan DO, Hoddinott J, Alemayehu S (2008) *An analysis of Ethiopia’s Productive Safety* 713	*Net Program and its linkages*. International Food Policy Research Institute, Washington, 714	DC.

715	26. Rosenbaum PR, Rubin DB (1983) The Central Role of the Propensity Score in

716	Observational Studies for Causal Effects. *Biometrika*,70,41-55.

717	<http://dx.doi.org/10.1093/biomet/70.1.41>

718	27. Derso A, Bizuneh H, Keleb A, Ademas A, Adane M (2021) Food insecurity status and 719	determinants among Urban Productive Safety Net Program beneficiary households in 720	Addis	Ababa, Ethiopia. *PLOS	ONE,*16(9): e0256634.https://doi. 721	org/10.1371/journal.pone.0256634

722	28. Alemseged G, Hailu B (2018) Determinants of rural household food insecurity in Northern

723	Ethiopia: An empirical study. *Sustainable Development Africa*, 2018; 20(1):32–63. 724	29. Scho L, Wekesah F, Mohamed S (2014) Vulnerability to food insecurity in urban slums: 725	Experiences from Nairobi, Kenya. *Journal of Urban Health*, 2014; 91(6):1098–113.

726	30. Yibrah H (2010) Impact of Productive Safety Net Program on the Rural Households asset

727	Protection and Consumption. Unpublished MA Thesis, Mekelle University, Ethiopia.

728

[Click here to access/download;Figure;Fig.1.tif](https://www2.cloud.editorialmanager.com/pone/download.aspx?id=36601775&guid=69eb68ec-3dd4-47fa-9036-5ec835037f92&scheme=1)

Figure	[Click here to access/download;Figure;S1_Fig 1.tif.tif](https://www2.cloud.editorialmanager.com/pone/download.aspx?id=36601776&guid=2635f3ff-9d12-43e0-8bfe-2f69bc82bbd7&scheme=1)

[Click here to access/download](https://www2.cloud.editorialmanager.com/pone/download.aspx?id=36601777&guid=56292ee4-c1cb-4a25-83c8-6f52c1773257&scheme=1)

[**Supporting Information** Table 1.docx](https://www2.cloud.editorialmanager.com/pone/download.aspx?id=36601777&guid=56292ee4-c1cb-4a25-83c8-6f52c1773257&scheme=1)

[Click here to access/download](https://www2.cloud.editorialmanager.com/pone/download.aspx?id=36601778&guid=b2f8614d-7bce-41ba-a3a2-1fad55cd849e&scheme=1)

[**Supporting Information** Table 2.docx](https://www2.cloud.editorialmanager.com/pone/download.aspx?id=36601778&guid=b2f8614d-7bce-41ba-a3a2-1fad55cd849e&scheme=1)

[Click here to access/download](https://www2.cloud.editorialmanager.com/pone/download.aspx?id=36601779&guid=96d76f35-6254-4f48-836c-8854245d438b&scheme=1)

[**Supporting Information** Table 3.docx](https://www2.cloud.editorialmanager.com/pone/download.aspx?id=36601779&guid=96d76f35-6254-4f48-836c-8854245d438b&scheme=1)

[Click here to access/download](https://www2.cloud.editorialmanager.com/pone/download.aspx?id=36601780&guid=4c6cb1c5-8562-456d-b03a-1ce6f3950674&scheme=1)

[**Supporting Information** Table 4.docx](https://www2.cloud.editorialmanager.com/pone/download.aspx?id=36601780&guid=4c6cb1c5-8562-456d-b03a-1ce6f3950674&scheme=1)

[Click here to access/download](https://www2.cloud.editorialmanager.com/pone/download.aspx?id=36601781&guid=11b7040b-8af7-46c8-bb29-c100105c7583&scheme=1)

[**Supporting Information** Table 5.docx](https://www2.cloud.editorialmanager.com/pone/download.aspx?id=36601781&guid=11b7040b-8af7-46c8-bb29-c100105c7583&scheme=1)

[Click here to access/download](https://www2.cloud.editorialmanager.com/pone/download.aspx?id=36601782&guid=57df7869-742c-4b4e-9269-7e20c08ebc78&scheme=1)

[**Supporting Information** Table 6.docx](https://www2.cloud.editorialmanager.com/pone/download.aspx?id=36601782&guid=57df7869-742c-4b4e-9269-7e20c08ebc78&scheme=1)

[Click here to access/download](https://www2.cloud.editorialmanager.com/pone/download.aspx?id=36601783&guid=8507955f-8aa4-4981-9833-372bdf4dd985&scheme=1)

[**Supporting Information** Table 7.docx](https://www2.cloud.editorialmanager.com/pone/download.aspx?id=36601783&guid=8507955f-8aa4-4981-9833-372bdf4dd985&scheme=1)

[Click here to access/download](https://www2.cloud.editorialmanager.com/pone/download.aspx?id=36601784&guid=c05c99d8-a41f-455b-bcdf-63ec853ff535&scheme=1)

[**Supporting Information** Table 8.docx](https://www2.cloud.editorialmanager.com/pone/download.aspx?id=36601784&guid=c05c99d8-a41f-455b-bcdf-63ec853ff535&scheme=1)

[Click here to access/download](https://www2.cloud.editorialmanager.com/pone/download.aspx?id=36601785&guid=4c423678-c83b-44ae-a701-bc86dcf2d98c&scheme=1)

[**Supporting Information** Table 9.docx](https://www2.cloud.editorialmanager.com/pone/download.aspx?id=36601785&guid=4c423678-c83b-44ae-a701-bc86dcf2d98c&scheme=1)

[Click here to access/download](https://www2.cloud.editorialmanager.com/pone/download.aspx?id=36601786&guid=4545bb65-5d35-419b-b009-4a20b183d284&scheme=1)

[**Supporting Information** Table 10.docx](https://www2.cloud.editorialmanager.com/pone/download.aspx?id=36601786&guid=4545bb65-5d35-419b-b009-4a20b183d284&scheme=1)

[Click here to access/download](https://www2.cloud.editorialmanager.com/pone/download.aspx?id=36601787&guid=bde51870-d071-4e0a-b893-29983be01901&scheme=1)

[**Supporting Information** S1_Table 1.docx](https://www2.cloud.editorialmanager.com/pone/download.aspx?id=36601787&guid=bde51870-d071-4e0a-b893-29983be01901&scheme=1)

[Click here to access/download](https://www2.cloud.editorialmanager.com/pone/download.aspx?id=36601788&guid=eca89afc-a82c-475b-b0e0-d2f7cb1fce61&scheme=1)

[**Supporting Information** S2_Table 2.docx](https://www2.cloud.editorialmanager.com/pone/download.aspx?id=36601788&guid=eca89afc-a82c-475b-b0e0-d2f7cb1fce61&scheme=1)

[Click here to access/download](https://www2.cloud.editorialmanager.com/pone/download.aspx?id=36601790&guid=7d0ff638-da25-4824-8b1c-83a9122c05b5&scheme=1)

[**Supporting Information** S4_Table 4.docx](https://www2.cloud.editorialmanager.com/pone/download.aspx?id=36601790&guid=7d0ff638-da25-4824-8b1c-83a9122c05b5&scheme=1)

[Click here to access/download](https://www2.cloud.editorialmanager.com/pone/download.aspx?id=36601791&guid=0f4d739c-6aec-44e8-8b09-ae40d260c5c9&scheme=1)

[**Supporting Information** S5_Table 5.docx](https://www2.cloud.editorialmanager.com/pone/download.aspx?id=36601791&guid=0f4d739c-6aec-44e8-8b09-ae40d260c5c9&scheme=1)

[Click here to access/download](https://www2.cloud.editorialmanager.com/pone/download.aspx?id=36601792&guid=3188975c-78ce-4dd8-a488-dedf2e58c54f&scheme=1)

[**Supporting Information** S6_Table 6.docx](https://www2.cloud.editorialmanager.com/pone/download.aspx?id=36601792&guid=3188975c-78ce-4dd8-a488-dedf2e58c54f&scheme=1)

[Click here to access/download](https://www2.cloud.editorialmanager.com/pone/download.aspx?id=36601793&guid=0d979222-0432-4537-9777-391f43811df6&scheme=1)

[**Supporting Information** S7_Table 7.docx](https://www2.cloud.editorialmanager.com/pone/download.aspx?id=36601793&guid=0d979222-0432-4537-9777-391f43811df6&scheme=1)

[Click here to access/download](https://www2.cloud.editorialmanager.com/pone/download.aspx?id=36601794&guid=a80b5d48-7a09-4d33-aa9a-2037d6426793&scheme=1)

[**Supporting Information** S8_Table 8.docx](https://www2.cloud.editorialmanager.com/pone/download.aspx?id=36601794&guid=a80b5d48-7a09-4d33-aa9a-2037d6426793&scheme=1)

[Click here to access/download](https://www2.cloud.editorialmanager.com/pone/download.aspx?id=36601795&guid=45763949-7029-4c30-8e4e-9f6a33f20aa7&scheme=1)

[**Supporting Information** S9_Table 9.docx](https://www2.cloud.editorialmanager.com/pone/download.aspx?id=36601795&guid=45763949-7029-4c30-8e4e-9f6a33f20aa7&scheme=1)

[Click here to access/download](https://www2.cloud.editorialmanager.com/pone/download.aspx?id=36601796&guid=0e56181b-2a84-4db4-9a3a-a874d4adc10d&scheme=1)

[**Supporting Information** S10_Table 10.docx](https://www2.cloud.editorialmanager.com/pone/download.aspx?id=36601796&guid=0e56181b-2a84-4db4-9a3a-a874d4adc10d&scheme=1)

Supporting Information

[Click here to access/download](https://www2.cloud.editorialmanager.com/pone/download.aspx?id=36601797&guid=83ad14d2-7c86-42c6-b1e5-e2bddee955b2&scheme=1)

[**Supporting Information**](https://www2.cloud.editorialmanager.com/pone/download.aspx?id=36601797&guid=83ad14d2-7c86-42c6-b1e5-e2bddee955b2&scheme=1)

[Non-author contact information.docx](https://www2.cloud.editorialmanager.com/pone/download.aspx?id=36601797&guid=83ad14d2-7c86-42c6-b1e5-e2bddee955b2&scheme=1)

[Click here to access/download](https://www2.cloud.editorialmanager.com/pone/download.aspx?id=36601789&guid=dcb8fd95-57ca-4c0c-b833-5ad3215043d7&scheme=1)

[**Supporting Information** S3_Table 3.docx](https://www2.cloud.editorialmanager.com/pone/download.aspx?id=36601789&guid=dcb8fd95-57ca-4c0c-b833-5ad3215043d7&scheme=1)

Revised Manuscript with Track Changes

## 1 Impact of Urban Productive Safety Net Program on Urban

2 Households’ Asset Accumulation and Food Consumption

## 3 Rate in Dessie City, South Wollo Zone, Amhara Region,

| 4 | Ethiopia |  | | |
| --- | --- | --- | --- | --- |
| 5 | Alem-meta Assefa |  |  |  |
| 6 | Department of Geography and Environmental Studies, Wollo University, | P.O. | Box | 1145, |
| 7 | Dessie, Ethiopia |  |  |  |
| 8 |  |  |  |  |
| 9 |  |  |  |  |
| 10 | Corresponding author’s email: [alemmeta99@yahoo.com](mailto:alemmeta99@yahoo.com) |  |  |  |
| 11 | Wollo University, Department of Geography and Environmental Studies, | P.O. | Box | 1145, |
| 12 | Dessie, Ethiopia |  | | |
| 13 | Corresponding author’s ORCID: [https://orcid.org/0000-0002-1856-](https://orcid.org/0000-0002-1856-2373) |  |  |  |
| 14 | [2373](https://orcid.org/0000-0002-1856-2373) |  |  |  |
| 15 |  |  |  |  |
| 16 |  |  |  |  |
| 17 |  |  |  |  |
| 18 |  |  |  |  |
| 19 |  |  |  |  |
| 20 |  |  |  |  |
| 21 |  |  |  |  |

##### 22 Abstract

23 Agriculture is the driver of economic growth in many developing countries like Ethiopia, where
24 it often represents at least 25% of gross domestic product. But the quality and productivity of
25 the land depends on the way agriculture is practiced. Better agricultural techniques are
26 necessarily including Productive Safety net Program. Urban Productive Safety Net Program is
27 designed to support the poor households and establish urban safety net mechanisms. While
28 several studies have examined the effects of rural productive safety net programs, the outcomes
29 of similar programs in urban areas are not well documented. This knowledge gap hinders the
30 ability to effectively design and implement urban productive safety net programs. The main
31 objective of this study was to assess the impact of urban productive safety net program on asset
32 accumulation and consumption rate in Dessie city. Both primary and secondary data sources
33 were used. A total of 112 households were selected randomly among the public work Urban
34 Productive Safety Net Program beneficiary and non-beneficiary households. Descriptive
35 analysis, inferential statistics and econometric models were used for analysis. Based on the
36 econometric estimation results the demographic and socio-economic variables such as
37 education, access to credit, access to extension services and adult male labor force revealed
38 positive relation to public work urban productive safety net program beneficiary and non-
39 beneficiary household’s asset accumulation and consumption. The impact of program
40 participation was also found to be positive and significant for both home asset and community
41 asset. While food and non-food consumption even the total consumption and food security
42 status are also positive. Generally, the result indicated that, due to program participation
43 beneficiary households have higher home asset and community asset and better consumption
44 and food security status.
45 **Keywords:** Productive safety net program; asset accumulation; consumption; Dessie City

# 46 1. Introduction

47 In Sub-Saharan Africa, numerous individuals continue to experience chronic food insecurity
48 caused by repeated droughts, limited productivity in subsistence agriculture, and widespread
49 poverty [1]. Samuel [2] states that various food security initiatives have been created to address
50 this ongoing issue effectively. Accordingly, in the past two decades we have seen a rapid
51 increase of social protection programs in African countries to alleviate poverty, food insecurity,
52 and vulnerability of poor households [3]. These formal programs aim to assist individuals and
53 households facing chronic food insecurity or temporary financial setbacks by offering income
54 support or alternatives, such as cash and in-kind transfers, subsidies, and labor-intensive public
55 work. The Productive Safety Net Program (PSNP) is a large-scale social protection program
56 implemented in Ethiopia since 2005. It aims to provide assistance to chronically food-insecure
57 households and help them improve their food security and resilience to shocks. The program
58 combines two main components: direct support through cash and/or food transfers to eligible
59 households, and support for public works programs. The cash and food transfers are intended
60 to meet immediate consumption needs, while public works programs provide temporary
61 employment opportunities and help build community assets such as roads, bridges, and
62 irrigation systems.
63 The relative lack of change in hunger at the global level from 2021 to 2022 hides substantial
64 differences at the regional level. Progress was made towards reducing hunger in most sub
65 regions in Asia and in Latin America, but hunger is still on the rise in Western Asia, the
66 Caribbean and all sub regions of Africa. The proportion of the population facing hunger is
67 much larger in Africa compared with the other regions of the World – nearly 20 percent
68 compared with 8.5 percent in Asia, 6.5 percent in Latin America and the Caribbean, and 7.0
69 percent in Oceania [4].
70 An estimated 149 million Africans are facing acute food insecurity—an increase of 12
71 million people from a year ago [5]. This equates to a risk category of 3 or higher (Crisis,
72 Emergency, and Catastrophe) on the Integrated Food Security Phase Classification (IPC) scale
73 of 1 to 5. Some 122 million of those facing acute food insecurity are in countries experiencing
74 conflict—82 percent of the total—accentuating that conflict is the primary driver of acute food
75 insecurity in Africa [6]. Four of the top 10 countries facing the most acute food insecurity are
76 in East Africa—Sudan, Ethiopia, South Sudan, and Somalia. Ethiopia, in particular, had an
77 estimated 20.1 million people who are facing food insecurity, and 15.1 million require
78 emergency food assistance, following a 2-year war in Tigray, conflicts in other parts of the
79 country, as well as a severe drought that struck the Horn of Africa [6]. These individuals lacked
80 consistent access to sufficient food, leading to asset selling and consumption restrictions in
81 order to survive [7].
82 Before implementing PSNP, the Ethiopian government relied on unpredictable annual appeals
83 for emergency food relief, which saved lives but failed to address the increasing number of
84 food insecure individuals [7]. Based on the information provided, the study conducted by
85 Berhane et al. [8] indicates that the second phase of the Productive Safety Net Program (PSNP)
86 in Ethiopia has several successes when compared with the initial emergency relief appeal
87 method. These successes include:
88 **Enhanced food security**: The study suggests that the PSNP plays a crucial role in ensuring
89 food security for both chronically and temporarily food insecure households. By providing
90 program transfers and supporting productive activities, the PSNP aims to assist over 7.5 million
91 people in 318 districts across eight regions. This targeted approach is likely more effective in
92 addressing the needs of food insecure individuals compared to the unpredictable annual appeals
93 for emergency food relief [9].
94 **Long-term planning and stability:** Unlike the initial emergency relief appeal method, which
95 relied on unpredictable annual appeals for food aid, the PSNP operates on a set timeline until
96 December 2014. This longer-term planning allows for more stability and predictability in
97 addressing food insecurity issues [10].
98 **Diversification of support:** The PSNP not only provides program transfers but also supports
99 productive activities. This indicates that the program aims to go beyond providing immediate
100 relief by helping households develop sustainable livelihoods and improve their resilience to
101 food insecurity.
102 However, without further information on the specific failures of the initial emergency relief
103 appeal method, it is challenging to make a direct comparison. It is important to note that the
104 PSNP is a complex program with its own limitations and challenges. Further analysis and
105 evaluation would be needed to understand the full extent of its successes and failures in
106 comparison to the initial emergency relief appeal method.
107 In an attempt to break the cycle of annual appeal of food aid and achieve an acceptable level
108 of food security at macro (national) and micro (household) level, the government of Ethiopia
109 developed a food security strategy (FSS) in November 1996 [11]. The FSS highlighted
110 government plans to address the causes and effects of food insecurity in Ethiopia. Based on the
111 FSS, the government designed regional food security programs and projects in 2002 [12].
112 The PSNP is currently the largest operating social protection program in Sub-Saharan Africa,
113 outside of South Africa. It differs from previous food-for-work programs, in that it focuses
114 continuously on selected households over several years and in that the explicit objective was
115 that it will eventually be phased out [13].
116 The Ethiopian government has been working on a program called the Urban Productive Safety
117 Net Program (UPSNP) that will span a 10-year period. This program is part of the Urban Food
118 Security and Job Creation Strategy, which was officially endorsed on May 8, 2015. The
119 purpose of the UPSNP is to provide assistance to more than 4.7 million impoverished
120 individuals living in 972 cities and towns across the country. It is envisaged that this will be
121 achieved over a long-term period through a gradual roll-out plan of different phases starting
122 with big cities having a population of over 100,000 people. The proposed Bank support will
123 provide assistance for the first five-year phase of the government program and is targeting 11
124 major cities, Addis Ababa and one city from each region (Adama, Assayita, Asosa, Dessie,
125 Dire Dawa, Gambella, Hawassa, Harari, Jijiga, and Mekele). In the initial stage of the UPSNP,
126 approximately 604,000 individuals who are among the most impoverished 12 percent and around
127 55 percent of the population residing below the poverty threshold in these 11 cities were the
128 recipients of the program's benefits [14].
129 Dessie City was one of chronically food insecure cities in Ethiopia. The first phase of the
130 program started in 2017 through direct support and public work by choosing beneficiaries from
131 selected *Kebeles* [15]. Despite the introduction of multiple food security interventions with
132 PSNP, Dessie City has been classified as a typical food insecure area. Having these in mind,
133 the researchers tried to evaluate the impacts of urban productive safety net program on urban
134 households’ asset accumulation, consumption and food security status in Dessie City.
135 Accordingly, the conceptual framework shows several factors; like personal, socio-economic,
136 institutional and bio-physical factors that affect household participation in UPSNP and its
137 impact (Fig 1).

##### 138 Fig. 1 Conceptual Framework of the Study

139 Historically, urban poverty has consistently faced the significant challenge of food insecurity,
140 especially in drought-prone areas of Ethiopia [16]. The responses to food security have
141 conventionally been dominated by emergency food-based intervention. High proportion of
142 food insecure households have been receiving emergency food aids. Although the emergency
143 assistance was substantial and saved many lives, there was uncertainty of the food aid, poor
144 timing of provision and insufficient quantity of aid for the individual’s beneficiary was among
145 the main defects of food aids in the past and creating dependency syndrome on the recipients
146 [17]. For that reason, the past decades of large-scale food aids deliveries have done little to
147 prevent households’ asset depletion, prevent environmental degradation and chronically food
148 insecurity in general, because of ignorance of integrating these aids with natural resource
149 management in the country.
150 Recognizing this, the government of Ethiopia changed the emergency food-based assistance
151 to multi-year support of PSNP in 2005 that provides cash and food transfers to chronically food
152 insecure households with the aim of preventing asset depletion at household level while
153 creating assets at the community level [18]. At national level, there were some works that tried
154 to highlights the impacts of program intervention, particularly PSNP. Unfortunately, most of
155 them have focused on the impacts of PSNP on asset building and consumption in rural areas
156 rather than urban areas. According to Kataru’s [19] research finding, it has been determined
157 that the Productive Safety Net Program (PSNP) is capable of effectively mitigating
158 consumption fluctuations and safeguarding household assets. Berhane et al. [20] indicated that
159 beneficiary households have managed to significantly improve their food security status by
160 1.05 months. On the contrary, what has been demonstrated is that there is no noteworthy
161 distinction between the consumption and assets of individuals involved in the PSNP program and
162 those who are not [21]. Similarly, Andersson et al. [22] indicated that there was no increment
163 in livestock holdings for program participants. Furthermore, Habitamu [23], stated that,
164 participation in PSNP reduces the asset size of the participants, due to the dependency behavior
165 of beneficiaries which discourage them to put efforts on their agricultural activities. But, none
166 of them discussed the magnitude of the impact of PSNP on asset accumulation and
167 consumption on urban households.
168 From the above statements, we understand that various researchers have examined the effects
169 of the program at both the national and local levels, but their findings have been inconsistent and
170 far from conclusive. Moreover, while several studies have examined the effects of rural
171 productive safety net programs, the outcomes of similar programs in urban areas are not well
172 documented. This knowledge gap hinders the ability to effectively design and implement urban
173 social protection interventions that can enhance the economic well-being and food security of
174 vulnerable urban populations. Consequently, this study was conducted to address these
175 knowledge gaps.

# 176 2. Materials and Methods

### 177 2.1 Description of the Study Area

178 Dessie City is aged more than a hundred and ten years old and also capital of Dessie Zuria
179 *Wereda* and South administrative Wollo Zone. It is located between 11^0^08’North Latitude and
180 39^0^38’East Longitude (Fig 2). It is also situated in the north eastern part of the country at 401km
181 away from Addis Ababa, the capital of Ethiopia. The city is placed at the foot of Tossa Ridge
182 and is surrounded by and also incorporates a number of hills within its boundary. The average
183 elevation of the city is 2,600 m.a.s.l. Dessie was established in 1893 by King Michael. The city
184 serves as social, economic and political center for the South Wollo Zone and their
185 administration. According to the Central Statistics Agency [24], the total population of the city
186 is approximately 250,346 and its annual growth rate is 3.72%. The sex composition of the
187 population is 46.8% male and 53.2% female. The city has 5 sub-cities and its total area is

188	16,745.82 hectares.

189 **Fig. 2 Map of the Study Area**

### 190 2.2 The Research Design and Sampling

191 The research was conducted through descriptive survey design. The survey design helps to
192 identify patterns of behavior, activities, people’s attitude, opinions, habits and any of the variety
193 of social problems that influence the explicability and scaling-up efforts of the program across
194 various contexts [25]. The utilization of a descriptive survey design aids in identifying past
195 occurrences and enables the pre-determination of specific preventive measures that can be
196 implemented.
197 The study used mixed research approach. Accordingly, both quantitative and qualitative data
198 were collected and analyzed at the same time. Data obtained through interview, questionnaire
199 and observation were analyzed qualitatively. The quantitative data were analyzed and
200 interpreted through appropriate statistical tools and cross tabulations were made to facilitate
201 meaningful analysis and interpretations of the research findings.
202 **Selection of Sample Site:** On the bases of the researchers’ prior knowledge of the area and
203 work place Dawudo Sub-city was selected purposively among the 5 sub-cities of Dessie City.
204 Then, in Dawudo sub-city, Robit and Bahil Amba *Kebeles* were selected by simple random
205 sampling method.

##### 206 Selection of Sample Households

207 Creswell [26], stated that all target populations are member of a real set of subjects to which a
208 researcher wishes to generalize the results of the study. The study specifically focused on each
209 chosen Kebele, and the researcher obtained the complete roster of household heads from the
210 Kebele administration office.
211 Then on the bases of *Kebele* document there are 338 Public Work-Productive Safety Net
212 Program (PW-PSNP) beneficiary households and 272 eligible non-beneficiary households in
213 Robit *Kebele*. While 337 PW-PSNP beneficiary households and 273 eligible non-beneficiary
214 households in Bahil Amba *Kebele,* thus totally 1220 both in PW-UPSNP beneficiary and
215 eligible non-beneficiary household heads were used as sample frames included for the survey.
216 Then after, the researcher used the known formula so as to minimize the number of household
217 heads in the study as a sample. Thus, Kothari [27] sample size determination was employed as
218 shown below:
219 219

n =	z^2^.p.q.N

e^2^(N−1)+Z^2^.p.q

220 Where: n = Sample size
221 z =Standard variation at 95 percent confidence interval (1.96)
222 p = Sample proportion in the target population estimated to have the characteristics being
223 measured (0.03)

224	q = 1-p

225 N =size of the target population is 1220
226 e= the estimate should be within 3 percent of the true value (0.03)
227 227

Therefore, the desired sample is n =	^1.96^2.0.03.(1−0.03).(1220)	= 112.4

0.03^2^(1219 −1)+(1.96)^2^.0.03.(1−0.03)

228 228

n=	0.03(1.96^2^ x0.97x1220)	=

0.03(0.03 𝑥 1219+1.96^2^)

3.8416𝑥0.97𝑥1220	= n=

36.57+3.8416

4546.14944	=112

40.4116

229	n =**112**

230 Then by using the above formula, 112 household heads were selected as a representative
231 sample. These household heads were selected based on proportional random sampling
232 technique or ratio to the total household heads (Table 1). In addition to this, interviews were
233 conducted with six key informants, specifically selected *Kebele* Development Agents, *Kebele*
234 Administrators, and the head of the UPSNP Task Force members. Finally, both the sample
235 households and the key informants were orally asked to express their agreement for data
236 collection interview. As a result, they expressed their consent verbally.

### 237 2.3 Data Sources and Collection Techniques

238 The study employed both primary and secondary data sources. The primary data were collected
239 from public work UPSNP beneficiary and eligible non-beneficiary household heads through
240 questionnaires as well as key informant interview. While secondary data were collected from
241 different published and unpublished sources of data. Data collection techniques of the study
242 include questionnaires, key informant interview, and field observation.

### 243 2.4 Methods of Data Analysis

244 The study employed both quantitative and qualitative data analysis techniques. Quantitative
245 data were gathered through the close-ended questionnaire and analyzed by using descriptive
246 statistics, inferential statistics and econometric tools using stata computer software. Similarly,
247 qualitative data was gathered through open-ended questionnaires and interview and analyzed
248 textually to supplement the questionnaire survey.
249 To assess the distribution of the covariates between the two groups, the quantitative data
250 underwent analysis using descriptive techniques. This involves examining the independent
251 variables through methods such as calculating frequencies and percentages, which were then
252 summarized in tables to interpret and draw conclusions from the results. Inferential statistical
253 tools, including mean, Chi-square test, one-sample t-test, and paired t-test, were employed to
254 determine the significance of mean values for continuous variables between the households of
255 PW-PSNP beneficiaries and eligible non-beneficiaries. To estimate the impact of household’s
256 participation in the Urban Productive Safety Net program on asset accumulation and
257 consumption expenditure logit model and an econometric model (Propensity score matching
258 method) were used in addition to the simple descriptive analysis.

##### 259 2.4.1 Binary Logistic Regression Model

260 According to Gujarati [28], in estimating logit model for the dichotomous (binary) logistic
261 regressions was used to achieve this objective, particularly; participation which takes a value
262 of 1 if the household participated in a program and 0 otherwise. The logit model was selected
263 for this study because the dependent variable is dichotomous in nature and the computation
264 will be easier. For the binary treatment case, where we estimate the probability of participants
265 versus non-participants of UPSNP is addressed as a decision involving binary/dichotomous
266 response variable. The logit model is mathematically formulated as follow:
267 267

Pi =

𝑒𝑍𝑖

zi	**(1)**

1+𝑒

268 Where, Pi -is the probability of participation in the PSNP in relation to explanatory variable
269 ezi- irrational number to the power of Zi
270 Zi = βo + Σ Βi Xi + Ui	**(2)**
271 Zi - a function of n explanatory variable 1, 2…	n )
272 βo - is the intercept
273 Βi- is regression coefficients to be estimated
274 Xi- is pre-intervention characteristics (explanatory variable)
275 Ui- is a disturbance term
276 The probability that the household belongs to non-participant group is given by:
277 277

1-Pi =

1

1+𝑒

zi	**(3)**

278 Then the odd ratio was written as:
279 279

Pi 1−Pi =

1+𝑒𝑧𝑖

1+𝑒

_zi = 𝑒

𝑧𝑖

**------------------------------------------------------------------------------(4)**

Pi

280 . It is simply the odds ratio in favor of participating in the UPSNP. It is the ratio of the

1−𝐏𝐢

281 probability that the household would participate in the UPSNP to the probability that he/she
282 would not participate in the UPSNP.
283 By taking the natural log of equation (4), the log of odds ratio was written as:
284 284

Li = Ln ( ^Pi^

1−𝐏𝐢

) = Ln ( 𝑒^𝑧𝑖^ ) = Zi = βo + Σ ΒiXi + Ui	(**5**)

285 Li - is the log of the odds ratio in favor of participation in UPSNP which is linear both in Xi
286 and parameters.
287 **2.4.2 Propensity Score Matching (PSM) Method**
288 As placement to participate in PSNP is not random, i.e., an eligible household is deliberately
289 selected based on asset size, vulnerability to shocks and food insecurity.	In addition, the
290 baseline survey was not conducted prior to the intervention of the PSNP in the study area.
291 Therefore, propensity score matching method is usually used to assess the impact of a program,
292 in this case, we used to address the impacts of PSNP on asset accumulation and consumption
293 in the beneficiary households.
294 According to Gilligan et al. [29] this model assumes that, “after controlling for all pre-
295 intervention observable household’s and community’s characteristics that are correlated with
296 both program participation and the outcome variable, untreated groups have the same average
297 outcome as the treated group would have had they did not receive the program”. This is the
298 basic idea, to found in group of non-participants those individuals who are similar to the
299 participants in all relevant pre-treatment characteristics X. Hence, the matching approach is
300 one of the best solutions.
301 **Procedures to estimate propensity score:** It is the first step of PSM technique. Rosenbaum
302 and Rubin [30] revealed that matching can be performed conditioning only on P(X) rather than
303 on X, where P(X) = Prob (D=1|X) is the probability of participating in the program conditional
304 on X. According to these authors, if outcomes without the intervention are independent of
305 participation given X, then they are also independent of participation given P(X). Estimating
306 the propensity score involves decision on three choices: what model to be used for the
307 estimation and what variables should be included in this model and matching algorism.

##### 308 Impact Indicator Variables for Asset Accumulation and consumption

309 The impact of the PSNP is analyzed by dividing the two categories which are the asset
310 building and consumption expenditure of the PW-PSNP beneficiary and eligible non-
311 beneficiary households [31].
312 **Outcome Variables for Asset Accumulation:** The first is the home asset (which is the sum
313 of household goods and consumer durables) and community asset value is also measured
314 based on the estimation of its market value by respondents in terms of Birr [32].
315 **Outcome variables for consumption**: In order to analyze the impacts of the PSNP on the
316 household’s consumption the study has attempted to examine it’s by classifying the
317 households’ consumption into two main categories, because households have consumed
318 different items and due to this reason their value might not the same. Therefore, in the study,
319 the households have been asked about the quantity as well as the current price of overall
320 households mean real food consumption expenditure per Adult Equivalent (AE) in terms of 24
321 hours and current non-food consumption expenditure per adult equivalent (AE) in terms of
322 year, at their disposal, during the field survey based on the respondents’ consumption rate in
323 terms of Ethiopian Birr [33].
324 324
325 325

# 326 3. Results and Discussions

### 327 3.1 Descriptive Analysis of the Dummy Variables Used in Logit

328 **Model**
329 **Sex of the household head:** In the urban area, female headed households are more effective
330 in feeding their family in a sustainable manner from year to year which makes them more likely
331 to be food secure. Because, female headed households are in a better labor input than the males
332 headed in urban area. Based on the respondents result in this study, the sample was composed
333 of both male and female headed households from the total sample households 94 (84%) and 18
334 (16%) were male and female headed household respectively. From the total male headed
335 household respondents, that have been included in the study, 54 (48%) are from the PW-PSNP
336 participant group while the other 40 (36%) are from the non-PSNP participants. In other way
337 female headed households represent about 8 (7%) and 10 (10%) from PW-PSNP participant
338 and non-PSNP beneficiary households respectively. As the result indicates that, in the study
339 area the male headed households were a higher chance to PSNP participants in the public work
340 components of the PSNP. Therefore, as compared to their female headed households’
341 counterpart male headed households participate more in the PSNP.
342 **Credit Experience**: It is indicating whether the household had credit experience or not. Those
343 who had credit experience can use it to purchase something so that they would manage to
344 escape the liquidity constraints experienced by many urban households in the study area.
345 Moreover, the credit could also serve as a capital source to fund any off-farm activity that the
346 households may undertake indicating that credit experience minimizes the danger of being food
347 unsecured. Amhara Credit and Saving Institution is one of the main and dominant credit
348 suppliers in the study area. Prior to PSNP of the respondents only 11 (10%) PW-PSNP
349 participant and 7 (6%) of non-PSNP participants had taken a loan. Prior to PSNP shows that
350 about 51 (46%) of respondents and 43 (38%) of non-beneficiary respondents had not borrowing
351 credit experience. Access to credit after the implementation of the program indicated that out
352 of the total respondents 38 (34%) of them were public work of PSNP beneficiaries and 33
353 (29%) non-beneficiaries households received credit, mainly to purchase food item, and
354 improve household asset. It indicates that, higher percent of PSNP public work participants had
355 access to credit compared to non-participant groups. Hence, after the program, from the total
356 respondents of public work PSNP beneficiary have been better and borrowing experience than
357 non-beneficiary household. It indicates that greater percentage of beneficiary households
358 mainly to purchase food item and improve home asset compared to non-beneficiary
359 households. Moreover, the survey result revealed that out of the total respondents with off-
360 farm income, 51 (46%) of them were from the participant group and the 41 (37%) were non-
361 participant households. A study done by Derso et al. [34] supported this result. It indicated that
362 three in four households were experiencing food insecurity and that an appropriate multi-
363 dimensional approach such as the opening of small business enterprises through credit service
364 support may help to diversify income sources and to reduce the dependency ratio. While,
365 without off-farm income, 11 (10%) of them were from the participant group and the others 9
366 (8%) were found to be non-participant households; regarding access to extension service about
367 55 (49%) of PSNP in public work participants and 33 (29%) of non-participants were receiving
368 the type of training in 2019/20. But, the remaining 7 (6%) of PSNP participants and 17 (15%)
369 non-participants did not receive any type of training. Based on these results, to some extent the
370 extension service was highly supportive to the PSNP beneficiary households when compared
371 with non-beneficiary households. According to the survey result the educational status of the
372 household revealed that 31 (27.6%) of PW-PSNP beneficiary households were literate (able to
373 read and write) and 25 (22%) of non-beneficiary households were literate (able to read and
374 write). While, 31 (27.6%) and 25 (22%) of PW-PSNP beneficiary and PSNP non-beneficiary
375 household heads were illiterate (unable to read and write) respectively. The result indicated
376 that, both illiterate and literate household heads in the respondent were similar in proportion
377 (Table 2).
378 On the other hand, access to remittance the result of the study showed that about 51(45.5%)
379 of the PW-PSNP beneficiary households did not receive any kind of remittance, and the rest of
380 11 (10%) received remittance mainly to purchase food item and none food items. while from
381 the total none beneficiary households, 11 (10%) household heads who took remittances and the
382 remaining 39 (35%) non-beneficiary household heads did not received remittance respectively.
383 This means greater percentages of beneficiary of the UPSNP are not received remittance as
384 compared to non-beneficiary. Besides, the result of the study also revealed that food gap faced
385 households prior to the introduction of the program three month and above were faced food
386 gap, 58 (52%) and 48 (43%) of PW-PSNP beneficiary and non-PSNP beneficiary households’
387 groups had food gap on three months of experienced food gaps respectively. It implies that
388 prior to the introduction of the program higher percentage of participant groups were faced
389 food gap and food insecure compared to non-beneficiary households (Table 2 above).

### 390 3.2 Descriptive Analysis of the Continuous Variables Used in Logit

391 **Model**
392 **Age of the household head:** It is a continuous variable that refers to the level of `experience
393 the farmers possess and measured in the number of years. The older the head of the household
394 is the more he/she would be effective in risk diversification as well as in predicting the weather
395 and applying the right technology. However, the existence of large number of children under
396 the age of 15 and old age of 65 and above in the family affect the household consumption
397 negatively. The households with higher dependency ratio will have lowest welfare status. This
398 means households with higher dependency ration had low level of welfare, hence higher
399 probability to fall in to food in security and poverty. The result of the study indicated that the
400 average age of the household head for the whole sample is around 49.17 years (Table 3). When
401 we compare the two groups in terms of their mean age, it was found that treated groups are
402 older than their controlled groups by about 1.58 year, i.e., 49.91 years compared to 48.33 years
403 respectively. Thus, the older household heads were found to be food in secured.
404 **Dependency Ration:** The dependency ratio shows the ratio of economically active persons
405 compared to economically dependent household members. Economically active members of
406 households, whose age is from 14 to 64 were assumed to be the principal productive force and
407 sources of income for the household. Household members whose age was between 0-14 and
408 above 64 were considered as economically inactive and dependent members of the household.
409 Similarly, the dependency ratio for the members of the sampled households estimated to be
410 1.25 and 1.08 of the PW-UPSNP beneficiaries and non-UPSNP beneficiaries respectively.
411 Whereas, the average difference between treated and non-treated group are around 0.32.
412 Therefore, the result indicates that treated groups are the higher dependency than control
413 groups. Thus, the higher dependency contributes to food insecurity and the probability level of
414 participation significantly 1 percent (Table 3 above).
415 **Household Family Size:** It refers to the total number of the household’s member. Family size
416 is one of the major socioeconomic variables, which as a significant influence on the amount of
417 household consumption. As the number of the family increases, given the fixed resources, the
418 food share goes to the individual in that family declines which maximizes the probability of
419 being food insecure. As presented in Table 3, the average family size of the combined samples
420 were around 4. The average family size of beneficiary and non-beneficiary households was 4
421 and 3 respectively. As a result, beneficiary households had greater family size compared to
422 non-beneficiary households (Table 3 above).

### 423 3.3 Impact of Productive Safety net Program on Community and

424 **Home asset values**
425 Different types of sustainable productive activities were being undertaken through public
426 works component of PSNP to build community assets. These activities largely focused on the
427 households’ area of community asset like maintenance of roads, maintenance and construction
428 of schools, maintenance and construction of latrine and cleaning surrounding environment.
429 Whereas, home asset (consumer and durable assets like radio, wrist watch, bed, mobile phone,
430 Tape recorder, Television and housing condition) were included. Then, the result of the study
431 revealed that in the households’ area of community asset and home asset value calculated as
432 the beneficiary worked and contribute as estimated at the values have 345.6 and 711.6
433 Ethiopian Birr respectively. Whereas, non-beneficiary households of community asset and
434 home asset value contribute to have 291.1 and 254.6 Ethiopian Birr respectively. Therefore,
435 the beneficiary households had greater amount of community and home asset building
436 tendency than non-beneficiary households (Table 4). According to this finding, the mean
437 difference in community asset and home asset value contribute between the beneficiary and
438 non-beneficiary households were found to be positive and statistically significant at 1% of
439 probability level. This implies that, the PSNP beneficiary households possess community asset
440 and home asset value that have contribute to greater value (quality) as compared to the assets
441 possessed by the non-PSNP beneficiary households.

### 442 3.4 Impact of Productive Safety Net Program on Consumption

443 Consumption expenditure was used as impact indicator while evaluating impact of the UPSNP,
444 and it was computed as per adult equivalent consumption expenditure. The overall households
445 mean real consumption expenditure per adult equivalent (AE) for the sample households was
446 257.63 Ethiopian birr. The mean consumption expenditure for PSNP beneficiaries and non-
447 beneficiary groups was 295.21 and 215.54 Ethiopian Birr respectively. The mean difference
448 test of consumption expenditure for the two groups was 79.68 Ethiopian Birr. It implies that
449 statistically significant at 1% probability level. Whereas the overall households mean real food
450 consumption and non-food consumption expenditure per adult equivalent (AE) for the sample
451 households was 209.95 and 47.68 Ethiopian Birr respectively (Table 5). The mean food
452 consumption expenditure for PSNP beneficiary and non-beneficiary groups was also 237.72
453 and 178.86 Ethiopian Birr respectively. The mean difference test of consumption expenditure
454 for the two groups was 58.86 Ethiopian Birr. It implies that statistically significant at 1%
455 probability level. Moreover, the mean non-food consumption expenditure for PSNP
456 beneficiary and non-beneficiary groups were 57.49 and 36.68 Ethiopian Birr respectively.
457 Therefore, the result of the study showed that the PSNP beneficiary households were found to
458 be positive and statistically significant at 1% over the intervention period. Besides, the overall
459 consumption per adult equivalent for the participants’ increased when compared to non-
460 participants over the intervention period. This result was supported by the evidences that taken
461 from the key informants at the time of interview. One of the key informants said that:
462 *“The beneficiary households have better money to pay for food items, medical cost and school fee with the help*
463 *of PW-UPSNP. Before they included in PSNP, some of the beneficiary households were eating once a day but*
464 *some others were eating twice in a day. After they were included in the PW-UPSNP, the majority of the beneficiary*
465 *households are eating three times in a day”.*
466 466

### 467 3.5 Econometric Results

468 The study used propensity score matching to evaluate the effects of PSNP on asset
469 accumulation and consumption expenditure among urban households. The propensity score
470 was estimated using a logistic model based on observable characteristics to determine the
471 likelihood of households participating in the program. Beneficiary and non-beneficiary
472 households were then matched based on their propensity scores, using a binary outcome
473 variable (1 for PW-PSNP beneficiary households and 0 for eligible non-PSNP beneficiary
474 households) in the logit model.

##### 475 3.5.1 Factors of Participation in Productive Safety Net Program

476 The researchers have incorporated relevant variables to explain the dependent variable and
477 conducted a logit model. They found that the gender, age of the household head, family size,
478 and off-farm income activities significantly affected program placement during selection.
479 The findings show that various factors significantly affected participation in PW-PSNP.
480 Employment on wage, family size, number of adult females, age of household head, and
481 employment on own business before UPSNP intervention all had positive and significant
482 influences on PW-PSNP participation, with varying magnitudes of impact.
483 Households with larger family sizes are more likely to participate in the program compared to
484 those with smaller family sizes. This is because larger families often have higher food demand
485 and a higher chance of food insecurity due to limited resources. The study found family size to
486 be statistically significant at a 5% level of significance. This result is consistent with the studies
487 conducted in Kenya and other rural parts of north Ethiopia by [35, 36] found that households
488 with a large family size were more like to be food secure as compared to those with a small
489 family size. The reason may be as the household size becomes larger, the number of
490 economically active members may also increase, which leads to increase their participation in
491 various income-generating activities that could boost their food security than for households
492 with lower family size. Additionally, male-headed households were more likely to be program
493 participants compared to female-headed households, likely due to females being active in non-
494 farm activities generating off-farm income. This result was also statistically significant at a 5%
495 level of significance.
496 Household head age is the other variable which is found to be negatively significant at 1%
497 level of significant and related with probability of participation in PW-PSNP. This implies that
498 as increase in age of household heads, decreases their participation in the program. This is due
499 to the reason that, the age goes beyond certain periods; the person may become physically weak
500 and food insecure. On the other hand, variables like sex of household head, food gap, accesses
501 to extension services and access to remittance and number of adult males are found to be
502 statistically insignificant (Table 6).
503 **3.5.2 Impact of UPSNP on Household Asset Accumulation**
504 In this study, household home assets represent the sum of household goods like home, pot,
505 oven, bed, box and barrel and consumer durables like mobile phone, watch, tape recorder and
506 Radio and Television etc. After converting both the household goods and the consumer durable
507 of the entire households in to their respective market value, the two categories have been added
508 together to form the household home assets.
509 The impact of UPSNP on household’s home asset were found to be positive with the
510 maximum and the minimum ATT value calculated to be 494 Ethiopian Birr (based on NN
511 matching) and 441 Ethiopian Birr (based on Kernel matching) (Table 7) respectively.
512 Statistically, this result is found to be significant at one percent for all matching estimators used
513 in this study, i.e., based on Kernel, Stratification and NN matching. The implication of this
514 result is, because of the introduction of the program, the value of non-productive/home asset at
515 the hands of the PW-PSNP beneficiary households is higher than the value of the non-
516 productive asset at the hands of the non-beneficiary households by the amount of at maximum
517 494 Ethiopian Birr and at minimum 441 Ethiopian Birr. And this result confirms the fact that
518 the program is successful in avoiding the depletion in the non-productive asset of the
519 beneficiary households.
520 According to this finding, the program has a positive and significant impact on the household
521 asset at 1% level of significance. This can be also stated as PW-PSNP beneficiary households
522 have more asset value than the eligible non-beneficiary households. Generally, the above
523 positive value of ATT implies that, because of the program intervention household’s home
524 assets have been protected in the study area.
525 During the field survey, the participants of the PW-PSNP program made efforts to enhance
526 community assets, as evidenced by their contributions to activities such as road maintenance,
527 improving the greenery of the environment, and erecting fences around schools, health posts,
528 and ponds. The result of this study shows that the current value of community assets at the
529 hands of the PSNP beneficiary households is higher than the current value of community assets
530 at the hand of the non-PSNP beneficiary households.
531 The mean difference in the current value of community assets between the PW-PSNP
532 beneficiary and eligible non-beneficiary households were found to be positive and significant
533 with the minimum and maximum ATT value of contribute as calculated 54 Ethiopian Birr
534 (based on Stratification matching) and 82 Ethiopian Birr (based on the NN matching)
535 respectively. Statistically, the calculated ATT for the treated and control group is found to be
536 significant at 1% based on Kernel and Stratification matching while 5% with nearest neighbor
537 matching estimators used in the study.
538 According to the result of this study on average the community assets of the PW-PSNP
539 beneficiary households contribute to the value at maximum 82 Ethiopian Birr (based on NN
540 matching) and to the minimum 54 Ethiopian Birr (based on Stratification matching) more as
541 compared to the community assets of the eligible non- beneficiary households.
542 In general, because of their participation in the program the PW-PSNP beneficiary
543 households have managed to protect their community assets from possible deterioration. Thus,
544 the program enables the PSNP beneficiary households to preserve their community assets from
545 depletion. The result coincides with [29], who assessed the impact of Ethiopia’s PSNP using
546 the method of propensity score matching method based on various impact indicators, also
547 found that the mean difference in community assets between PW-PSNP and eligible non-PSNP
548 households are positive and significant at one percent. Therefore, the result of the study showed
549 that participation in PW-PSNP was found to be helpful to the participants in improving their
550 community assets. This result is also coinciding with the finding of Yibrah [37], who studied
551 the impact of participating in the Productive Safety Net Program on Consumption and Asset
552 holding.

#### 553 3.5.3 Food Security Status of Sample Households

554 Data on food consumption was collected from 112 households for this study, aiming to capture
555 the diversity and frequency of various foods consumed within a 24-hour period. The study
556 utilized a household food consumption score, which is a continuous measure and can be divided
557 into three levels. The results indicated that 45 households (72.5%) of PW-UPSNP beneficiaries
558 had acceptable food consumption, 11 households (17.7%) had borderline consumption, and 6
559 households (9.6%) were considered poor in terms of their food consumption score. On the one
560 hand, among eligible non-UPSNP beneficiary households, 38% had acceptable food
561 consumption, 36% had borderline consumption, and 26% had poor consumption. The
562 households with poor consumption are considered food insecure, while those with borderline
563 consumption are moderately food insecure, and the households with adequate food
564 consumption are food secure. On the other hand, the mean food consumption score for PW-
565 UPSNP beneficiary households was 1.53, whereas for eligible non-PSNP beneficiary
566 households, it was 1.23. Consequently, there is a significant difference in the mean food
567 consumption score between PW-UPSNP beneficiary households and eligible non-UPSNP
568 beneficiary households, which amounts to 1.38.
569 The outcome suggests that households benefiting from PW-UPSNP are more food secure
570 compared to those eligible for PSNP but not receiving PW-UPSNP. The chi-square test
571 confirms a significant difference in food security status between the two groups at a 1% level
572 of significance (Table 8).

#### 573 3.5.4 Food Consumption Expenditure per Adult Equivalent

574 Food consumption expenditure behavior can be used to assess household food security. The
575 average food consumption expenditure for households benefiting from PW-UPSNP was
576 237.72 Ethiopian Birr, while for non-beneficiary households, it was 178.86 Ethiopian Birr. The
577 statistically significant difference of 58.86 Ethiopian Birr between the two groups was observed
578 at a 1% probability level (Table 9). Moreover, this result indicates that the PW-UPSNP on
579 beneficiary households food consumption had a positive with the maximum and the minimum
580 ATT value calculated to be 59 Ethiopian Birr (based on stratification matching) and 45
581 Ethiopian Birr (based on NN matching) respectively (Table 10).
582 582

# 583 Conclusions

584 Chronic poverty and lack of food are major global problems, particularly in developing regions.
585 In Ethiopia, around 8.5 million people, constituting 10% of the population, suffer from chronic
586 food insecurity, leading to asset selling and consumption restrictions. A study shows that
587 factors like household head's age, family size, and off-farm income activities influence
588 participation in the PW-UPSNP program. Beneficiary households have more assets than
589 eligible non-beneficiary households. The PW-UPSNP program significantly increases food
590 consumption, non-food consumption, and total consumption expenditure for beneficiaries
591 compared to non-beneficiaries.
592 Econometric analysis reveals positive effects of the PW-UPSNP program on community and
593 home asset accumulation. More PW-UPSNP beneficiaries are food secure compared to non-
594 beneficiaries. The mean food consumption expenditure is significantly higher for PW-UPSNP
595 beneficiaries. The program has a positive and statistically significant impact on food
596 consumption. The second phase of the PSNP program in Ethiopia ensures food security for
597 both chronically and temporarily food insecure households. It aims to assist over 7.5 million
598 people across eight regions through program transfers and productive activities.
599 Previous emergency food relief efforts were insufficient, leading to the implementation of
600 the PSNP program. It is the largest social protection program in Sub-Saharan Africa and
601 focuses on selected households over several years to achieve sustainable food security. Most
602 studies have focused on rural areas, but the overall conclusion is that the PW-UPSNP program
603 has a positive effect on consumption, asset accumulation, and food security. The program
604 successfully addresses chronic poverty and food insecurity in Ethiopia.
605 Future studies could examine the long-term impacts of the urban productive safety net
606 program, including its effects on household resilience, poverty reduction, and broader
607 community-level outcomes over time. Researchers could also investigate potential barriers to
608 program participation and ways to improve access and targeting for the most vulnerable urban
609 households. Comparative analyses with rural productive safety net programs could provide
610 insights into contextual factors influencing program effectiveness in urban versus rural settings.
611 Additionally, qualitative research exploring participants' experiences and perceptions could
612 complement the quantitative findings to offer a more holistic understanding of the program's
613 impacts.

##### 614 Supporting information

615 S1_Fig 1
616 S1_Table 1
617 S1_Table 2
618 S1_Table 3
619 S1_Table 4
620 S1_Table 5
621 S1_Table 6
622 S1_Table 7
623 S1_Table 8
624 S1_Table 9
625 S1_Table 10

##### 626 Acknowledgments

627 The author would like to thank farmers, agricultural development agents, and local
628 administrators of the study area for their assistance during the field work.

##### 629 Declarations

630 The author declare that he has no conflict of interests.

##### 631 Ethics Statement

632 As I mentioned in the Journal web site the reason why I gave the response N/A is because our
633 study does not have any connection with both human and animal connection. What we did is
634 collecting data from sample households via interview and focus group discussion as well as
635 field observation. All of these data collection methods do not require any kind of ethical
636 approval. Both the sample households and the key informants were expressed their consent
637 verbally to give us the response for the questions we asked about the impact of urban productive
638 safety net program on urban households’ asset accumulation and food consumption rate in
639 Dessie City. Their verbal agreement is mentioned in the methodology section of the
640 manuscript.

##### 641 Competing Interests

642 The author has declared that no competing interests exist.

##### 643 Data Availability Statement

644 Data is available within the paper and supporting information. I attached minimal data set
645 within the Supporting Information.

##### 646 Funding

647 The author received no specific funding for this work.

##### 648 Author Contributions

649 Conceptualization: Alem-meta Assefa.
650 Data curation: Alem-meta Assefa.
651 Formal analysis: Alem-meta Assefa.
652 Investigation: Alem-meta Assefa.
653 Methodology: Alem-meta Assefa.
654 Project administration: Alem-meta Assefa.
655 Resources: Alem-meta Assefa.
656 Software: Alem-meta Assefa.
657 Supervision: Alem-meta Assefa.
658 Validation: Alem-meta Assefa.
659 Visualization: Alem-meta Assefa.
660 Writing – original draft: Alem-meta Assefa.
661 Writing – review and editing: Alem-meta Assefa.
662 662
663 **REFERENCES**
664 1. Fekadu N, Mberengwa	(2009)	Challenges	of	Productive	Safety	Net	Program 665	Implementation at Local Level. The Cases of Kuyu Woreda, North Shewa Zone, Oromia 666	Region, Ethiopia. *Journal of Sustainable Development in Africa,* 11(1), 248-267.

667	2. Samuel G (2010) Food aid and small householder agriculture in Ethiopia development

668	studies, March 20-22.

669	3. World Bank (2012) Retrieved from; Washington, D.C: 2012. Managing risk, promoting 670	growth: Developing systems for social protection in Africa‐‐the World Bank’s Africa 671	social protection strategy 2012-2022. [[Google Scholar](https://scholar.google.com/scholar?q=World%2BBank%2BManaging%2Brisk%2C%2Bpromoting%2Bgrowth%E2%80%AF%3A%2BDeveloping%2Bsystems%2Bfor%2Bsocial%2Bprotection%2Bin%2BAfrica%E2%80%90%E2%80%90the%2BWorld%2BBank%E2%80%99s%2BAfrica%2Bsocial%2Bprotection%2Bstrategy%2B2012-2022%2B2012%2BRetrieved%2Bfrom%2BWashington%2C%2BD.C%2B)]

672 4. FAO, IFAD, UNICEF, WFP and WHO (2023) The State of Food Security and Nutrition in 673	the World 2023. Urbanization, agrifood systems transformation and healthy diets across 674	the rural–urban continuum. Rome, FAO. <https://doi.org/10.4060/cc3017en>

675 5. Food and Agriculture Organization of the United Nations (2023) *The State of Food Security* 676	*and	Nutrition	in	the	World	2023*.Rome: FAO. Retrieved	from 677	<https://www.fao.org/publications/sofi/2023>

678 6. Africa Center for Strategic Studies (2023) Unresolved Conflicts Continue to Drive Africa’s 679	Food Crisis. africacenter.org/spotlight/unresolved-conflicts-continue-to-drive-africas- 680	food-crisis/

681	7. Ministry of Agriculture and Rural Development (MOARD) (2010) Concept Note for

682	Establishment of a System of Productive Safety Net in Ethiopia.

683	8. Berhane G, J Hoddinott, Kumar H, Alemayehu S, Michael T, Yisehac Y et al. (2013) 684	‘Evaluation of Ethiopia’s Food Security Program: Documenting Progress in the 685	Implementation of the Productive Safety Nets Program and the Household Asset 686	Building Programm’. ESSP II–EDRI REPORT, Washington, D.C: International Food 687	Policy Research Institute.

688	9. Gilligan D.O, Hoddinott J, Seyoum T.A (2008) *The impact of Ethiopia's Productive Safety*

689	*Net Programme and its linkages***.** International Food Policy Research Institute (IFPRI).

690 10. Berhane G, Gilligan D.O, Hoddinott J, Kumar N, Taffesse A.S (2014) Can social protection 691	work in Africa? The impact of Ethiopia's Productive Safety Net Programme. *Economic* 692	*Development and Cultural Change,* 63(1), 1-26.

693 11. Federal Democratic Republic of Ethiopia (FDRE) (2006) Productive Safety Net Program: 694	Program Implementation Manual (rev). Ministry of Agriculture and Rural Development 695	(MoARD), Addis Ababa, Ethiopia.

696	12. Federal Democratic Republic of Ethiopia (FDRE) (2001) Rural Development Policies,

697	Strategies and Instruments of the Government of the FDRE.

698 13. Berhane G, J Hoddinott, Kumar N, Alemayehu S (2011) The impact of Ethiopia’s 699	Productive Safety Nets and Household Asset Building Programme: 2006-2010. 700	Washington, D.C: International Food Policy Research Institute.

701 14. Ministry of Urban Development and Housing UPSNP (2016) Urban Productive Safety Net
702 Program Implementation Manual, Version xx, Addis Ababa.
703 15. Dessie City UPSNP Task Force Office (2019) Unpublished Report Document, Ethiopia.
704 16. Zewdie Y.M (2014) "Food Insecurity and Coping Strategies of Urban Poor Households in
705 Addis Ababa." *Journal of Economics and Sustainable Development,* 5(20): 20-30.
706 17. Andersson C, Alemu M, Stage J (2010) Impacts of the Productive Safety Net Program in 707	Ethiopia on livestock and tree holdings of rural households Environment for 708	Development Discussion Paper Series 09-05.

709	18. Devereux S, Sabates-Wheeler R (2004) Transformative social protection. IDS Working

710	Paper, 232. Institute of Development Studies, Brighton, Sussex Bn1 9RE England.

711

| 712 | 19. Kataru K (2011) Assessment of the Impact of Productive Safety Net Program on the Lives |
| --- | --- |
| 713 | of the Beneficiaries in Ethiopia: The Case of Dera Malo Woreda, Gamo Gofa Zone, |
| 714 | unpublished Master of Arts Degree in Public Management, Addis Ababa University, |
| 715 | Ethiopia. |
| 716 | 20. Berhane G, J Hoddinott, Kumar N, Alemayehu S (2011) The impact of Ethiopia’s |
| 717 | Productive Safety Nets and Household Asset Building Programme:2006-2010. |
| 718 | Washington, D.C: International Food Policy Research Institute. |
| 719 | 21. Tadele M (2011) Impact of Productive Safety Net Program on the sustainable Land |
| 720 | Management Practice in Central Rift value. University of Haramaya, Ethiopia. |
| 721 | 22. Andersson C, Alemu M, Jesper S (2011) Impacts of the Productive Safety Net Program in |
| 722 | Ethiopia on livestock and tree holdings of rural households. *Journal of Development* |
| 723 | *Economics*, 94 (2011) 119–126. doi:10.1016/j.jdeveco.2009.12.002. |
| 724 | 23. Habitamu A (2011) Impact of Productive Safety Net Program on the Household Welfare |
| 725 | and labor supply in rural Ethiopia. Master’s Thesis, Addis Ababa University, Ethiopia. |
| 726 | 24. Central Statistics Authority (CSA) (2007) National Population Statistics. Federal |
| 727 | Democratic Republic of Ethiopia, Central Statistical Authority, Addis Ababa, Ethiopia. |
| 728 | 25. Kombo DK, Tromp DL (2006) *Thesis Writing*: An Introduction, Paulines Publications |
| 729 | Africa, Nairobi. |
| 730 | 26. Creswell JW (2009) *Research Design: Qualitative, Quantitative, and Mixed Methods* |
| 731 | *Approaches.* 3^rd^Edition, SAGE Publications, London, United Kingdom. |
| 732 | 27. Kothari CR (2004) *Quantitative Techniques*. 2^nd^Edition, New Delhi: Vikas Publishing |
| 733 | House Pvt. Ltd. India. |
| 734 | 28. Gujarati DN (2004) *Basic Econometrics*. 4^th^Edition, the McGraw−Hill Companies. |
| 735 | 29. Gilligan DO, Hoddinott J, Alemayehu S (2008) *An analysis of Ethiopia’s Productive Safety* |
| 736 | *Net Program and its linkages*. International Food Policy Research Institute, Washington, |
| 737 | DC. |
| 738 | 30. Rosenbaum PR, Rubin DB (1983) The Central Role of the Propensity Score in |
| 739 | Observational Studies for Causal Effects. *Biometrika*,70, 41-55. |
| 740 | <http://dx.doi.org/10.1093/biomet/70.1.41> |
| 741 |  |
| 742 | 31. Gebrehiwot T, Castilla C (2019) "The Role of the Productive Safety Net Programme |
| 743 | (PSNP) in Improving Consumption and Asset Accumulation in Rural Ethiopia." |
| 744 | *Journal of Development Studies,* 55(5): 685-700. |
| 745 | 32. Gilligan Daniel O, Hoddinott John (2007) "Is There Persistence in the Impact of Emergency |
| 746 | Food Aid? Evidence on Consumption, Food Security, and Assets in Rural Ethiopia." |
| 747 | *American Journal of Agricultural Economics,* 89(2): 225-242. |

748	33. Gebrehiwot T, Van der Veen A (2015) "Coping with Food Insecurity on a Micro-scale: 749	Evidence from Ethiopian Rural Households." *Ecology of Food and Nutrition*, 54(2): 750	187-208.

751 34. Derso A, Bizuneh H, Keleb A, Ademas A, Adane M (2021) Food insecurity status and 752	determinants among Urban Productive Safety Net Program beneficiary households in 753	Addis	Ababa,	Ethiopia.	*PLOS	ONE,*16(9):e0256634.https://doi. 754	org/10.1371/journal.pone.0256634

755	35. Alemseged G, Hailu B (2018) Determinants of rural household food insecurity in Northern

756	Ethiopia: An empirical study. *Sustainable Development Africa*, 2018; 20(1):32–63. 757	36. Scho L, Wekesah F, Mohamed S (2014) Vulnerability to food insecurity in urban slums: 758	Experiences from Nairobi, Kenya. *Journal of Urban Health*, 2014; 91(6):1098–113.

759	37. Yibrah H (2010) Impact of Productive Safety Net Program on the Rural Households asset

760	Protection and Consumption. Unpublished MA Thesis, Mekelle University, Ethiopia.

761

762

Revised Manuscript with Track Changes

1 Impact of Urban Productive Safety Net Program on Urban
2 Households’ Asset Accumulation and Food Consumption

## 3 Rate in Dessie City, South Wollo Zone, Amhara Region,

4 Ethiopia
5 ^1^Bezaye Endris, ^2^Alem-meta Assefa

6

7 ^1^Dessie City Municipal office, Dessie, Ethiopia
8 ^2^Department of Geography and Environmental Studies, Wollo University, P.O. Box 1145,
9 Dessie, Ethiopia

10

11

12 Corresponding author’s email: [alemmeta99@yahoo.com](mailto:alemmeta99@yahoo.com)
13 ^1^Wollo University, Department of Geography and Environmental Studies, P.O. Box 1145,
14 Dessie, Ethiopia
15 Corresponding author’s ORCID: [https://orcid.org/0000-0002-1856-](https://orcid.org/0000-0002-1856-2373)

16	[2373](https://orcid.org/0000-0002-1856-2373)

17

18

19

20

21

##### 22 Abstract

23 Agriculture is the driver of economic growth in many developing countries like Ethiopia,
24 where it often represents at least 25% of gross domestic product. But the quality and
25 productivity of the land depends on the way agriculture is practiced. Better agricultural
26 techniques are necessarily including Productive Safety net Program. Urban Productive Safety
27 Net Program is designed to support the poor households and establish urban safety net
28 mechanisms. The main objective of this study was to assess the impact of urban productive
29 safety net program on asset accumulation and consumption rate in Dessie city. Both primary
30 and secondary data sources were used. A total of 112 households were selected based on
31 simple random sampling method among the public work Urban Productive Safety Net
32 Program	beneficiary	and	eligible	non-beneficiary	households.	Descriptive	analysis,
33 inferential statistics and econometric models were used for analysis. Based on the
34 econometric estimation results the demographic and socio-economic variables such as
35 education, access to credit, access to extension services and adult male labor force revealed
36 positive relation to public work urban productive safety net program beneficiary and non-
37 beneficiary household’s asset accumulation and consumption. The impact of program
38 participation was also found to be positive and significant for both home asset and
39 community asset. While food and non-food consumption even the total consumption and
40 food security status are also positive. Generally, the result indicated that, due to program
41 participation beneficiary households have higher home asset and community asset and better
42 consumption and food security status.
43 **Keywords:** Productive safety net program; asset accumulation; consumption; Dessie City

44

45

# 46 1. Introduction

47 In Sub-Saharan Africa, numerous individuals continue to experience chronic food insecurity
48 caused by repeated droughts, limited productivity in subsistence agriculture, and widespread
49 poverty [1]. Samuel [2] states that various food security initiatives have been created to
50 address this ongoing issue effectively. Accordingly, in the past two decades we have seen a
51 rapid increase of social protection programs in African countries to alleviate poverty, food
52 insecurity, and vulnerability of poor households [3]. These formal programs aim to assist
53 individuals and households facing chronic food insecurity or temporary financial setbacks by
54 offering income support or alternatives, such as cash and in-kind transfers, subsidies, and
55 labor-intensive public work. The Productive Safety Net Program (PSNP) is a large-scale
56 social protection program implemented in Ethiopia since 2005. It aims to provide assistance
57 to chronically food-insecure households and help them improve their food security and
58 resilience to shocks. The program combines two main components: direct support through
59 cash and/or food transfers to eligible households, and support for public works programs. The
60 cash and food transfers are intended to meet immediate consumption needs, while public
61 works programs provide temporary employment opportunities and help build community
62 assets such as roads, bridges, and irrigation systems.
63 The relative lack of change in hunger at the global level from 2021 to 2022 hides
64 substantial differences at the regional level. Progress was made towards reducing hunger in
65 most subregions in Asia and in Latin America, but hunger is still on the rise in Western Asia,
66 the Caribbean and all subregions of Africa. The proportion of the population facing hunger is
67 much larger in Africa compared with the other regions of the World – nearly 20 percent
68 compared with 8.5 percent in Asia, 6.5 percent in Latin America and the Caribbean, and 7.0
69 percent in Oceania [4].
70 An estimated 149 million Africans are facing acute food insecurity—an increase of 12
71 million people from a year ago. This equates to a risk category of 3 or higher (Crisis,
72 Emergency, and Catastrophe) on the Integrated Food Security Phase Classification (IPC)
73 scale of 1 to 5. Some 122 million of those facing acute food insecurity are in countries
74 experiencing conflict—82 percent of the total—accentuating that conflict is the primary
75 driver of acute food insecurity in Africa [5]. Four of the top 10 countries facing the most
76 acute food insecurity are in East Africa—Sudan, Ethiopia, South Sudan, and Somalia.
77 Ethiopia, in particular, had an estimated 20.1 million people who are facing food insecurity,
78 and 15.1 million require emergency food assistance, following a 2-year war in Tigray,
79 conflicts in other parts of the country, as well as a severe drought that struck the Horn of
80 Africa [5]. These individuals lacked consistent access to sufficient food, leading to asset
81 selling and consumption restrictions in order to survive [6].
82 Before implementing PSNP, the Ethiopian 69 government relied on unpredictable annual
83 appeals for emergency food relief, which saved lives but failed to 70 address the increasing
84 number of food insecure individuals [6]. Based on the information provided, the study
85 conducted by Berhane et al. [7] indicates that the second phase of the Productive Safety Net
86 Program (PSNP) in Ethiopia has several successes when compared with the initial emergency
87 relief appeal method. These successes include:
88 **Enhanced food security**: The study suggests that the PSNP plays a crucial role in ensuring
89 food security for both chronically and temporarily food insecure households. By providing
90 program transfers and supporting productive activities, the PSNP aims to assist over 7.5
91 million people in 318 districts across eight regions. This targeted approach is likely more
92 effective in addressing the needs of food insecure individuals compared to the unpredictable
93 annual appeals for emergency food relief.
94 **Long-term planning and stability:** Unlike the initial emergency relief appeal method, which
95 relied on unpredictable annual appeals for food aid, the PSNP operates on a set timeline until
96 December 2014. This longer-term planning allows for more stability and predictability in
97 addressing food insecurity issues.
98 **Diversification of support:** The PSNP not only provides program transfers but also supports
99 productive activities. This indicates that the program aims to go beyond providing immediate
100 relief by helping households develop sustainable livelihoods and improve their resilience to
101 food insecurity.
102 However, without further information on the specific failures of the initial emergency
103 relief appeal method, it is challenging to make a direct comparison. It is important to note that
104 the PSNP is a complex program with its own limitations and challenges. Further analysis and
105 evaluation would be needed to understand the full extent of its successes and failures in
106 comparison to the initial emergency relief appeal method.
107 In an attempt to break the cycle of annual appeal of food aid and achieve an acceptable
108 level of food security at macro (national) and micro (household) level, the government of
109 Ethiopia developed a food security strategy (FSS) in November 1996 [8]. The FSS
110 highlighted government plans to address the causes and effects of food insecurity in Ethiopia.
111 Based on the FSS, the government designed regional food security programs and projects in

112	2002 [9].

113 The PSNP is currently the largest operating social protection program in Sub-Saharan
114 Africa, outside of South Africa. It differs from previous food-for-work programs, in that it
115 focuses continuously on selected households over several years and in that the explicit
116 objective was that it will eventually be phased out [10].
117 The Ethiopian government has been working on a program called the Urban Productive
118 Safety Net Program (UPSNP) that will span a 10-year period. This program is part of the
119 Urban Food Security and Job Creation Strategy, which was officially endorsed on May 8,
120 2015. The purpose of the UPSNP is to provide assistance to more than 4.7 million
121 impoverished individuals living in 972 cities and towns across the country. It is envisaged
122 that this will be achieved over a long-term period through a gradual roll-out plan of different
123 phases starting with big cities having a population of over 100,000 people. The proposed
124 Bank support will provide assistance for the first five-year phase of the government program
125 and is targeting 11 major cities, Addis Ababa and one city from each region (Adama,
126 Assayita, Asosa, Dessie, Dire Dawa, Gambella, Hawassa, Harari, Jijiga, and Mekele). In the
127 initial stage of the UPSNP, approximately 604,000 individuals who are among the most
128 impoverished 12 percent and around 55 percent of the population residing below the poverty
129 threshold in these 11 cities were the recipients of the program's benefits [11].
130 Dessie City was one of chronically food insecure cities in Ethiopia. The first phase of the
131 program started in 2017 through direct support and public work by choosing beneficiaries
132 from selected *Kebeles* [12]. Hence, Dessie City has been labeled as typical food insecure area
133 despite various food security interventions with PSNP is effective or not. Having these in
134 mind, the researchers tried to evaluate the impacts of urban productive safety net program on
135 urban households’ asset accumulation, consumption and food security status in Dessie City.
136 Accordingly, the conceptual framework shows several factors; like personal, socio-economic,
137 institutional and bio-physical factors that affect household participation in UPSNP and its
138 impact (Fig 1).

##### 139 Fig. 1 Conceptual Framework of the Study

140 Historically, urban poverty has consistently faced the significant challenge of food
141 insecurity, especially in drought-prone areas of Ethiopia. The responses to food security have
142 conventionally been dominated by emergency food-based intervention. High proportion of
143 food insecure households have been receiving emergency food aids. Although the emergency
144 assistance was substantial and saved many lives, there was uncertainty of the food aid, poor
145 timing of provision and insufficient quantity of aid for the individual’s beneficiary was
146 among the main defects of food aids in the past and creating dependency syndrome on the
147 recipients [13]. For that reason, the past decades of large-scale food aids deliveries have done
148 little to prevent households’ asset depletion, prevent environmental degradation and
149 chronically food insecurity in general, because of ignorance of integrating these aids with
150 natural resource management in the country.
151 Recognizing this, the government of Ethiopia changed the emergency food-based
152 assistance to multi-year support of PSNP in 2005 that provides cash and food transfers to
153 chronically food insecure households with the aim of preventing asset depletion at household
154 level while creating assets at the community level [14]. At national level, there were some
155 works that tried to highlights the impacts of program intervention, particularly PSNP.
156 Unfortunately, most of them have focused on the impacts of PSNP on asset building and
157 consumption in rural areas rather than urban areas. According to Kataru’s [15] research
158 finding, it has been determined that the Productive Safety Net Program (PSNP) is capable of
159 effectively mitigating consumption fluctuations and safeguarding household assets. Berhane
160 et al. [16] indicated that beneficiary households have managed to significantly improve their
161 food security status by 1.05 months. On the contrary, what has been demonstrated is that there
162 is no noteworthy distinction between the consumption and assets of individuals involved in the
163 PSNP program and those who are not [17]. Similarly, Andersson et al. [18] indicated that
164 there was no increment in livestock holdings for program participants. Furthermore,
165 Habitamu [19], stated that, participation in PSNP reduces the asset size of the participants,
166 due to the dependency behavior of beneficiaries which discourage them to put efforts on their
167 agricultural activities. But, none of them discussed the magnitude of the impact of PSNP on
168 asset accumulation and consumption on urban households.
169 From the above statements, we understand that various researchers have examined the
170 effects of the program at both the national and local levels, but their findings have been
171 inconsistent and far from conclusive. Consequently, this study was conducted to address this
172 gap.

# 173 2. Materials and Methods

### 174 2.1 Description of the Study Area

175 Dessie City is aged more than a hundred and ten years old and also capital of Dessie Zuria
176 *Wereda* and South administrative Wollo Zone. It is located between 11^0^08’North Latitude
177 and 39^0^38’East Longitude. It is also situated in the north eastern part of the country at 401km
178 away from Addis Ababa, the capital of Ethiopia. The city is placed at the foot of Tossa Ridge
179 and is surrounded by and also incorporates a number of hills within its boundary. The average
180 elevation of the city is 2,600 m.a.s.l. Dessie was established in 1893 by King Michael. The
181 city serves as social, economic and political center for the South Wollo Zone and their
182 administration. According to the Central Statistics Agency [20], the total population of the
183 city is approximately 250,346 and its annual growth rate is 3.72%. The sex composition of
184 the population is 46.8% male and 53.2% female. The city has 5 sub-cities and its total area is

185	16,745.82 hectares.

186

187

188

189 **2.2 The Research Design and Sampling**
190 The research was conducted through descriptive survey design. The survey design helps to
191 identify patterns of behavior, activities, people’s attitude, opinions, habits and any of the
192 variety of social problems that influence the explicability and scaling-up efforts of the
193 program across various contexts [21]. The utilization of a descriptive survey design aids in
194 identifying past occurrences and enables the pre-determination of specific preventive
195 measures that can be implemented.
196 The study used mixed research approach. Accordingly, both quantitative and qualitative
197 data were collected and analyzed at the same time. Data obtained through interview,
198 questionnaire and observation were analyzed qualitatively. The quantitative data were
199 analyzed and interpreted through appropriate statistical tools and cross tabulations were made
200 to facilitate meaningful analysis and interpretations of the research findings.
201 **Selection of Sample Site:** On the bases of the researchers’ prior knowledge of the area and
202 work place Dawudo Sub-city was selected purposively among the 5 sub-cities of Dessie City.
203 Then, in Dawudo sub-city, Robit and Bahil Amba *Kebeles* were selected by simple random
204 sampling method.

##### 205 Selection of Sample Households

206 Creswell [22], stated that all target populations are member of a real set of subjects to which a
207 researcher wishes to generalize the results of the study. The study specifically focused on
208 each chosen Kebele, and the researcher obtained the complete roster of household heads from
209 the Kebele administration office.
210 Then on the bases of *Kebele* document there are 338 Public Work-Productive Safety Net
211 Program (PW-PSNP) beneficiary households and 272 eligible non-beneficiary households in
212 Robit *Kebele*. While 337 PW-PSNP beneficiary households and 273 eligible non-beneficiary
213 households in Bahil Amba *Kebele,* thus totally 1220 both in PW-UPSNP beneficiary and
214 eligible non-beneficiary household heads were used as sample frames included for the
215 survey. Then after, the researcher used the known formula so as to minimize the number of
216 household heads in the study as a sample. Thus, Kothari [23] sample size determination was
217 employed as shown below:
218 218

z^2^.p.q.N

e^2^(N−1)+Z^2^.p.q

n =

219 Where: n = Sample size
220 z =Standard variation at 95 percent confidence interval (1.96)
221 p = Sample proportion in the target population estimated to have the characteristics being
222 measured (0.03)

223	q = 1-p

224 N =size of the target population is 1220
225 e= the estimate should be within 3 percent of the true value (0.03)
226 226

Therefore, the desired sample is n =	^1.96^2.0.03.(1−0.03).(1220)	= 112.4

0.03	1219 −1 +(1.96) .0.03.(1−0.03)

2(	)	2

227 227

0.03(1.96^2^ x0.97x1220)

n= 0.03(0.03 𝑥 1219+1.96^2^)

3.8416𝑥0.97𝑥1220	= n=

36.57+3.8416

=

4546.14944	=112

40.4116

228	n =**112**

229 Then by using the above formula, 112 household heads were selected as a representative
230 sample. These household heads were selected based on proportional random sampling
231 technique or ratio to the total household heads (Table 1). In addition to this, interviews were
232 conducted with six key informants, specifically selected *Kebele* Development Agents, *Kebele*
233 Administrators, and the head of the UPSNP Task Force members. Finally, both the sample
234 households and the key informants were orally asked to express their agreement for data
235 collection interview. As a result, they expressed their consent verbally.

##### 236 Table 1.1 Sampling Frame and sample size of household heads.

| **Dawudo sub city** | **Target population** | | | **Size of sample households** | | |
| --- | --- | --- | --- | --- | --- | --- |
|  | PWPSN P  beneficia ry | Eligible- UPSNP  non- beneficiary | Total | PWPSNP  Beneficiary | UPSNP non- beneficiary | Total |
| **Robit Kebele** | 338 | 272 | 610 | 31 | 25 | 56 |
| **Bahilamba Kebele** | 337 | 273 | 610 | 31 | 25 | 56 |
| **Total** | 675 | 545 | 1220 | 62 | 50 | 112 |

237 237

### 238 2.3 Data Sources and Collection Techniques

239 The study employed both primary and secondary data sources. The primary data were
240 collected from public work UPSNP beneficiary and eligible non-beneficiary household heads
241 through questionnaires as well as key informant interview. While secondary data were
242 collected from different published and unpublished sources of data. Data collection
243 techniques of the study include questionnaires, key informant interview, and field
244 observation.

### 245 2.4 Methods of Data Analysis

246 The study employed both quantitative and qualitative data analysis techniques. Quantitative
247 data were gathered through the close-ended questionnaire and analyzed by using descriptive
248 statistics, inferential statistics and econometric tools using stata computer software. Similarly,
249 qualitative data was gathered through open-ended questionnaires and interview and analyzed
250 textually to supplement the questionnaire survey.
251 To assess the distribution of the covariates between the two groups, the quantitative data
252 underwent analysis using descriptive techniques. This involves examining the independent
253 variables through methods such as calculating frequencies and percentages, which were then
254 summarized in tables to interpret and draw conclusions from the results. Inferential statistical
255 tools, including mean, Chi-square test, one-sample t-test, and paired t-test, were employed to
256 determine the significance of mean values for continuous variables between the households
257 of PW-PSNP beneficiaries and eligible non-beneficiaries. To estimate the impact of
258 household’s participation in the Urban Productive Safety Net program on asset accumulation
259 and consumption expenditure logit model and an econometric model (Propensity score
260 matching method) were used in addition to the simple descriptive analysis.

##### 261 Ethics Statement

262 As I mentioned in the Journal web site the reason why I gave the response N/A is because our
263 study does not have any connection with both human and animal connection. What we did is
264 collecting data from sample households via interview and focus group discussion as well as
265 field observation. All of these data collection methods do not require any kind of ethical
266 approval. Both the sample households and the key informants were expressed their consent
267 verbally to give us the response for the questions we asked about the impact of urban
268 productive safety net	program	on	urban	households’	asset	accumulation	and	food
269 consumption rate in Dessie City. Their verbal agreement is mentioned in the methodology
270 section of the manuscript.

##### 271 2.4.1 Binary Logistic Regression Model

272 According to Gujarati [24], in estimating logit model for the dichotomous (binary) logistic
273 regressions was used to achieve this objective, particularly; participation which takes a value
274 of 1 if the household participated in a program and 0 otherwise. The logit model was selected
275 for this study because the dependent variable is dichotomous in nature and the computation
276 will be easier. For the binary treatment case, where we estimate the probability of participants
277 versus non-participants of UPSNP is addressed as a decision involving binary/dichotomous
278 response variable. The logit model is mathematically formulated as follow:
279 279

Pi =

𝑒𝑍𝑖

zi	**(1)**

1+𝑒

280 Where, Pi -is the probability of participation in the PSNP in relation to explanatory variable
281 ezi- irrational number to the power of Zi
282 Zi = βo + Σ Βi Xi + Ui	**(2)**
283 Zi - a function of n explanatory variable 1, 2…	n )
284 βo - is the intercept
285 Βi- is regression coefficients to be estimated
286 Xi- is pre-intervention characteristics (explanatory variable)
287 Ui- is a disturbance term
288 The probability that the household belongs to non-participant group is given by:
289 289

1-Pi =

1 zi	**(3)**

1+𝑒

290 Then the odd ratio was written as:
291 291

Pi 1−Pi =

1+𝑒𝑧𝑖 _zi 𝑒

1+𝑒

=

𝑧𝑖

**------------------------------------------------------------------------------(4)**

Pi

292 . It is simply the odds ratio in favor of participating in the UPSNP. It is the ratio of the

1−𝐏𝐢

293 probability that the household would participate in the UPSNP to the probability that he/she
294 would not participate in the UPSNP.
295 By taking the natural log of equation (4), the log of odds ratio was written as:
296 296

Li = Ln ( ^Pi^

1−𝐏𝐢

) = Ln ( 𝑒^𝑧𝑖^ ) = Zi = βo + Σ ΒiXi + Ui	(**5**)

297 Li- is the log of the odds ratio in favor of participation in UPSNP which is linear both in Xi
298 and parameters.
299 **2.4.2 Propensity Score Matching (PSM) Method**
300 As placement to participate in PSNP is not random, i.e., an eligible household is deliberately
301 selected based on asset size, vulnerability to shocks and food insecurity.	In addition, the
302 baseline survey was not conducted prior to the intervention of the PSNP in the study area.
303 Therefore, propensity score matching method is usually used to assess the impact of a
304 program, in this case, we used to address the impacts of PSNP on asset accumulation and
305 consumption in the beneficiary households.
306 According to Gilligan et al. [25] this model assumes that, “after controlling for all pre-
307 intervention observable household’s and community’s characteristics that are correlated with
308 both program participation and the outcome variable, untreated groups have the same average
309 outcome as the treated group would have had they did not receive the program”. This is the
310 basic idea, to found in group of non-participants those individuals who are similar to the
311 participants in all relevant pre-treatment characteristics X. Hence, the matching approach is
312 one of the best solutions.
313 **Procedures to estimate propensity score:** It is the first step of PSM technique. Rosenbaum
314 and Rubin [26] revealed that matching can be performed conditioning only on P(X) rather
315 than on X, where P(X) = Prob (D=1|X) is the probability of participating in the program
316 conditional on X. According to these authors, if outcomes without the intervention are
317 independent of participation given X, then they are also independent of participation given
318 P(X). Estimating the propensity score involves decision on three choices: what model to be
319 used for the estimation and what variables should be included in this model and matching
320 algorism.

##### 321 Impact Indicator Variables for Asset Accumulation and consumption

322 The impact of the PSNP is analyzed by dividing the two categories which are the asset
323 building and consumption expenditure of the PW-PSNP beneficiary and eligible non-
324 beneficiary households.
325 **Outcome Variables for Asset Accumulation:** The first is the home asset (which is the sum
326 of household goods and consumer durables) and community asset value is also measured
327 based on the estimation of its market value by respondents in terms of Birr.
328 **Outcome variables for consumption**: In order to analyze the impacts of the PSNP on the
329 household’s consumption the study has attempted to examine it’s by classifying the
330 households’ consumption into two main categories, because households have consumed
331 different items and due to this reason, their value might not the same. Therefore, in the study,
332 the households have been asked about the quantity as well as the current price of overall
333 households mean real food consumption expenditure per Adult Equivalent (AE) in terms of
334 24 hours and current non-food consumption expenditure per adult equivalent (AE) in terms of
335 year, at their disposal, during the field survey based on the respondents’ consumption rate in
336 terms of Ethiopian Birr.

# 337 3. Results and Discussion

### 338 3.1 Descriptive Analysis of the Dummy Variables Used in Logit

339 **Model**
340 **Sex of the household head:** In the urban area, female headed households are more effective
341 in feeding their family in a sustainable manner from year to year which makes them more
342 likely to be food secure. Because, female headed households are in a better labor input than
343 the males headed in urban area. Based on the respondents result in this study, the sample was
344 composed of both male and female headed households from the total sample households 94
345 (84%) and 18 (16%) were male and female headed household respectively. From the total
346 male headed household respondents, that have been included in the study, 54 (48%) are from
347 the PW-PSNP participant group while the other 40 (36%) are from the non-PSNP
348 participants. In other way female headed households represent about 8 (7%) and 10 (10%)
349 from PW-PSNP participant and non-PSNP beneficiary households respectively. As the result
350 indicates that, in the study area the male headed households were a higher chance to PSNP
351 participants in the public work components of the PSNP. Therefore, as compared to their
352 female headed households’ counterpart male headed households participate more in the
353 PSNP.
354 **Credit Experience**: It is indicating whether the household had credit experience or not.
355 Those who had credit experience can use it to purchase something so that they would manage
356 to escape the liquidity constraints experienced by many urban households in the study area.
357 Moreover, the credit could also serve as a capital source to fund any off-farm activity that the
358 households may undertake indicating that credit experience minimizes the danger of being
359 food unsecured. Amhara Credit and Saving Institution is one of the main and dominant credit
360 suppliers in the study area. Prior to PSNP of the respondents only 11 (10%) PW-PSNP
361 participant and 7 (6%) of non-PSNP participants had taken a loan. Prior to PSNP shows that
362 about 51 (46%) of respondents and 43 (38%) of non-beneficiary respondents had not
363 borrowing credit experience. Access to credit after the implementation of the program
364 indicated that out of the total respondents 38 (34%) of them were public work of PSNP
365 beneficiaries and 33 (29%) non-beneficiaries households received credit, mainly to purchase
366 food item, and improve household asset. It indicates that, higher percent of PSNP public
367 work participants had access to credit compared to non-participant groups. Hence, after the
368 program, from the total respondents of public work PSNP beneficiary have been better and
369 borrowing experience than non-beneficiary household. It indicates that greater percentage of
370 beneficiary households mainly to purchase food item and improve home asset compared to
371 non-beneficiary households. Moreover, the survey result revealed that out of the total
372 respondents with off-farm income, 51 (46%) of them were from the participant group and the
373 41 (37%) were non-participant households. A study done by Derso et al. [27] supported this
374 result. It indicated that three in four households were experiencing food insecurity and that an
375 appropriate multi-dimensional approach such as the opening of small business enterprises
376 through credit service support may help to diversify income sources and to reduce the
377 dependency ratio. While, without off-farm income, 11 (10%) of them were from the
378 participant group and the others 9 (8%) were found to be non-participant households;
379 regarding access to extension service about 55 (49%) of PSNP in public work participants
380 and 33 (29%) of non-participants were receiving the type of training in 2019/20. But, the
381 remaining 7 (6%) of PSNP participants and 17 (15%) non-participants did not receive any
382 type of training. Based on these results, to some extent the extension service was highly
383 supportive to the PSNP beneficiary households when compared with non-beneficiary
384 households. According to the survey result the educational status of the household revealed
385 that 31 (27.6%) of PW-PSNP beneficiary households were literate (able to read and write)
386 and 25 (22%) of non-beneficiary households were literate (able to read and write). While, 31
387 (27.6%) and 25 (22%) of PW-PSNP beneficiary and PSNP non-beneficiary household heads
388 were illiterate (unable to read and write) respectively. The result indicated that, both illiterate
389 and literate household heads in the respondent were similar in proportion (Table 2).
390 390
391 391
392 392
393 393

**Table 2. Descriptive Analysis of the Dummy Variables Used in the Study.**

| **Explanatory Variables** | **All Samples** | **PSNP Categories** | | | |
| --- | --- | --- | --- | --- | --- |
|  |  | **UPSNP beneficiary HHs** | | **Non-UPSNP beneficiary HHs** | |
|  |  | **Frequency** | **Percentage** | **Frequency** | **Percentage** |
| Sex of household heads | Male (n= 94) | 54 | 48 | 40 | 35.7 |
|  | Female (n=18) | 8 | 7 | 10 | 9 |
| Access to credit before the  implementation of the program | Yes (n=18) | 11 | 10 | 7 | 10 |
|  | No (n=94) | 51 | 45.5 | 43 | 38 |
| Access to credit after the implementation of the  program | Yes(n=71) | 38 | 33.9 | 33 | 29.4 |
|  | No(n=41) | 24 | 21.4 | 17 | 15 |
| Off-farm income before  program | Yes (n=92) | 51 | 45.5 | 41 | 36.6 |
|  | No (n=20) | 11 | 10 | 9 | 8 |
| Access to extension services and training | Yes (n=88) | 55 | 49 | 33 | 29.46 |
|  | No (n=24) | 7 | 6.25 | 17 | 15 |
| Household head education | Literate (n=56) | 31 | 27.6 | 25 | 22.3 |
|  | Illiterate (n=56) | 31 | 27.6 | 25 | 22.3 |
| Access to remittance | Yes (n=30) | 19 | 17 | 11 | 10 |
|  | No (n=82) | 43 | 38 | 39 | 35 |
| Experienced food gap | Yes (n=106) | 58 | 52 | 48 | 43 |
|  | No ( n=6) | 4 | 3.5 | 2 | 1.7 |

394 On the other hand, access to remittance the result of the study showed that about
395 51(45.5%) of the PW-PSNP beneficiary households did not receive any kind of remittance,
396 and the rest of 11 (10%) received remittance mainly to purchase food item and none food
397 items. while	from the total none beneficiary households, 11 (10%) household heads who
398 took remittances and the remaining 39 (35%) non-beneficiary household heads did not
399 received remittance respectively. This means greater percentages of beneficiary of the
400 UPSNP are not received remittance as compared to non-beneficiary. Besides, the result of the
401 study also revealed that food gap faced households prior to the introduction of the program
402 three month and above were faced food gap, 58 (52%) and 48 (43%) of PW-PSNP
403 beneficiary and non-PSNP beneficiary households’ groups had food gap on three months of
404 experienced food gaps respectively. It implies that prior to the introduction of the program
405 higher percentage of participant groups were faced food gap and food insecure compared to
406 non-beneficiary households (Table 2 above).

### 407 3.2 Descriptive Analysis of the Continuous Variables Used in

408 **Logit Model**
409 **Age of the household head:** It is a continuous variable that refers to the level of `experience
410 the farmers possess and measured in the number of years. The older the head of the
411 household is the more he/she would be effective in risk diversification as well as in predicting
412 the weather and applying the right technology. However, the existence of large number of
413 children under the age of 15 and old age of 65 and above in the family affect the household
414 consumption negatively. The households with higher dependency ratio will have lowest
415 welfare status. This means households with higher dependency ration had low level of
416 welfare, hence higher probability to fall in to food in security and poverty. The result of the
417 study indicated that the average age of the household head for the whole sample is around
418 49.17 years (Table 3). When we compare the two groups in terms of their mean age, it was
419 found that treated groups are older than their controlled groups by about 1.58 year, i.e., 49.91
420 years compared to 48.33 years respectively. Thus, the older household heads were found to
421 be food in secured.
422 **Table 3. Descriptive Analysis of Continuous Variables Used in the Logit Model.**

| **Variables** | **All Samples** | **Beneficiary** | **Non-beneficiary** | **Mean Difference** | **T-value** |
| --- | --- | --- | --- | --- | --- |
| Age of the Household  Heads | 49.17 | 49.91 | 48.33 | 1.58 | 0.97 |
| Family Size | 4.03 | 4.17 | 3.88 | 0.29 | 1.64 |
| Dependency Ration | 1.25 | 1.40 | 1.08 | 0.32 | 2.63^***^ |
| Average Family Age | 30.28 | 30.52 | 30.02 | 0.49 | 0.41 |

423 Note: *** means significant at 1%
424 424
425 **Dependency Ration:** The dependency ratio shows the ratio of economically active persons
426 compared to economically dependent household members. Economically active members of
427 households, whose age is from 14 to 64 were assumed to be the principal productive force
428 and sources of income for the household. Household members whose age was between 0-14
429 and above 64 were considered as economically inactive and dependent members of the
430 household. Similarly, the dependency ratio for the members of the sampled households
431 estimated to be 1.25 and 1.08 of the PW-UPSNP beneficiaries and non-UPSNP beneficiaries
432 respectively. Whereas, the average difference between treated and non-treated group are
433 around 0.32. Therefore, the result indicates that treated groups are the higher dependency
434 than control groups. Thus, the higher dependency contributes to food insecurity and the
435 probability level of participation significantly 1 percent (Table 3 above).
436 **Household Family Size:** It refers to the total number of the household’s member. Family
437 size is one of the major socioeconomic variables, which as a significant influence on the
438 amount of household consumption. As the number of the family increases, given the fixed
439 resources, the food share goes to the individual in that family declines which maximizes the
440 probability of being food insecure. As presented in Table 3, the average family size of the
441 combined samples was around 4. The average family size of beneficiary and non-beneficiary
442 households was 4 and 3 respectively. As a result, beneficiary households had greater family
443 size compared to non-beneficiary households (Table 3 above).

### 444 3.3 Impact of Productive Safety net Program on Community and

445 **Home asset values**
446 Different types of sustainable productive activities were being undertaken through public
447 works component of PSNP to build community assets. These activities largely focused on the
448 households’ area of community asset like maintenance of roads, maintenance and
449 construction of schools, maintenance and construction of latrine and cleaning surrounding
450 environment. Whereas, home asset (consumer and durable assets like radio, wrist watch, bed,
451 mobile phone, Tape recorder, Television and housing condition) were included. Then, the
452 result of the study revealed that in the households’ area of community asset and home asset
453 value calculated as the beneficiary worked and contribute as estimated at the values have
454 345.6 and 711.6 Ethiopian Birr respectively. Whereas, non-beneficiary households of
455 community asset and home asset value contribute to have 291.1 and 254.6 Ethiopian Birr
456 respectively. Therefore, the beneficiary households had greater amount of community and
457 home asset building tendency than non-beneficiary households (Table 4). According to this
458 finding, the mean difference in community asset and home asset value contribute between the
459 beneficiary and non-beneficiary households were found to be positive and statistically
460 significant at 1% of probability level. This implies that, the PSNP beneficiary households
461 possess community asset and home asset value that have contribute to greater value (quality)
462 as compared to the assets possessed by the non-PSNP beneficiary households.
463 **Table 4. Mean Comparisons for Impact Indicators Under Asset Accumulation.**

| Type of the asset | All Sample | UPSNP-  beneficiary | UPSNP non-  beneficiary | Mean  Difference | T-value |
| --- | --- | --- | --- | --- | --- |
| Current home asset value | 496.05 | 711.63 | 254.59 | 457.04 | 6.99*** |
| Community asset value | 319.95 | 345.66 | 291.15 | 54.51 | 2.64*** |

464 Where *** means significant at 1% level of significant

### 465 3.4 Impact of Productive Safety Net Program on Consumption

466 Consumption expenditure was used as impact indicator while evaluating impact of the
467 UPSNP, and it was computed as per adult equivalent consumption expenditure. The overall
468 households mean real consumption expenditure per adult equivalent (AE) for the sample
469 households was 257.63 Ethiopian birr. The mean consumption expenditure for PSNP
470 beneficiaries and non-beneficiary groups was 295.21 and 215.54 Ethiopian Birr respectively.
471 The mean difference test of consumption expenditure for the two groups was 79.68 Ethiopian
472 Birr. It implies that statistically significant at 1% probability level. Whereas the overall
473 households mean real food consumption and non-food consumption expenditure per adult
474 equivalent (AE) for the sample households was 209.95 and 47.68 Ethiopian Birr respectively
475 (Table 5).
476 **Table 5. The Impact of UPSNP on Households Food Consumptions Per Adult Equivalent.**

| Variables | All Samples | Public work beneficiary | Non-PSNP  beneficiary | Mean Difference | T-value |
| --- | --- | --- | --- | --- | --- |
| Consumption expenditure per adult  equivalent household | 257.63 | 295.21 | 215.54 | 79.68 | 3.88*** |
| Food consumption expenditure per  adult equivalent household | 209.95 | 237.72 | 178.86 | 58.86 | 3.04*** |
| Non- food consumption expenditure per adult equivalent  household | 47.68 | 57.49 | 36.68 | 20.81 | 5.83*** |

477 Note: *** means significant at 1% level of significant
478 478
479 The mean food consumption expenditure for PSNP beneficiary and non-beneficiary groups
480 was also 237.72 and 178.86 Ethiopian Birr respectively. The mean difference test of
481 consumption expenditure for the two groups was 58.86 Ethiopian Birr. It implies that
482 statistically significant at 1% probability level. Moreover, the mean non-food consumption
483 expenditure for PSNP beneficiary and non-beneficiary groups were 57.49 and 36.68
484 Ethiopian Birr respectively. Therefore, the result of the study showed that the PSNP
485 beneficiary households were found to be positive and statistically significant at 1% over the
486 intervention period. Besides, the	overall	consumption	per	adult	equivalent	for the
487 participants’ increased when compared to non-participants over the intervention period. This
488 result was supported by the evidences that taken from the key informants at the time of
489 interview. One of the key informants said that:
490 *“The beneficiary households have better money to pay for food items, medical cost and school fee with the help*
491 *of PW-UPSNP. Before they included in PSNP, some of the beneficiary households were eating once a day but*
492 *some others were eating twice in a day. After they were included in the PW-UPSNP, the majority of the*
493 *beneficiary households are eating three times in a day”.*

### 494 3.5 Econometric Results

495 The study used propensity score matching to evaluate the effects of PSNP on asset
496 accumulation and consumption expenditure among urban households. The propensity score
497 was estimated using a logistic model based on observable characteristics to determine the
498 likelihood of households participating in the program. Beneficiary and non-beneficiary
499 households were then matched based on their propensity scores, using a binary outcome
500 variable (1 for PW-PSNP beneficiary households and 0 for eligible non-PSNP beneficiary
501 households) in the logit model.

##### 502 3.5.1 Factors of Participation in Productive Safety Net Program

503 The researchers have incorporated relevant variables to explain the dependent variable and
504 conducted a logit model. They found that the gender, age of the household head, family size,
505 and off-farm income activities significantly affected program placement during selection.
506 The findings show that various factors significantly affected participation in PW-PSNP.
507 Employment on wage, family size, number of adult females, age of household head, and
508 employment on own business before UPSNP intervention all had positive and significant
509 influences on PW-PSNP participation, with varying magnitudes of impact.
510 Households with larger family sizes are more likely to participate in the program
511 compared to those with smaller family sizes. This is because larger families often have higher
512 food demand and a higher chance of food insecurity due to limited resources. The study
513 found family size to be statistically significant at a 5% level of significance. This result is
514 consistent with the studies conducted in Kenya and other rural parts of north Ethiopia by
515 [28,29] found that households with a large family size were more like to be food secure as
516 compared to those with a small family size. The reason may be as the household size
517 becomes larger, the number of economically active members may also increase, which leads
518 to increase their participation in various income-generating activities that could boost their
519 food security than for households with lower family size. Additionally, male-headed
520 households were more likely to be program participants compared to female-headed
521 households, likely due to females being active in non-farm activities generating off-farm
522 income. This result was also statistically significant at a 5% level of significance.
523 Household head age is the other variable which is found to be negatively significant at 1%
524 level of significant and related with probability of participation in PW-PSNP. This implies
525 that as increase in age of household heads, decreases their participation in the program. This
526 is due to the reason that, the age goes beyond certain periods; the person may become
527 physically weak and food insecure. On the other hand, variables like sex of household head,
528 food gap, accesses to extension services and access to remittance and number of adult males
529 are found to be statistically insignificant (Table 6).
530 **Table 6. 2Logit Model Estimates for Household’s Participation In UPSNP.**

| **Explanatory Variables** | **Coefficient** | **Roust Std. Err.** | **P- Value** |
| --- | --- | --- | --- |
| Sex of household head | -.647086 | .4778888 | 0.176 |
| Age of household head | -.3801537 | .1072199 | 0.000*** |
| Food gap before the implementation of the program | 1.521904 | 1.177113 | 0.196 |
| Employment on wage before the implementation of the  program | .8004876 | .4212125 | 0.057* |
| Employment on own business before the implementation of  the program | 1.241627 | .456983 | 0.007* |
| Accesses to extension services | .4487181 | .3334611 | 0.178 |
| Access to credit before the implementation of the program | -.2792303 | .4296875 | 0.516 |
| Access to remittance before the implementation of the  program | -.3621517 | .4033549 | 0.369 |
| Education of household head | .3446515 | .3264757 | 0.291 |
| Number of adult males | -.1649666 | .2454619 | 0.502 |
| Number of adult females | -.5768482 | .2447218 | 0.018** |
| Family size | .4680607 | .1911481 | 0.014** |
| Constant | 6.996062 | 3.094728 | 0.024 |

531 **Note**: *, **, *** means significant at 10%, 5% and 1% respectively
532 **3.5.2 Impact of UPSNP on Household Asset Accumulation**
533 In this study, household home assets represent the sum of household goods like home, pot,
534 oven, bed, box and barrel and consumer durables like mobile phone, watch, tape recorder and
535 Radio and Television etc. After converting both the household goods and the consumer
536 durable of the entire households in to their respective market value, the two categories have
537 been added together to form the household home assets.
538 The impact of UPSNP on household’s home asset were found to be positive with the
539 maximum and the minimum ATT value calculated to be 494 Ethiopian Birr (based on NN
540 matching) and 441 Ethiopian Birr (based on Kernel matching) (Table 7) respectively.
541 **Table 37. ATT Estimated Results on the Households Asset Accumulation.**

| Outcome variables | Matching estimators | Public work UPSNP  beneficiary | Eligible non-PSNP beneficiary | Average treatment effect on the treated (ATT) | T-statistics |
| --- | --- | --- | --- | --- | --- |
| Current value of home asset | Nearest neighbor | 62 | 23 | 494.40 | 6.47*** |
|  | Stratification | 62 | 49 | 455.37 | 7.24*** |
|  | Kernel | 62 | 49 | 441.57 | 6.01*** |
| Community Asset | Nearest neighbor | 62 | 23 | 82.88 | 2.13** |
|  | Stratification | 62 | 49 | 54.44 | 2.61*** |
|  | Kernel | 62 | 49 | 59.72 | 2.49*** |

542 **Note:** **, *** means significant at 5% and 1% respectively
543 Statistically, this result is found to be significant at one percent for all matching estimators
544 used in this study, i.e., based on Kernel, Stratification and NN matching. The implication of
545 this result is, because of the introduction of the program, the value of non-productive/home
546 asset at the hands of the PW-PSNP beneficiary households is higher than the value of the
547 non-productive asset at the hands of the non-beneficiary households by the amount of at
548 maximum 494 Ethiopian Birr and at minimum 441 Ethiopian Birr. And this result confirms
549 the fact that the program is successful in avoiding the depletion in the non-productive asset of
550 the beneficiary households.
551 According to this finding, the program has a positive and significant impact on the household
552 asset at 1% level of significance. This can be also stated as PW-PSNP beneficiary households
553 have more asset value than the eligible non-beneficiary households. Generally, the above
554 positive value of ATT implies that, because of the program intervention household’s home
555 assets have been protected in the study area.
556 During the field survey, the participants of the PW-PSNP program made efforts to enhance
557 community assets, as evidenced by their contributions to activities such as road maintenance,
558 improving the greenery of the environment, and erecting fences around schools, health posts,
559 and ponds. The result of this study shows that the current value of community assets at the
560 hands of the PSNP beneficiary households is higher than the current value of community
561 assets at the hand of the non-PSNP beneficiary households.
562 The mean difference in the current value of community assets between the PW-PSNP
563 beneficiary and eligible non-beneficiary households were found to be positive and significant
564 with the minimum and maximum ATT value of contribute as calculated 54 Ethiopian Birr
565 (based on Stratification matching) and 82 Ethiopian Birr (based on the NN matching)
566 respectively. Statistically, the calculated ATT for the treated and control group is found to be
567 significant at 1% based on Kernel and Stratification matching while 5% with nearest
568 neighbor matching estimators used in the study.
569 According to the result of this study on average the community assets of the PW-PSNP
570 beneficiary households contribute to the value at maximum 82 Ethiopian Birr (based on NN
571 matching) and to the minimum 54 Ethiopian Birr (based on Stratification matching) more as
572 compared to the community assets of the eligible non- beneficiary households.
573 In general, because of their participation in the program the PW-PSNP beneficiary
574 households have managed to protect their community assets from possible deterioration.
575 Thus, the program enables the PSNP beneficiary households to preserve their community
576 assets from depletion. The result coincides with [25], who assessed the impact of Ethiopia’s
577 PSNP using the method of propensity score matching method based on various impact
578 indicators, also found that the mean difference in community assets between PW-PSNP and
579 eligible non-PSNP households are positive and significant at one percent. Therefore, the
580 result of the study showed that participation in PW-PSNP was found to be helpful to the
581 participants in improving their community assets. This result is also coinciding with the
582 finding of Yibrah [30], who studied the impact of participating in the Productive Safety Net
583 Program on Consumption and Asset holding.

#### 584 3.5.3 Food Security Status of Sample Households

585 Data on food consumption was collected from 112 households for this study, aiming to
586 capture the diversity and frequency of various foods consumed within a 24-hour period. The
587 study utilized a household food consumption score, which is a continuous measure and can
588 be divided into three levels. The results indicated that 45 households (72.5%) of PW-UPSNP
589 beneficiaries had acceptable food consumption, 11 households (17.7%) had borderline
590 consumption, and 6 households (9.6%) were considered poor in terms of their food
591 consumption score. On the one hand, among eligible non-UPSNP beneficiary households,
592 38% had acceptable food consumption, 36% had borderline consumption, and 26% had poor
593 consumption. The households with poor consumption are considered food insecure, while
594 those with borderline consumption are moderately food insecure, and the households with
595 adequate food consumption are food secure. On the other hand, the mean food consumption
596 score for PW-UPSNP beneficiary households was 1.53, whereas for eligible non-PSNP
597 beneficiary households, it was 1.23. Consequently, there is a significant difference in the
598 mean food consumption score between PW-UPSNP beneficiary households and eligible non-
599 UPSNP beneficiary households, which amounts to 1.38.
600 The outcome suggests that households benefiting from PW-UPSNP are more food secure
601 compared to those eligible for PSNP but not receiving PW-UPSNP. The chi-square test
602 confirms a significant difference in food security status between the two groups at a 1% level
603 of significance (Table 8).
604 **Table 48. Households Food Security Status.**

| Food consumption score | Public work UPSNP beneficiary | | Eligible-non-Public work-UPSNP beneficiary | | Total | | X^2^-value |
| --- | --- | --- | --- | --- | --- | --- | --- |
|  | Frequency | % | Frequency | % | Frequency | % |  |
| Acceptable food consumption (>42) | 45 | 72.5 | 19 | 38 | 64 | 57 |  |
| Borderline food consumption (28.5-42) | 11 | 17.7 | 18 | 36 | 29 | 26 |  |
| Poor food consumption (≤28) | 6 | 9.6 | 13 | 26 | 19 | 17 |  |
| Total | 62 | 100 | 50 | 100 | 112 | 100 |  |
| Mean | 1.53 |  | 1.23 | | 1.38 |  | 15.06 *** |

605 **Note**: *** Significant at 1% level
606 606

#### 607 3.5.4 Food Consumption Expenditure Per Adult Equivalent

608 Food consumption expenditure behavior can be used to assess household food security. The
609 average food consumption expenditure for households benefiting from PW-UPSNP was
610 237.72 Ethiopian Birr, while for non-beneficiary households, it was 178.86 Ethiopian Birr.
611 The statistically significant difference of 58.86 Ethiopian Birr between the two groups was
612 observed at a 1% probability level (Table 9).
613 **Table 9. 5Mean Comparisons on the Impact of UPSNP On Households Food Consumptions Rate Per**
614 **Adult Equivalent.**

| Variables | All sample  mean | Public work UPSNP  beneficiary | Eligible-non- UPSNP  beneficiary | Mean Difference | T-value |
| --- | --- | --- | --- | --- | --- |
| Food consumption expenditure per  adult equivalent household | 209.95 | 237.72 | 178.86 | 58.86 | 3.04*** |

615 **Note:** *** means significant at 1% level of significant
616 616
617 Moreover, this result indicates that the PW-UPSNP on beneficiary households food
618 consumption had a positive with the maximum and the minimum ATT value calculated to be
619 59 Ethiopian Birr (based on stratification matching) and 45 Ethiopian Birr (based on NN
620 matching) respectively (Table 10).
621 **Table6 10. ATT Estimated Result of The Impact of UPSNP On Household Food Consumption**
622 **Expenditure.**

| Outcome variables | Matching estimators | PW UPSNP  beneficiary | eligible non- UPSNP  beneficiary | Average treatment effect on treated | t-statistics |
| --- | --- | --- | --- | --- | --- |
| Food consumption expenditure per adult equivalent | Nearest  neighbor | 62 | 23 | 45.6 | 1.51 |
|  | Stratification | 62 | 49 | 59.63 | 3.19^***^ |
|  | Kernel | 62 | 49 | 54.07 | 2.71^***^ |

623 **Note:** *** Significant at 1% level
624 624

# 625 Conclusions

626 Chronic poverty and lack of food are major global problems, particularly in developing
627 regions. In Ethiopia, around 8.5 million people, constituting 10% of the population, suffer
628 from chronic food insecurity, leading to asset selling and consumption restrictions. A study
629 shows that factors like household head's age, family size, and off-farm income activities
630 influence participation in the PW-UPSNP program. Beneficiary households have more assets
631 than eligible non-beneficiary households. The PW-UPSNP program significantly increases
632 food	consumption,	non-food	consumption,	and	total	consumption	expenditure	for
633 beneficiaries compared to non-beneficiaries.
634 Econometric analysis reveals positive effects of the PW-UPSNP program on community
635 and home asset accumulation. More PW-UPSNP beneficiaries are food secure compared to
636 non-beneficiaries. The mean food consumption expenditure is significantly higher for PW-
637 UPSNP beneficiaries. The program has a positive and statistically significant impact on food
638 consumption. The second phase of the PSNP program in Ethiopia ensures food security for
639 both chronically and temporarily food insecure households. It aims to assist over 7.5 million
640 people across eight regions through program transfers and productive activities.
641 Previous emergency food relief efforts were insufficient, leading to the implementation of
642 the PSNP program. It is the largest social protection program in Sub-Saharan Africa and
643 focuses on selected households over several years to achieve sustainable food security. Most
644 studies have focused on rural areas, but the overall conclusion is that the PW-UPSNP
645 program has a positive effect on consumption, asset accumulation, and food security. The
646 program successfully addresses chronic poverty and food insecurity in Ethiopia.

#### 647 Recommendation

648 The UPSNP program has proven to be an effective social protection intervention in
649 combating chronic poverty and food insecurity in urban areas of Ethiopia. Its positive impact
650 on asset accumulation, food consumption, and overall well-being of beneficiary households is
651 evident. Therefore, it is recommended to continue and expand the UPSNP program to reach
652 more vulnerable urban households and further improve their livelihoods. By doing so,
653 Ethiopia can make significant strides towards achieving sustainable development and
654 reducing poverty and food insecurity in urban areas.

##### 655 Acknowledgments

656 The authors would like to thank farmers, agricultural development agents, and local
657 administrators of the study area for their assistance during the field work.

##### 658 Declarations

659 The authors declare that they have no conflict of interests.

##### 660 Competing Interests

661 The authors have declared that no competing interests exist.

##### 662 Data Availability

663 Data used and/or analysed in the study are available from the corresponding author on
664 reasonable request.

##### 665 Funding

666 The author(s) received no specific funding for this work.

##### 667 Author Contributions

668 Conceptualization: Bezaye Endris, Alem-meta Assefa.
669 Data curation: Bezaye Endris, Alem-meta Assefa.
670 Formal analysis: Bezaye Endris, Alem-meta Assefa.
671 Investigation: Bezaye Endris, Alem-meta Assefa.
672 Methodology: Bezaye Endris, Alem-meta Assefa.
673 Project administration: Bezaye Endris, Alem-meta Assefa.
674 Resources: Bezaye Endris, Alem-meta Assefa.
675 Software: Bezaye Endris, Alem-meta Assefa.
676 Supervision: Bezaye Endris, Alem-meta Assefa.
677 Validation: Bezaye Endris, Alem-meta Assefa.
678 Visualization: Bezaye Endris, Alem-meta Assefa.
679 Writing – original draft: Bezaye Endris, Alem-meta Assefa.
680 Writing – review and editing: Bezaye Endris, Alem-meta Assefa.

| 681 | **REFERENCES** |
| --- | --- |
| 682 | 1. Fekadu N, Mberengwa (2009) Challenges of Productive Safety Net Program |
| 683 | Implementation at Local Level. The Cases of Kuyu Woreda, North Shewa Zone, |
| 684 | Oromia Region, Ethiopia. *Journal of Sustainable Development in Africa,* 11(1), 248- |
| 685 | 267. |
| 686 | 2. Samuel G (2010) Food aid and small householder agriculture in Ethiopia development |
| 687 | studies, March 20-22. |
| 688 | 3. World Bank (2012) Retrieved from; Washington, D.C: 2012. Managing risk, promoting |
| 689 | growth: Developing systems for social protection in Africa‐ ‐ the World Bank’s |
| 690 | Africa social protection strategy 2012-2022. [[Google Scholar](https://scholar.google.com/scholar?q=World%2BBank%2BManaging%2Brisk%2C%2Bpromoting%2Bgrowth%E2%80%AF%3A%2BDeveloping%2Bsystems%2Bfor%2Bsocial%2Bprotection%2Bin%2BAfrica%E2%80%90%E2%80%90the%2BWorld%2BBank%E2%80%99s%2BAfrica%2Bsocial%2Bprotection%2Bstrategy%2B2012-2022%2B2012%2BRetrieved%2Bfrom%2BWashington%2C%2BD.C%2B)] |
| 691 | 4. FAO, IFAD, UNICEF, WFP and WHO (2023) The State of Food Security and Nutrition |
| 692 | in the World 2023. Urbanization, agrifood systems transformation and healthy diets |
| 693 | across the rural–urban continuum. Rome, FAO. <https://doi.org/10.4060/cc3017en> |
| 694 | 5. Africa Center for Strategic Studies (2023) Unresolved Conflicts Continue to Drive |
| 695 | Africa’s Food Crisis. africacenter.org/spotlight/unresolved-conflicts-continue-to-drive- |
| 696 | africas-food-crisis/ |
| 697 | 6. Ministry of Agriculture and Rural Development (MOARD) (2010) Concept Note for |
| 698 | Establishment of a System of Productive Safety Net in Ethiopia. |
| 699 | 7. Berhane G, J Hoddinott, Kumar H, Alemayehu S, Michael T, Yisehac Y et al. (2013) |
| 700 | ‘Evaluation of Ethiopia’s Food Security Program: Documenting Progress in the |
| 701 | Implementation of the Productive Safety Nets Program and the Household Asset |
| 702 | Building Programm’. ESSP II–EDRI REPORT, Washington, D.C: International Food |
| 703 | Policy Research Institute. |
| 704 | 8. Federal Democratic Republic of Ethiopia (FDRE) (2006) Productive Safety Net Program: |
| 705 | Program Implementation Manual(rev). Ministry of Agriculture and Rural Development |
| 706 | (MoARD), Addis Ababa, Ethiopia. |
| 707 | 9. Federal Democratic Republic of Ethiopia (FDRE) (2001) Rural Development Policies, |
| 708 | Strategies and Instruments of the Government of the FDRE. |
| 709 | 10. Berhane G, J Hoddinott, Kumar N, Alemayehu S (2011) The impact of Ethiopia’s |
| 710 | Productive Safety Nets and Household Asset Building Programme:2006-2010. |
| 711 | Washington, D.C: International Food Policy Research Institute. |
| 712 | 11. Ministry of Urban Development and Housing UPSNP (2016) Urban Productive Safety |
| 713 | Net Program Implementation Manual, Version xx, Addis Ababa. |
| 714 | 12. Dessie City UPSNP Task Force Office (2019) Unpublished Report Document, Ethiopia. |
| 715 | 13. Andersson C, Alemu M, Stage J (2010) Impacts of the Productive Safety Net Program in |
| 716 | Ethiopia on livestock and tree holdings of rural households Environment for |
| 717 | Development Discussion Paper Series 09-05. |
| 718 | 14. Devereux S, Sabates-Wheeler R (2004) Transformative social protection. IDS Working |
| 719 | Paper, 232. Institute of Development Studies, Brighton, Sussex Bn1 9RE England. |
| 720 |  |
| 721 | 15. Kataru K (2011) Assessment of the Impact of Productive Safety Net Program on the |
| 722 | Lives of the Beneficiaries in Ethiopia: The Case of Dera Malo Woreda, Gamo Gofa |
| 723 | Zone, unpublished Master of Arts Degree in Public Management, Addis Ababa |
| 724 | University, Ethiopia. |

725 16. Berhane G, J Hoddinott, Kumar N, Alemayehu S (2011) The impact of Ethiopia’s 726	Productive Safety Nets and Household Asset Building Programme:2006-2010. 727	Washington, D.C: International Food Policy Research Institute.

728	17. Tadele M (2011) Impact of Productive Safety Net Program on the sustainable Land

729	Management Practice in Central Rift value. University of Haramaya, Ethiopia.

730 18. Andersson C, Alemu M, Jesper S (2011) Impacts of the Productive Safety Net Program in 731	Ethiopia on livestock and tree holdings of rural households. *Journal of Development* 732	*Economics*, 94 (2011) 119–126. doi:10.1016/j.jdeveco.2009.12.002.

733	19. Habitamu A (2011) Impact of Productive Safety Net Program on the Household Welfare 734	and labor supply in rural Ethiopia. Master’s Thesis, Addis Ababa University, Ethiopia. 735	20. Central	Statistics Authority (CSA) (2007) National	Population Statistics. Federal 736	Democratic Republic of Ethiopia, Central Statistical Authority, Addis Ababa, 737	Ethiopia.

738 21. Kombo DK, Tromp DL (2006) *Thesis Writing*: An Introduction, Paulines Publications
739 Africa, Nairobi.
740 22. Creswell JW (2009) *Research Design: Qualitative, Quantitative, and Mixed Methods*
741 *Approaches.* 3^rd^Edition, SAGE Publications, London, United Kingdom.
742 23. Kothari CR (2004) *Quantitative Techniques*. 2^nd^Edition, New Delhi: Vikas Publishing
743 House Pvt. Ltd. India.
744 24. Gujarati DN (2004) *Basic Econometrics*. 4^th^Edition, the McGraw−Hill Companies.
745 25. Gilligan DO, Hoddinott J, Alemayehu S (2008) *An analysis of Ethiopia’s Productive* 746	*Safety Net Program and its linkages*. International Food Policy Research Institute, 747	Washington, DC.

748	26. Rosenbaum PR, Rubin DB (1983) The Central Role of the Propensity Score in

749	Observational Studies for Causal Effects. *Biometrika*,70,41-55.

750	<http://dx.doi.org/10.1093/biomet/70.1.41>

751 27. Derso A, Bizuneh H, Keleb A, Ademas A, Adane M (2021) Food insecurity status and 752	determinants among Urban Productive Safety Net Program beneficiary households in 753	Addis	Ababa, Ethiopia. *PLOS	ONE,*16(9): e0256634.https://doi. 754	org/10.1371/journal.pone.0256634

755	28. Alemseged G, Hailu B (2018) Determinants of rural household food insecurity in 756	Northern Ethiopia: An empirical study. *Sustainable Development Africa*, 2018; 757	20(1):32–63.

758	29. Scho L, Wekesah F, Mohamed S (2014) Vulnerability to food insecurity in urban slums:

759	Experiences from Nairobi, Kenya. *Journal of Urban Health*, 2014; 91(6):1098–113.

760	30. Yibrah H (2010) Impact of Productive Safety Net Program on the Rural Households asset

761	Protection and Consumption. Unpublished MA Thesis, Mekelle University, Ethiopia.

762

Response to Reviewers

##### Response to Reviewers Minor comments

1. In the abstract, the authors should remove italics from this section.

**Response**: We did it.

2. In the introduction, line 53, the name of the author should be mention before the reference number.

**Response:** Mentioned. Samuel [2]…

3. Lines 59 &60, approximately 850 million people worldwide faced food insecurity, with a majority residing in developing regions. According to the state of food insecurity (SOFI) 2023 report how many people are food insecure worldwide? What is the share of Africa’s continent?

**Response:** The answer is given as follow:

The relative lack of change in hunger at the global level from 2021 to 2022 hides substantial differences at the regional level. Progress was made towards reducing hunger in most subregions in Asia and in Latin America, but hunger is still on the rise in Western Asia, the Caribbean and all subregions of Africa. The proportion of the population facing hunger is much larger in Africa compared with the other regions of the World – nearly 20 percent compared with 8.5 percent in Asia, 6.5 percent in Latin America and the Caribbean, and 7.0 percent in Oceania [4].

4- Lines 60 &61, In Africa, around 200 million people, accounting for 27.4 percent of the population, experienced food insecurity between the 1970s and 1990s. what is the current prevalence for Africa in recent time?2020-2023?

##### Response:

- An estimated 149 million Africans are facing acute food insecurity—an increase of 12 million people from a year ago. This equates to a risk category of 3 or higher (Crisis, Emergency, and Catastrophe) on the Integrated Food Security Phase Classification (IPC) scale of 1 to 5. Some 122 million of those facing acute food insecurity are in countries experiencing conflict—82 percent of the total—accentuating that conflict is the primary driver of acute food insecurity in Africa [5]. Four of the top 10 countries facing the most acute food insecurity are in East Africa—Sudan, Ethiopia, South Sudan, and Somalia. An estimated 20.1 million people are facing food insecurity in Ethiopia, and 15.1 million require emergency food assistance, following a 2-year war in Tigray, conflicts in other parts of the country, as well as a severe drought that struck the Horn of Africa. The World Food Program and other donors suspended food aid in Ethiopia for 4 months following the discovery of a large-scale diversion within the country [5].

5- Lines 61 & 62, Ethiopia, in particular, had about 8.5 million chronically food insecure individuals, constituting 10 percent of its population. Is this the most recent data available?

##### Response:

- An estimated 20.1 million people are facing food insecurity in Ethiopia, and 15.1 million require emergency food assistance, following a 2-year war in Tigray, conflicts in other parts of the country, as well as a severe drought that struck the Horn of Africa. The World Food Program and other donors suspended food aid in Ethiopia for 4 months following the discovery of a large-scale diversion within the country [5].

6- Lines 65 &66 conducted a study indicating that the second phase of PSNP plays a crucial role in ensuring food security for both chronically and temporarily food insecure households in Ethiopia. Meaning of PSNP? State the full meaning at first mention before abbreviating.

**Response:** corrected.

The Productive Safety Net Program (PSNP)

7- Lines 67 &68 districts across eight regions by providing program .transfers and supporting productive activities until December 2014. What was its success rate and what is it fails out as compared with the initial emergency relief appeal method?

##### Response:

Before implementing PSNP, the Ethiopian 69 government relied on unpredictable annual appeals for emergency food relief, which saved lives but failed to 70 address the increasing number of food insecure individuals [6]. Based on the information provided, the study conducted by Berhane et al. [7] indicates that the second phase of the Productive Safety Net Program (PSNP) in Ethiopia has several successes when compared with the initial emergency relief appeal method. These successes include:

**Enhanced food security**: The study suggests that the PSNP plays a crucial role in ensuring food security for both chronically and temporarily food insecure households. By providing program transfers and supporting productive activities, the PSNP aims to assist over 7.5 million people in 318 districts across eight regions. This targeted approach is likely more effective in addressing the needs of food insecure individuals compared to the unpredictable annual appeals for emergency food relief.

**Long-term planning and stability:** Unlike the initial emergency relief appeal method, which relied on unpredictable annual appeals for food aid, the PSNP operates on a set timeline until December 2014. This longer-term planning allows for more stability and predictability in addressing food insecurity issues.

**Diversification of support:** The PSNP not only provides program transfers but also supports productive activities. This indicates that the program aims to go beyond providing immediate relief by helping households develop sustainable livelihoods and improve their resilience to food insecurity.

However, without further information on the specific failures of the initial emergency relief appeal method, it is challenging to make a direct comparison. It is important to note that the PSNP is a complex program with its own limitations and challenges. Further analysis and evaluation would be needed to understand the full extent of its successes and failures in comparison to the initial emergency relief appeal method.

8- Line 82, million urban poor living in 972 cities and towns. Urban poor what? dwellers/resident?

##### Response:

The Ethiopian government has been working on a program called the Urban Productive Safety Net Program (UPSNP) that will span a 10-year period. This program is part of the Urban Food Security and Job Creation Strategy, which was officially endorsed on May 8, 2015. The purpose of the UPSNP is to provide assistance to more than 4.7 million impoverished individuals living in 972 cities and towns across the country.

9- Lines 86-88, beneficiaries, In the first phase, 604,000 the poorest 12 percent and about 55 percent of people living below the poverty line in these 11 cities Urban Productive Safety Net Project [10]. Incomplete statement. What happened to 604,000?

**Response:** Corrected as follow:

In the initial stage of the UPSNP, approximately 604,000 individuals who are among the most impoverished 12 percent and around 55 percent of the population residing below the poverty threshold in these 11 cities were the recipients of the program's benefits.

10- Line 93, Having these in mind the researcher was try to evaluate the impacts of urban productive safety net program on urban households’ asset accumulation , consumption and food security status in Dessie City. Delete the word was.

**Response:** Corrected as follow:

Having these in mind, the researchers tried to evaluate the impacts of urban productive safety net program on urban households’ asset accumulation, consumption and food security status in Dessie City.

11- Line 98 Chronically, food insecurity has been one of the most problems of urban poverty, particularly in drought-prone. Please rephrase for clarity.

**Response:** Rephrased as follow:

Historically, urban poverty has consistently faced the significant challenge of food insecurity, especially in drought-prone areas of Ethiopia.

12- Line 100, High proportion of food insecure households, which have been receiving emergency food aids. Remove the word which.

**Response:** Corrected as follow:

High proportion of food insecure households have been receiving emergency food aids.

13- Line 101, Although the emergency assistance was substantial and saved many lives, it was uncertainty of the food aid, …. Do you mean the uncertainty/or inconsistency in availability of food aid?

**Response:** It is the uncertainty of the food aid and corrected as follow:

Although the emergency assistance was substantial and saved many lives, there was uncertainty of the food aid, poor timing of provision and insufficient quantity of aid for the individual’s beneficiary was among the main defects of food aids in the past and creating dependency syndrome on the recipients [13].

14- Lines 107-109, aim of preventing asset depletion at household level while creating assets at the community level (NO REFERENCE).

**Response:** The reference is included (Look at the following statement):

Recognizing this, the government of Ethiopia changed the emergency food-based assistance to multi- year 108 support of PSNP in 2005 that provides cash and food transfers to chronically food insecure households with the aim of preventing asset depletion at household level while creating assets at the community level Devereux and Sabates (2004).

15- Lines 112-113, Some findings state that, PSNP has able to smooth the consumption and protects household’s assets (GRAMMER CHECK for clarity).

**Response:** Checked.

According to Kataru’s [13] research finding, it has been determined that the Productive Safety Net Program (PSNP) is capable of effectively mitigating consumption fluctuations and safeguarding household assets.

16- Line 113, [14] indicated that beneficiary households have managed to 114 significantly improve their food security status by 1.05 months. Mention the authors NAME BEFORE the reference number.

**Response:** Corrected as follow:

Berhane et al. [16] indicated that beneficiary households have managed to significantly improve their food security status by 1.05 months.

17- Lines 114-115, In contrast to these, what proved is that there is no significant difference between, the consumption as well as assets of PSNP participants and non-participants. Please clarify this statement for better understanding.

**Response:** Clarified as follow:

On the contrary, what has been demonstrated is that there is no noteworthy distinction between the consumption and assets of individuals involved in the PSNP program and those who are not [17].

18- Lines 116-118, Moreover, [17], stated that, participation in PSNP reduces the asset size of the participants, due to the dependency behavior of beneficiaries which discourage them to put efforts on their agricultural activities. Do mention the author before reference.

##### Response:

Furthermore, Habitamu [19], stated that, participation in PSNP reduces the asset size of the participants, due to the dependency behavior of beneficiaries which discourage them to put efforts on their agricultural activities.

19- Line 112, levels have brought inconsistent results and far from conclusive,(This statement is not clear).

**Response:** corrected as follow:

The previous ideas indicated that various researchers have examined the effects of the program at both the national and local levels, but their findings have been inconsistent and far from definitive. Consequently, this study was conducted to address this gap.

20- Line 136, THE RESEARCH DESIGN AND SAMPLING. Put this in sentence case.

##### Response: 2.2 The Research Design and Sampling

21- Lines 137-139, Because of survey design helped identify patterns of behavior, activities, people‘s attitude, opinions, habits and any of the variety of social problems that influence the explicability and scaling-up efforts of the program across various contexts. Recast as survey design helps to identify….

**Response:** Reorganized as follow:

The survey design helps to identify patterns of behavior, activities, people’s attitude, opinions, habits and any of the variety of social problems that influence the explicability and scaling-up efforts of the program across various contexts

22- Lines 139-141, Descriptive survey design help to identify the issues had been occurred and could be predetermine certain preventive measures could be adopted. Paraphrase this sentence for clarity and better understanding

**Response:** Paraphrased as follow:

The utilization of a descriptive survey design aids in identifying past occurrences and enables the pre- determination of specific preventive measures that can be implemented.

23- Line 144, The quantitative data were analyzed and interpreted in the numerical data through appropriate. Delete – the words… in the numerical data

##### Response:

The quantitative data were analyzed and interpreted through appropriate statistical tools and cross tabulations were made to facilitate meaningful analysis and interpretations of the research findings.

24- Line 154, stated that target population as all members of a real set of subjects to which a researcher wishes to……. Replace as by are

##### Response:

Creswell [22], stated that all target populations are member of a real set of subjects to which a researcher wishes to generalize the results of the study.

25- Lines 155-156, … The study was targeted each selected *Kebele*, the name of the entire list of household heads were obtained from *Kebele* administration office. They should be corrected grammatically.

**Response:** corrected as follow:

The study specifically focused on each chosen Kebele, and the researcher obtained the complete roster of household heads from the Kebele administration office.

26- Line 157-158, Then on the bases of *Kebele* document there are 338 PWPSNP beneficiary households and 272 eligible non-beneficiary households in Robit *Kebele.* Full meaning

##### Response:

Then on the bases of *Kebele* document there are 338 Public Work-Productive Safety Net Program (PW- PSNP) beneficiary households and 272 eligible non-beneficiary households in Robit *Kebele*.

27- Line 160, beneficiary household heads were used to as sample frames included for the survey (The statement needs to be improved upon grammatically).

##### Response:

… beneficiary household heads were used as sample frames included for the survey.

28- Line 175, In addition to this, 6 key informant interviewees were conducted with purposively. You mean interview?

**Response:** Yes, I mean interview. Accordingly, it has corrected as follow:

Additionally, interviews were conducted with six key informants, specifically selected Kebele Development Agents, Kebele Administrators, and the head of the UPSNP Task Force.

29- Lines 175-176, ). In addition to this, 6 key informant interviewees were conducted with purposively selected *Kebele* Development Agents, *Kebele* Administrators and head of UPSNP Task Force. You mean interview? Please rephrase for clarity.

**Response:** Yes, I mean interview. Accordingly, it has rephrased as follow:

In addition to this, interviews were conducted with six key informants, specifically selected *Kebele*

Development Agents, *Kebele* Administrators, and the head of the UPSNP Task Force members.

30- Line 178, The study was employed both primary and secondary data sources. Delete-was

**Response:** The word was is deleted.

The study employed both primary and secondary data sources.

31- Line184, The study was employed both quantitative and qualitative data analysis techniques. Delete-was

**Response:** The word was is deleted.

The study employed both quantitative and qualitative data analysis techniques.

32- Lines 186 &187, Similarly, qualitative data was gathered through open-ended questionnaires and interview and analyzed through textually to supplement the questionnaire survey. Delete-through

**Response:** The word through is deleted.

Similarly, qualitative data was gathered through open-ended questionnaires and interview and analyzed textually to supplement the questionnaire survey.

33- Lines 189 &190, To understand the distribution of the covariates across the two groups, the quantitative data were analyzed the 190 independent variables through simple descriptive analysis, such as frequencies, and percentages were done to summarized in tables, interpret and conclude the results. please rephrase.

**Response:** Paraphrased as follow:

To assess the distribution of the covariates between the two groups, the quantitative data underwent analysis using descriptive techniques.

34- Line 190, and percentages were done to …..

**Response:** Paraphrased and corrected as follow:

This involves examining the independent variables through methods such as calculating frequencies and percentages, which were then summarized in tables to interpret and draw conclusions from the results.

35- Lines 193 &194, In addition, to the simple descriptive analysis, to estimate the impact of participation in the urban Productive Safety Net program. Delete-in addition to---

**Response:** The phrase in addition to is deleted.

36- Line 198, According to [22], in estimating logit model for the dichotomous (binary) logistic regressions will be used to….

**Response:** Will be is replaced by was.

According to Gujarati [24], in estimating logit model for the dichotomous (binary) logistic regressions was used to achieve this objective, particularly; participation which takes a value of 1 if the household participated in a program and 0 otherwise.

37- Line 249, …by classifying the households’ consumption in to two main categories,

**Response:** It is rewritten as into

… by classifying the households’ consumption into two main categories,

38- Line 254, .. the field survey based on the respondents’ their consumption rate in terms of Ethiopian birr.

**Response:** The word their is deleted.

… the field survey based on the respondents’ consumption rate in terms of Ethiopian Birr.

39- Results and Discussion section (pages 10- 17) should be well written with appropriate comparison with relevant previous research output/published articles and must be well referenced. That is this section should not just be result presentations alone without being adequately discussed.

**Response: we tried to rewrite it with** appropriate comparison with relevant previous studies. For example, 1. a study conducted by Derso et al. [27] supports the result of credit experience variable.

… A study done by Derso et al. [27] supported this result. It indicated that three in four households were experiencing food insecurity and that an appropriate multi-dimensional approach such as the opening of small business enterprises through credit service support may help to diversify income sources and to reduce the dependency ratio.

2. a study done by Alemseged G, Hailu B (2018) as well as by Scho L, Wekesah F, Mohamed S (2014) supported the result of the variable family size … This result is consistent with the studies conducted in Kenya and other rural parts of north Ethiopia by [28,29] found that households with a large family size were more like to be food secure as compared to those with a small family size. The reason may be as the household size becomes larger, the number of economically active

members may also increase, which leads to increase their participation in various income- generating activities that could boost their food security than for households with lower family size.

40- Line 474, CONCLUSION. Can you make it precise?

**Response:** Yes, we did it.

Chronic poverty and lack of food are major global problems, particularly in developing regions. In Ethiopia, around 8.5 million people, constituting 10% of the population, suffer from chronic food insecurity, leading to asset selling and consumption restrictions. A study shows that factors like household head's age, family size, and off-farm income activities influence participation in the PW-UPSNP program. Beneficiary households have more assets than eligible non-beneficiary households. The PW-UPSNP program significantly increases food consumption, non-food consumption, and total consumption expenditure for beneficiaries compared to non-beneficiaries.

Econometric analysis reveals positive effects of the PW-UPSNP program on community and home asset accumulation. More PW-UPSNP beneficiaries are food secure compared to non-beneficiaries. The mean food consumption expenditure is significantly higher for PW- UPSNP beneficiaries. The program has a positive and statistically significant impact on food consumption. The second phase of the PSNP program in Ethiopia ensures food security for both chronically and temporarily food insecure households. It aims to assist over 7.5 million people across eight regions through program transfers and productive activities.

Previous emergency food relief efforts were insufficient, leading to the implementation of the PSNP program. It is the largest social protection program in Sub-Saharan Africa and focuses on selected households over several years to achieve sustainable food security. Most studies have focused on rural

areas, but the overall conclusion is that the PW-UPSNP program has a positive effect on consumption, asset accumulation, and food security. The program successfully addresses chronic poverty and food insecurity in Ethiopia.

41- Line 534, (pages 20-21) **REFERENCES.** Most of references are outdated. Kindly work on all

**Response:** We tried to up-date some of them. For example, the following references are included. World Bank (2012) Retrieved from; Washington, D.C: 2012. Managing risk, promoting growth:

Developing systems for social protection in Africa‐‐the World Bank’s Africa social protection strategy 2012-2022. [[Google Scholar](https://scholar.google.com/scholar?q=World%2BBank%2BManaging%2Brisk%2C%2Bpromoting%2Bgrowth%E2%80%AF%3A%2BDeveloping%2Bsystems%2Bfor%2Bsocial%2Bprotection%2Bin%2BAfrica%E2%80%90%E2%80%90the%2BWorld%2BBank%E2%80%99s%2BAfrica%2Bsocial%2Bprotection%2Bstrategy%2B2012-2022%2B2012%2BRetrieved%2Bfrom%2BWashington%2C%2BD.C%2B)]

FAO, IFAD, UNICEF, WFP and WHO (2023) The State of Food Security and Nutrition in the World 2023. Urbanization, agrifood systems transformation and healthy diets across the rural–urban continuum. Rome, FAO. <https://doi.org/10.4060/cc3017en>

5. Africa Center for Strategic Studies (2023) Unresolved Conflicts Continue to Drive Africa’s Food Crisis. africacenter.org/spotlight/unresolved-conflicts-continue-to-drive-africas-food-crisis/

. Berhane G, J Hoddinott, Kumar H, Alemayehu S, Michael T, Yisehac Y et al. (2013) ‘Evaluation of Ethiopia’s Food Security Program: Documenting Progress in the Implementation of the Productive Safety Nets Program and the Household Asset Building Programm’. ESSP II–EDRI REPORT, Washington, D.C: International Food Policy Research Institute.

Devereux S, Sabates-Wheeler R (2004) Transformative social protection. IDS Working Paper, 232.

Institute of Development Studies, Brighton, Sussex Bn1 9RE England.

Derso A, Bizuneh H, Keleb A, Ademas A, Adane M (2021) Food insecurity status and determinants among Urban Productive Safety Net Program beneficiary households in Addis Ababa, Ethiopia. *PLOS ONE,*16(9): e0256634.https://doi. org/10.1371/journal.pone.0256634

Alemseged G, Hailu B (2018) Determinants of rural household food insecurity in Northern Ethiopia: An empirical study. *Sustainable Development Africa*, 2018; 20(1):32–63.

Scho L, Wekesah F, Mohamed S (2014) Vulnerability to food insecurity in urban slums: Experiences from Nairobi, Kenya. *Journal of Urban Health*, 2014; 91(6):1098–113.

42.Conclusion/recommendation

**Response:** we have included a brief recommendation.

The UPSNP program has proven to be an effective social protection intervention in combating chronic poverty and food insecurity in urban areas of Ethiopia. Its positive impact on asset accumulation, food consumption, and overall well-being of beneficiary households is evident. Therefore, it is recommended to continue and expand the UPSNP program to reach more vulnerable urban households and further improve their livelihoods. By doing so, Ethiopia can make significant strides towards achieving sustainable development and reducing poverty and food insecurity in urban areas.

Response to Reviewers

Based on the questions raised by reviewers, I have responded one by one as follow:

##### Author’s Response

**Question 1.** In the abstract, the authors should do some grammar checks on some tenses (appropriate use of past tense). And indicate the knowledge gap and/ problem statement.

**Response**: I made the necessary grammar correction and the inclusion of the knowledge gap.

**Question 2: Lines 67 & 68 -** An estimated 149 million Africans are facing acute food insecurity— an increase of 12 million people from a year ago. Is this a recent information? If yes please indicate the year of reference and source of the information with appropriate referencing.

**Response:** I include the following reference**:**

Food and Agriculture Organization of the United Nations. (2023). *The State of Food Security and Nutrition in the World 2023*. Rome: FAO.

**Question 3:** Lines 78 & 79 - Before implementing PSNP, the Ethiopian government relied on unpredictable annual appeals for emergency food relief, which saved lives but failed to address the increasing number of food insecure individuals [6].

**Response:** Numbers 69 and 70 are **deleted** because they were written mistakenly.

**Question 4:** Lines 84 to 89 - The study suggests that the PSNP plays a crucial role in ensuring food security for both chronically and temporarily food insecure households. By providing program transfers and supporting productive activities, the PSNP aims to assist over 7.5 million people in 318 districts across eight regions. This targeted approach is likely more effective in addressing the needs of food insecure individuals compared to the unpredictable annual appeals for emergency food relief. No reference to support this fact.

**Response:** I include the following reference**:**

Gilligan, D. O., Hoddinott, J., & Seyoum Taffesse, A. (2008). *The impact of Ethiopia's Productive Safety Net Programme and its linkages***.** International Food Policy Research Institute (IFPRI).

**Question 5:** Lines 90 to 93 - Unlike the initial emergency relief appeal method, which relied on unpredictable annual appeals for food aid, the PSNP operates on a set timeline until December 2014. This longer-term planning allows for more stability and predictability in addressing food insecurity issues. No reference.

**Response:** I include the following reference**:**

Berhane, G., Gilligan, D. O., Hoddinott, J., Kumar, N., & Taffesse, A. S. (2014). Can social protection work in Africa? The impact of Ethiopia's Productive Safety Net Programme. *Economic Development and Cultural Change,* 63(1), 1-26.

**Question 6:** Lines 126 & 127 - Hence, Dessie City has been labeled as typical food insecure area despite various food security interventions with PSNP is effective or not. Please paraphrase the following statement:

**Response:** The statement is paraphrased as follow:

Despite the introduction of multiple food security interventions with PSNP, Dessie City has been classified as a typical food insecure area.

**Question 7:** Lines 134 &135 - Historically, urban poverty has consistently faced the significant challenge of food insecurity, especially in drought-prone areas of Ethiopia. No reference.

**Response:** I include the following reference**:**

Zewdie, Yilma Muluken*. 2014. "Food Insecurity and Coping Strategies of Urban Poor Households in Addis Ababa." Journal of Economics and Sustainable Development 5(20): 20-30.*

**Question 8:** Lines 296-302 - Impact Indicator Variables for Asset Accumulation and consumption

The impact of the PSNP is analyzed by dividing the two categories which are the asset building and consumption expenditure of the PW-PSNP beneficiary and eligible non-beneficiary households. Reference for this asersion.

**Response:** I include the following reference**:** Gebrehiwot, T. and Castilla, C. 2019. "The Role of the Productive Safety Net Programme (PSNP) in Improving Consumption and Asset Accumulation in Rural Ethiopia." *Journal of Development Studies* 55(5): 685-700.

**Question 9: Outcome Variables for Asset Accumulation:** The first is the home asset (which is the sum of household goods and consumer durables) and community asset value is also measured based on the estimation of its market value by respondents in terms of Birr. Reference for this tool/ascersion.

**Response:** I include the following reference**:** Gilligan, Daniel O., and Hoddinott, John. 2007. "Is There Persistence in the Impact of Emergency Food Aid? Evidence on Consumption, Food Security, and Assets in Rural Ethiopia." American Journal of Agricultural Economics 89(2): 225- 242.

**Question 10:** Lines 306 to 310 - Therefore, in the study, the households have been asked about the quantity as well as the current price of overall households mean real food consumption expenditure per Adult Equivalent (AE) in terms of 24 hours and current non-food consumption expenditure per adult equivalent (AE) in terms of year, at their disposal, during the field survey based on the respondents’ consumption rate in terms of Ethiopian Birr. Reference for the source of this measure/tool?

**Response:** I include the following reference**:** Gebrehiwot, Tesfaye, and Van der Veen, Anne. 2015. "Coping with Food Insecurity on a Micro-scale: Evidence from Ethiopian Rural Households**."** Ecology of Food and Nutrition 54(2): 187-208.
