# Supplementary material for: Impact of urban productive safety net program on urban households’ asset accumulation and food consumption rate in Dessie City, South Wollo Zone, Amhara Region, Ethiopia
Source: PLoS One. 2024 Sep 25;19(9):e0308575. doi: 10.1371/journal.pone.0308575 (PMC11423993; doi:10.1371/journal.pone.0308575)
Supplement: Attachment — Submitted filename: Responses to reviewers.docx [file pone.0308575.s006.docx]

Based on the questions raised by reviewers, I have responded one by one as follow:

**Author’s Response**

**Question 1.** In the abstract, the authors should do some grammar checks on some tenses (appropriate use of past tense). And indicate the knowledge gap and/ problem statement.

**Response**: I made the necessary grammar correction and the inclusion of the knowledge gap.

**Question 2: Lines 67 & 68 -** An estimated 149 million Africans are facing acute food insecurity—an increase of 12 million people from a year ago. Is this a recent information? If yes please indicate the year of reference and source of the information with appropriate referencing.

**Response:** **I include the following reference:**

Food and Agriculture Organization of the United Nations. (2023). The State of Food Security and Nutrition in the World 2023. Rome: FAO.

**Question 3:** Lines 78 & 79 - Before implementing PSNP, the Ethiopian government relied on unpredictable annual appeals for emergency food relief, which saved lives but failed to address the increasing number of food insecure individuals [6].

**Response:** Numbers 69 and 70 are **deleted** because they were written mistakenly.

**Question 4:** Lines 84 to 89 - The study suggests that the PSNP plays a crucial role in ensuring food security for both chronically and temporarily food insecure households. By providing program transfers and supporting productive activities, the PSNP aims to assist over 7.5 million people in 318 districts across eight regions. This targeted approach is likely more effective in addressing the needs of food insecure individuals compared to the unpredictable annual appeals for emergency food relief. No reference to support this fact.

**Response: I include the following reference:**

**Gilligan, D. O., Hoddinott, J., & Seyoum Taffesse, A. (2008).** The impact of Ethiopia's Productive Safety Net Programme and its linkages**. International Food Policy Research Institute (IFPRI).**

**Question 5:** Lines 90 to 93 - Unlike the initial emergency relief appeal method, which relied on unpredictable annual appeals for food aid, the PSNP operates on a set timeline until December 2014. This longer-term planning allows for more stability and predictability in addressing food insecurity issues. No reference.

**Response: I include the following reference:**

Berhane, G., Gilligan, D. O., Hoddinott, J., Kumar, N., & Taffesse, A. S. (2014). Can social protection work in Africa? The impact of Ethiopia's Productive Safety Net Programme. *Economic Development and Cultural Change,* 63(1), 1-26.

**Question 6:** Lines 126 & 127 - Hence, Dessie City has been labeled as typical food insecure area despite various food security interventions with PSNP is effective or not. Please paraphrase the following statement:

**Response:** The statement is paraphrased as follow:

Despite the introduction of multiple food security interventions with PSNP, Dessie City has been classified as a typical food insecure area.

**Question 7:** Lines 134 &135 - Historically, urban poverty has consistently faced the significant challenge of food insecurity, especially in drought-prone areas of Ethiopia. No reference.

**Response: I include the following reference:**

Zewdie, Yilma Muluken. 2014. "Food Insecurity and Coping Strategies of Urban Poor Households in Addis Ababa." ***Journal of Economics and Sustainable Development*** 5(20): 20-30.

##### **Question 8:** Lines 296-302 - Impact Indicator Variables for Asset Accumulation and consumption

##### The impact of the PSNP is analyzed by dividing the two categories which are the asset building and consumption expenditure of the PW-PSNP beneficiary and eligible non-beneficiary households. Reference for this asersion.

**Response:**  **I include the following reference:**

Gebrehiwot, T. and Castilla, C. 2019. "The Role of the Productive Safety Net Programme (PSNP) in Improving Consumption and Asset Accumulation in Rural Ethiopia." ***Journal of Development Studies*** 55(5): 685-700.

**Question 9:** **Outcome Variables for Asset Accumulation:** The first is the home asset (which is the sum of household goods and consumer durables) and community asset value is also measured based on the estimation of its market value by respondents in terms of Birr. Reference for this tool/ascersion.

**Response: I include the following reference:**

Gilligan, Daniel O., and Hoddinott, John. 2007. "Is There Persistence in the Impact of Emergency Food Aid? Evidence on Consumption, Food Security, and Assets in Rural Ethiopia." **American Journal of Agricultural Economics** 89(2): 225-242.

**Question 10:** Lines 306 to 310 - Therefore, in the study, the households have been asked about the quantity as well as the current price of overall households mean real food consumption expenditure per Adult Equivalent (AE) in terms of 24 hours and current non-food consumption expenditure per adult equivalent (AE) in terms of year, at their disposal, during the field survey based on the respondents’ consumption rate in terms of Ethiopian Birr. Reference for the source of this measure/tool?

**Response: I include the following reference:**

Gebrehiwot, Tesfaye, and Van der Veen, Anne. 2015. "Coping with Food Insecurity on a Micro-scale: Evidence from Ethiopian Rural Households**." Ecology of Food and Nutrition** 54(2): 187-208.
