# Supplementary material for: Impact of urban productive safety net program on urban households’ asset accumulation and food consumption rate in Dessie City, South Wollo Zone, Amhara Region, Ethiopia
Source: PLoS One. 2024 Sep 25;19(9):e0308575. doi: 10.1371/journal.pone.0308575 (PMC11423993; doi:10.1371/journal.pone.0308575)
Supplement: Attachment — Submitted filename: REVIEWER comment-PONE-D-23-24451.docx [file pone.0308575.s001.docx]

**REVIEWER’S COMMENT**

**Summary**

Chronic poverty, particularly the long-term lack of food, remains a significant issue. Millions of people worldwide faced food insecurity, with a majority residing in developing regions. Ethiopia, in particular, had about 8.5 million chronically food insecure individuals, constituting 10 percent of its population. These individuals lacked consistent access to sufficient food, leading to asset selling and consumption restrictions in order to survive. A study conducted indicated that the second phase of Productive Safety Net Programmed (PSNP) plays a crucial role in ensuring food security for both chronically and temporarily food insecure households in Ethiopia. This program aims to assist over 7.5 million people (1.6 million households) in 318 districts across eight regions by providing program transfers and supporting productive activities until December 2014. Before implementing PSNP, the Ethiopian government relied on unpredictable annual appeals for emergency food relief, which saved lives but failed to address the increasing number of food insecure individuals. The implementation of PSNP was done in an attempt to break the cycle of annual appeal of food aid and achieve an acceptable level of food security at macro (national) and micro (household) level. The PSNP is currently the largest operating social protection program in Sub-Saharan Africa, outside of South Africa. It differs from previous food-for-work programs, in that it focuses continuously on selected household over several years and in that the explicit objective was that it will eventually be phased out. At national level, there were some works that tried to highlights the impacts of program intervention, particularly PSNP. Unfortunately, most of them have focused on the impacts of PSNP on asset building and consumption in rural areas rather than urban areas. Hence, this study aimed at bridging the knowledge gap.

**Major comments**

The introduction, methods, and results, sections are well documented with good clarity and are scientific facts. However, there are major revisions to be done on the manuscript before publication.

**Minor comments**

1. In the abstract, the authors should remove italics from this section.
2. In the introduction, line 53, the name of the author should be mention before the reference number.
3. Lines 59 &60, approximately 850 million people worldwide faced food insecurity, with a majority residing in developing regions.
4. Lines 60 &61, In Africa, around 200 million people, accounting for 27.4 percent of the population, experienced food insecurity between the 1970s and 1990s
5. Lines 61 & 62, Ethiopia, in particular, had about 8.5 million chronically food insecure individuals, constituting 10 percent of its population.
6. Lines 65 &66 conducted a study indicating that the second phase of PSNP plays a crucial role in ensuring food security for both chronically and temporarily food insecure households in Ethiopia.
7. Lines 67 &68 districts across eight regions by providing program .transfers and supporting productive activities until December 2014.
8. Line 82, million urban poor living in 972 cities and towns.
9. Lines 86-88, beneficiaries, In the first phase, 604,000 the poorest 12 percent and about 55 percent of people living below the poverty line in these 11 cities Urban Productive Safety Net Project [10].
10. Line 93, Having these in mind the researcher was try to evaluate the impacts of……………………
11. Line 98 Chronically, food insecurity has been one of the most problems of urban poverty, particularly in drought-prone
12. Line 100, High proportion of food insecure households, which have been receiving emergency food aids.
13. Line 101, Although the emergency assistance was substantial and saved many lives, it was uncertainty of the food aid,
14. Lines 107-109, aim of preventing asset depletion at household level while creating assets at the community level (NO REFERENCE).
15. Lines 112-113, Some findings state that, PSNP has able to smooth the consumption and protects household’s assets (GRAMMER CHECK for clarity).
16. Line 113, [14] indicated that beneficiary households. Mention the authors NAME BEFORE the reference number.
17. Lines 114-115, In contrast to these, what proved is that there is no significant difference between, the consumption as well as assets of PSNP participants and non-participants.
18. Lines 116-118, Moreover, [17], stated that, participation in PSNP reduces the asset size of the participants, due to the dependency behavior of beneficiaries which discourage them to put efforts on their agricultural activities.
19. Line 112, levels have brought inconsistent results and far from conclusive,(This statement is not clear).
20. Line 136, THE RESEARCH DESIGN AND SAMPLING
21. Lines 137-139, Because of survey design helped identify patterns of behavior, activities, people‘s attitude, opinions, habits and any of the variety of social problems that influence the explicability and scaling-up efforts of the program across various contexts
22. Lines 139-141, Descriptive survey design help to identify the issues had been occurred and could be predetermine certain preventive measures could be adopted.
23. Line 144, The quantitative data were analyzed and interpreted in the numerical data through appropriate.
24. Line 154, stated that target population as all members of a real set of subjects to which a researcher wishes to…….
25. Lines 155-156, The study was targeted each selected *Kebele*, the name of the entire list of household heads were obtained from *Kebele* administration office.
26. Line 157-158, Then on the bases of *Kebele* document there are 338 PWPSNP beneficiary households and 272 eligible non-beneficiary households in Robit *Kebele.*
27. Line 160, beneficiary household heads were used to as sample frames included for the survey (The statement needs to be improved upon grammatically).
28. Line 175, In addition to this, 6 key informant interviewees were conducted with purposively.
29. Lines 175-176, ). In addition to this, 6 key informant interviewees were conducted with purposively selected *Kebele* Development Agents, *Kebele* Administrators and head of UPSNP Task Force.
30. Line 178, The study was employed both primary and secondary data sources.
31. Line184, The study was employed both quantitative and qualitative data analysis techniques
32. Lines 186 &187, Similarly, qualitative data was gathered through open-ended questionnaires and interview and analyzed through textually to supplement the questionnaire survey
33. Lines 189 &190, please rephrase
34. Line 190, and percentages were done to …..
35. Lines 193 &194, In addition, to the simple descriptive analysis, to estimate the impact of participation in the urban Productive Safety Net program.
36. Line 198, According to [22], in estimating logit model for the dichotomous (binary) logistic regressions will be used to
37. Line 249, by classifying the households’ consumption in to two main
38. Line 254, the field survey based on the respondents’ their consumption rate in terms of Ethiopian birr.
39. Results and Discussion section (pages 10- 17) should be well written with appropriate comparison with relevant previous research output/published articles and must be well referenced. That is this section should not just be result presentations alone without being adequately discussed.
40. Line 474, CONCLUSION
41. Line 534, (pages 20-21) **REFERENCES**
42. **Conclusion/recommendation**

Generally, the study titled “Impact of Urban Productive Safety Net Program on Urban Households’ Asset Accumulation and Food Consumption Rate in Dessie City, South Wollo Zone, Amhara Region, Ethiopia” was well-researched, very relevant, scientific, and very informative, it raised pertinent issues regarding the impact of government intervention on asset acquisition and food consumption in achieving the “zero hunger” which is the Sustainable Development Goal 2. The authors demonstrated excellent mastery of the study. However, major corrections are required as highlighted.
